# Evaluation of school sports coach position policies in China based on the PMC Index Model

**DOI:** 10.3389/fpubh.2026.1880356

**Published:** 2026-09-18

**Authors:** Hongbo Shi, Xinyu Yu, Dawei Cao, Yuanyuan Gao

**Affiliations:** 1 Graduate School, Harbin Sport University, Harbin, Heilongjiang, China; 2 School of Physical Education, Huaibei Normal University, Huaibei, Anhui, China; 3 School of Physical Education, Harbin Sport University, Harbin, Heilongjiang, China

**Keywords:** content analysis, PMC Index Model, policy design evaluation, position setting, school sports, school sports coaches

## Abstract

**Background:**

In 2023, the General Administration of Sport of China, the Central Institutional Organization Commission Office, the Ministry of Education, and the Ministry of Human Resources and Social Security jointly issued the Implementation Opinions on Establishing Coaching Positions in Schools, positioning school sports coaches as an important mechanism for integrating sports and education. Provincial authorities subsequently introduced supporting measures. This study evaluates the internal structure, completeness, and consistency of these policy documents to identify design strengths and weaknesses and inform future policy refinement.

**Methods:**

Twelve formally promulgated policy documents issued between 2022 and 2024 were selected using explicit inclusion and exclusion criteria. Policy text mining, a two-round Delphi consultation with 15 experts, and content analysis were used to construct a Policy Modeling Consistency (PMC) index model. Nine of ten conceptual first-level variables, comprising 38 s-level indicators, entered the PMC calculation. Policy texts were coded using a documented coding manual by a single coder, with consistency assessed through blind test–retest re-coding. Descriptive statistics and sensitivity analyses examined indicator discrimination and ranking robustness.

**Results:**

The mean PMC index was 6.29 (SD = 0.523; range = 5.57–7.22). Ten policies were classified as good and two as excellent. Policy level (X4) and policy domain (X9) showed zero variance and therefore did not discriminate among policies. Among the seven discriminating variables, mean scores were highest for participating actors (X6 = 0.92) and policy focus (X3 = 0.88), and lowest for policy timeliness (X2 = 0.06), policy evaluation (X8 = 0.73), and guarantee and incentives (X7 = 0.75). Removing X4 and X9 left the ranking unchanged (Spearman ρ = 1.0000), while additionally removing X2 produced only a marginal change (ρ = 0.9926). However, grade classifications varied substantially across specifications.

**Conclusion:**

The policies are comparatively complete across the measured design dimensions but remain weak in timeliness, evaluation, and guarantee and incentive mechanisms. Only three documents specify a policy time horizon, few include verifiable objectives, and supervision mechanisms are often insufficiently detailed. The PMC scores should therefore be interpreted as measures of structural completeness rather than implementation effectiveness, which requires separate implementation and outcome data.

## Introduction

In 2020, the General Administration of Sport of China and the Ministry of Education jointly issued the Opinions on Deepening the Integration of Sports and Education to Promote the Healthy Development of Adolescents (1), which proposed recruiting outstanding retired athletes into schools to serve as physical education teachers and coaches, thereby ensuring an adequate supply of sports coaching personnel in schools. In 2023, the General Administration of Sport of China, the Central Institutional Organization Commission Office, the Ministry of Education, and the Ministry of Human Resources and Social Security jointly promulgated the Implementation Opinions on Establishing Coaching Positions in Schools (2) (hereinafter referred to as the Implementation Opinions). This policy stipulates that schools at all levels, from compulsory education to higher education, should establish corresponding coaching positions. In terms of staffing arrangements, local authorities are encouraged to adopt models suited to regional conditions, including full-time positions incorporated into the official staffing system as well as part-time positions procured through government service purchasing. Retired athletes at or above the first-class level are identified as a key target group for recruitment. The policy further provides that school sports coaches should apply their professional expertise primarily to sports training and competition work in schools (2). How the coach’s role is bounded against the physical education teacher’s is a question that arises in other systems as well (3), and it is returned to in the Discussion.

These documents institutionalize school coaching work within the wider policy agenda of integrating sport and education. They assign coaches responsibilities both for students’ physical and mental development and for the early stages of the competitive sport talent pathway. Subsequently, many provinces and municipalities have introduced supporting implementation measures to ensure that coaching positions in primary and secondary schools are established in accordance with actual needs. Closely aligned with the practical demands of contemporary school sports, these policies address the staffing and governance challenges facing school sport provision in China, and set out the responsibilities attaching to school coaching posts. Coaches working in schools have been shown to contribute to physical activity promotion and youth development (4), and in the Chinese policy framework they are also assigned a role in identifying and developing athletes for the competitive sport system (2). Establishing these posts is therefore intended as a policy instrument for deepening the integration of sport and education, improving school sport provision — described in the policy documents themselves as its “high-quality development” — and giving adolescents access to more systematic professional coaching. Accordingly, examining how these policies are designed — how completely and consistently they specify the arrangements on which implementation depends — can inform the future refinement of coaching-position policy in China. Such an examination addresses policy design; establishing what the policies have achieved in practice would require separate evidence on implementation, which is beyond the scope of the present study.

The provision of qualified coaching personnel in schools is not a challenge specific to China. Comparable policy questions — how coaching expertise is brought into schools, how coaches are credentialled and remunerated, and how school-based provision connects to the wider sport system — arise across national settings, and international scholarship has examined them from several directions: frameworks for lifelong physical activity and sport participation (5), implementation-science approaches to comprehensive school physical activity programs (6), the use of near-peer coaches to combine physical activity promotion with youth development (4), the delivery of coach education where geographic access is constrained (7), and the definition of professional boundaries between physical education teachers and coaching staff (3). What distinguishes the Chinese approach is the choice of policy instrument. Rather than operating principally through certification markets or contracted external providers, the Chinese policy establishes coaching work as a category of post within the public institutional staffing system (bianzhi) and attaches it to the professional-title system. This makes questions of staffing quotas, title evaluation criteria and inter-departmental coordination central to policy design in a way that has no direct counterpart in systems where school coaching is organized contractually. It also means that the design features which matter most for this policy area are institutionally specific, and that an evaluation framework for it must be constructed accordingly.

The model has been applied across a range of public-policy domains, including regional science and technology innovation policy (8). In the field of education policy evaluation, the PMC Index Model has been widely applied to such areas as the promotion of digital transformation in education (9), the development of compulsory education (10), and the advancement of first-class undergraduate education in universities (11), thereby providing a scientific basis for relevant policy development. In recent years, quantitative studies on sports policy evaluation have also shown a growing trend. The application of the PMC Index has gradually expanded from areas such as sports consumption (12), public services for national fitness (13), and the sports industry (14) in the broader field of sport humanities and social sciences to the domain of school sports (15), where it has yielded promising results. Against this backdrop, the present study employs the PMC Index Model to examine 12 national and provincial policy documents concerning the establishment of sports coaching positions in primary schools, secondary schools and higher education institutions. Three gaps motivate the study. First, existing research on school coaching provision has examined coach education, credentialling and delivery models, but rarely the internal design of the policy documents that establish coaching posts. Second, little is known about how a public-sector staffing system of the Chinese kind — in which posts are created within establishment quotas and tied to a professional-title ladder — shapes the design of school coaching policy. Third, applications of the PMC model seldom test whether their indicators discriminate between the policies scored or whether the resulting ranking survives a change of specification. The study accordingly asks three questions: what design features characterize these policies; which design dimensions are consistently strong and which consistently weak across the policy set; and whether the resulting assessment is robust to the specification of the evaluation framework. The unit of analysis is the policy document, and the evidence is the promulgated policy text.

## Materials and methods

### Study design

This study is a quantitative evaluation of policy texts. It applies content analysis and the PMC Index Model to formally promulgated policy documents in order to appraise the internal design, structural completeness and consistency of those documents. Throughout, a higher PMC score is described as indicating a more complete policy design on the dimensions measured, never as indicating a better policy: the index registers what a document specifies, and the relation between design completeness and policy quality is not something a text analysis can establish. The unit of analysis is the policy document. The design is not that of a systematic review: the study does not identify, appraise or synthesize the findings of primary empirical studies, and reporting standards developed for evidence synthesis are correspondingly not applicable. Nor does the design permit inference about implementation outcomes. Policy evaluation of this kind establishes what a policy specifies and how coherently it does so; establishing what a policy achieves requires implementation and outcome data, which this study does not use. Claims throughout are confined accordingly.

### PMC index model

The PMC Index Model, proposed by Ruiz Estrada et al. (16), is commonly used in policy evaluation research. As a model for policy assessment and analysis, it not only examines the specific features of a policy, but also evaluates its internal consistency, thereby identifying existing problems in a targeted manner and offering suggestions for improvement. It addresses both the limited persuasiveness of purely text-based evaluation and the shortcomings of mathematical methods, which often involve substantial subjectivity in indicator selection and insufficient precision (17). The construction of the PMC Index Model generally includes four steps: variable classification and parameter setting, the establishment of a multi-input–output table, PMC index calculation, and PMC surface construction (18).

### Selection of policy texts

#### Retrieval sources

Policy documents were retrieved from four sources: the Central People’s Government portal^1^; the official portals of the General Administration of Sport of China, the Ministry of Education, and the Ministry of Human Resources and Social Security; the official portals of provincial, autonomous-region and municipal sports bureaux, education departments, human-resources and social-security departments, and institutional-organization commission offices; and the PKUlaw database, used to cross-check the existence and current version of documents located through official portals. Search terms were “establishing coaching positions in schools”, “establishment of school sports coach positions”, “sports coach professional and technical posts”, and “retired athletes serving as school physical education teachers/coaches”. Initial retrieval was conducted between October and December 2024; a verification round was conducted in August 2026, in which every retained document was re-obtained from its issuing authority and checked field by field against the bibliographic details reported in Table 1. The 2026 round verified sources and bibliographic detail for the documents already in the sample; it did not extend the sampling period, and policies issued after the 2022–2024 window were therefore not added. The window is part of the study design — it covers the period from the first local documents to the first wave of responses to the 2023 national opinions — and the cross-sectional character of the sample is stated in the Limitations.

**Table 1:** Policy documents on the establishment of school sports coach positions in China included in the analysis (*n* = 12).

No.	Policy title	Document number	Date of issuance	Issuing authority	Administrative level
P1	Implementation Opinions of the General Administration of Sport of China, the Central Institutional Organization Commission Office, the Ministry of Education, and the Ministry of Human Resources and Social Security on Establishing Coaching Positions in Schools	Ti Ren Gui Zi (2023) No. 3	January 16, 2023	General Administration of Sport of China; Central Institutional Organization Commission Office; Ministry of Education; Ministry of Human Resources and Social Security	National
P2	Notice of Anhui Province on Effectively Carrying Out the Selection of School Physical Education Teachers and Sports Coaches from Retired Athletes of Outstanding Provincial Sports Teams	Wan Ti Ren (2022) No. 9	January 18, 2022 (published February 8, 2022)	Anhui Provincial Sports Bureau; Provincial Department of Education; Provincial Department of Human Resources and Social Security	Provincial
P3	Implementation Measures of Fujian Province for Establishing Coaching Positions in Schools	Not stated in available text	April 14, 2023	Fujian Provincial Sports Bureau; Provincial Institutional Organization Commission Office; Provincial Department of Education; Provincial Department of Human Resources and Social Security	Provincial
P4	Implementation Rules of Guangdong Province on Establishing Sports Coach Positions in Primary, Secondary, and Higher Education Institutions across the Province	Yue Ti Gui (2024) No. 1	January 15, 2024 (effective February 20, 2024)	Guangdong Provincial Sports Bureau; Provincial Institutional Organization Commission Office; Provincial Department of Education; Provincial Department of Human Resources and Social Security	Provincial
P7	Implementation Opinions of Jiangsu Province on Establishing Coaching Positions in Schools	Su Ti Gui (2023) No. 2	August 29, 2023 (effective September 8, 2023)	Jiangsu Provincial Sports Bureau; Provincial Institutional Organization Commission Office; Provincial Department of Education; Provincial Department of Human Resources and Social Security	Provincial
P8	Implementation Opinions on the Management of Professional and Technical Posts for Sports Coaches in Primary, Secondary, and Higher Education Institutions in Zhejiang Province	Zhe Ti Ren (2022) No. 275	October 12, 2022 (effective December 1, 2022)	Zhejiang Provincial Sports Bureau; Provincial Institutional Organization Commission Office; Provincial Department of Education; Provincial Department of Human Resources and Social Security	Provincial
P9	Implementation Opinions on Establishing Professional and Technical Posts for Sports Coaches in Schools in Jiangxi Province	Not stated in available text	July 25, 2024	Jiangxi Provincial Sports Bureau; Provincial Institutional Organization Commission Office; Provincial Department of Education; Provincial Department of Human Resources and Social Security	Provincial
P12	Implementation Opinions on the Management of Coaching Positions in Primary, Secondary, and Higher Education Institutions in Henan Province	Not stated in available text	August 1, 2023 (published August 16, 2023)	Henan Provincial Sports Bureau; Provincial Institutional Organization Commission Office; Provincial Department of Education; Provincial Department of Human Resources and Social Security	Provincial
P13	Implementation Measures for the Management of Professional and Technical Posts for Sports Coaches in Primary, Secondary, and Higher Education Institutions in Jilin Province	Not stated in available text	August 25, 2023 (published September 13, 2023)	Jilin Provincial Sports Bureau; Provincial Institutional Organization Commission Office; Provincial Department of Education; Provincial Department of Human Resources and Social Security	Provincial
P15	Implementation Opinions of the Inner Mongolia Autonomous Region on Establishing Professional and Technical Posts for Sports Coaches in Primary, Secondary, and Higher Education Institutions	Not stated in available text	December 21, 2023	Inner Mongolia Autonomous Region Sports Bureau; Regional Institutional Organization Commission Office; Regional Department of Education; Regional Department of Human Resources and Social Security	Provincial (Autonomous Region)
P17	Implementation Plan of Chongqing Municipality for Establishing Coaching Positions in Primary, Secondary, and Higher Education Institutions	Not stated in available text	August 29, 2023	Chongqing Municipal Sports Bureau; Municipal Institutional Organization Commission Office; Municipal Education Commission; Municipal Human Resources and Social Security Bureau	Provincial (Municipality)
P18	Implementation Opinions on the Management of Professional and Technical Posts for School Coaches in Shanghai	Hu Ti Ren (2024) No. 82	April 30, 2024 (published May 8, 2024)	Shanghai Municipal Sports Bureau; Municipal Institutional Organization Commission Office; Municipal Education Commission; Municipal Human Resources and Social Security Bureau	Provincial (Municipality)

Administrative level is coded under the revised definition of X4 (see Methods). Municipalities directly under central government and autonomous regions occupy the same administrative level as provinces and are coded as provincial, with the sub-type given in parentheses. Where the promulgated text carries a signature date and the issuing portal a later publication date, the signature date is given first and the publication date in parentheses; where the document specifies its own commencement date, that date is given in parentheses instead. Document numbers are given in romanized form and are recorded where they appear in the obtained promulgated text; a document number is not an inclusion criterion, since some authorities publish the text of a policy on a portal page that omits it. “Not stated in available text” indicates that the obtained version carries no document number of its own, which occurs where a policy was retrieved as a web page rather than as the issued document; it does not indicate that the document lacks one. The original Chinese titles, document numbers and full issuing-agency lists are reported in Supplementary material S3. Six documents retrieved during sampling were excluded for failing the inclusion criteria; the identification and screening process is reported in Figure 1.

#### Inclusion criteria

A document was included only if it satisfied all four of the following conditions: (i) it is a formally promulgated policy document issued by an identifiable government body or department, with a verifiable issuing date and a full official text, and not superseded or withdrawn at the time of retrieval; (ii) it was issued between 2022 and 2024; (iii) it is directly concerned with the establishment or management of school sports coaching positions, rather than referring to them incidentally; and (iv) its full official text could be obtained from the issuing authority or an authoritative database.

#### Exclusion criteria

A document was excluded if it was a news report or media summary of a policy; a policy interpretation, Q&A or explanatory reading; a reply to a legislative suggestion or proposal; a draft issued for public comment and not formally promulgated; a document concerning a related but distinct subject, in particular professional-title evaluation standards rather than position establishment; a duplicate of a document already included; or a document whose full official text could not be obtained.

#### Screening outcome

Applying these criteria yielded a final sample of 12 policy documents (Table 1): one national document issued jointly by four central departments, eight provincial documents, one autonomous-region document and two documents issued by municipalities directly under central government. Autonomous regions and such municipalities occupy the same administrative level as provinces, so eleven of the twelve documents are provincial-level in that sense; the three types are distinguished here because the analysis treats them as separate cases. Of the documents initially retrieved, six were excluded on the following grounds. Two existed only as drafts for public comment. One was a professional-title evaluation standard rather than a position-establishment policy, and was additionally issued as a draft outside the study window. One was available only as a policy interpretation republished by a municipal authority. One was available only as a news summary accompanied by a reply to a legislative suggestion. Two could not be obtained in full official text from the issuing authority or from the supplementary database. Because coding requires the promulgated text itself, none of these documents could be assessed on the same evidentiary basis as the retained sample, and all were therefore removed from both the scored sample and the text corpus. The identification, screening and inclusion process is set out in Figure 1, and the retrieval record for each document is reported in Supplementary material S3.

**Figure 1:**
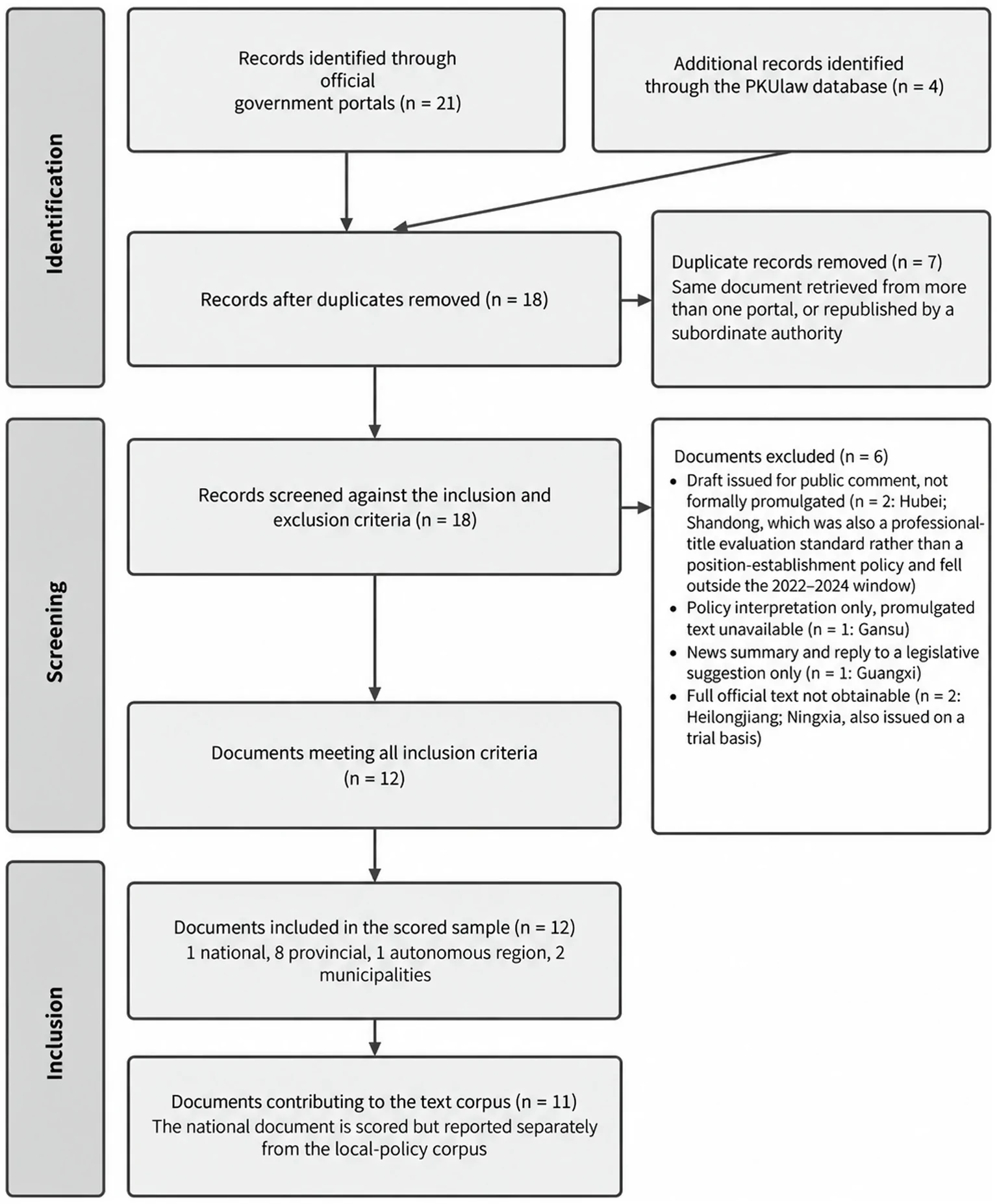
Identification, screening and inclusion of policy documents. Records were retrieved from the four sources listed in the Methods and screened against the inclusion and exclusion criteria stated there. The twelve retained documents comprise one national document, eight provincial documents, one autonomous-region document and two issued by municipalities directly under central government; eleven of them, all but the national document, form the text corpus. Counts are documents, not web pages; a document located through more than one source was counted once. The numbering of the exclusion criteria follows the order in which they are stated in the Methods.

#### Chronology of the sample

The national implementation opinions (P1) were issued in January 2023. Nine of the eleven local documents cite P1 by document number as the authority under which they are issued and were promulgated after it. Two — P2 (Anhui, January 2022) and P8 (Zhejiang, October 2022) — precede it, and cite the 2020 central documents on school physical education and on integrating sport and education instead. The sample is therefore not a uniform set of downstream responses to a single central mandate, and this distinction is drawn on in the Discussion.

#### Consequences for coverage

The final sample covers publicly obtainable formal policies rather than the complete population of policy documents in this area. Two consequences should be noted. First, because sub-provincial implementation rules are unevenly published, the sample is weighted toward national and provincial documents. Second, only one autonomous-region document remains in the sample, so the study cannot support comparison between autonomous regions. Both limitations are revisited in the Limitations section.

### Policy text mining

#### Corpus construction and preprocessing

The PMC scoring sample contains 12 policies: the national framework document P1 and eleven local implementation policies. The word-frequency and semantic-network analyses were defined on the eleven local implementation policies because this stage was used to characterize the shared vocabulary and thematic structure of local implementation documents, whereas P1 was retained in the PMC analysis so that the national framework and local policies could be evaluated on the same indicator system. The text-mining corpus and the PMC scoring sample therefore differ by P1 and serve distinct analytical purposes. The promulgated texts of the eleven local policies were assembled into the text-mining corpus. Because policy documents retrieved from government web portals carry substantial non-policy material, a documented preprocessing pipeline was applied before analysis. The pipeline performed deduplication within each document; separation of interpretive material (policy interpretations, Q&A items, replies to legislative suggestions and press material) into a quarantined file excluded from analysis; removal of web-page furniture (navigation menus, index numbers, font and print controls, view counters, source URLs and capture dates); stripping of HTML tags and entities, URLs, e-mail addresses, control characters and emoji; and normalization of full-width characters to half-width. Portal navigation menus were removed by structural signature rather than by keyword, because menu labels such as “policy interpretation” and “competitive sport” are lexically indistinguishable from policy vocabulary and keyword deletion would distort the frequency counts. Deduplication was confined to within-document repetitions because provincial implementation policies may legitimately reproduce provisions from the same central policy. The final pipeline retained all eleven local policies, removed six duplicate paragraphs, separated 9,441 characters of interpretive text, normalized 2,251 full-width characters, removed 47 items of web furniture and 49 navigation lines, and yielded 30,135 characters of cleaned policy text. Per-document cleaning statistics, the quarantined material and the preprocessing log are reported in Supplementary material S3.

#### Word frequency and semantic network analysis

The cleaned local-policy corpus was segmented and term frequencies were counted using a scripted segmentation and co-occurrence pipeline supplied with Supplementary material S3. A domain dictionary was used so that compound terms specific to this policy area — including school sport, sports coach, coaching position, professional and technical post, and retired athlete — were counted as single terms rather than split into their components. Non-specific terms were screened out and the highest-frequency substantive terms were extracted (Table 2). A semantic-network map was then constructed from co-occurrence within the same policy provision to display the thematic structure of the local-policy corpus and the connections among its principal concepts (Figure 2). The corpus file, screening list, domain dictionary, segmentation rules and co-occurrence rule are provided in Supplementary material S3 so that both Table 2 and Figure 2 can be reproduced.

**Table 2:** Highest-frequency substantive terms in the cleaned policy corpus.

No.	Term	Chinese term	Frequency	No.	Term	Chinese term	Frequency
1	School	学校	285	11	Athletic activity	运动	68
2	School sport	学校体育	262	12	Management	管理	67
3	Coach	教练员	249	13	Professional title	职称	67
4	Sport	体育	209	14	Retired athlete	退役运动员	61
5	Coaching position	教练员岗位	124	15	Post establishment	岗位设置	61
6	Education	教育	85	16	Adolescents	青少年	52
7	Establishment of posts	设置	80	17	Reserve sports talent	体育后备人才	51
8	Sports team	运动队	71	18	Training	培训	49
9	Sports coach	体育教练员	69	19	Government department	部门	47
10	Students	学生	69	20	Sport discipline	项目	46

Frequencies are segmentation counts from the cleaned corpus of the eleven retained local implementation policies (30,135 characters); the national framework document P1 is included in the 12-policy PMC scoring sample but not in this local-policy text-mining corpus, as explained in the Methods. The corpus contains promulgated policy text only and excludes interpretive material. Terms are shown in English translation together with the segmented Chinese term. Each character span belongs to one segmented term, so a shorter term is not counted again inside a longer compound expression. Non-specific terms — function words, general verbs, adjectives and quantifier phrases — were screened out after segmentation; the screening list is provided in Supplementary material S3. The corpus file, counting script and full frequency list are supplied with Supplementary material S3.

**Figure 2:**
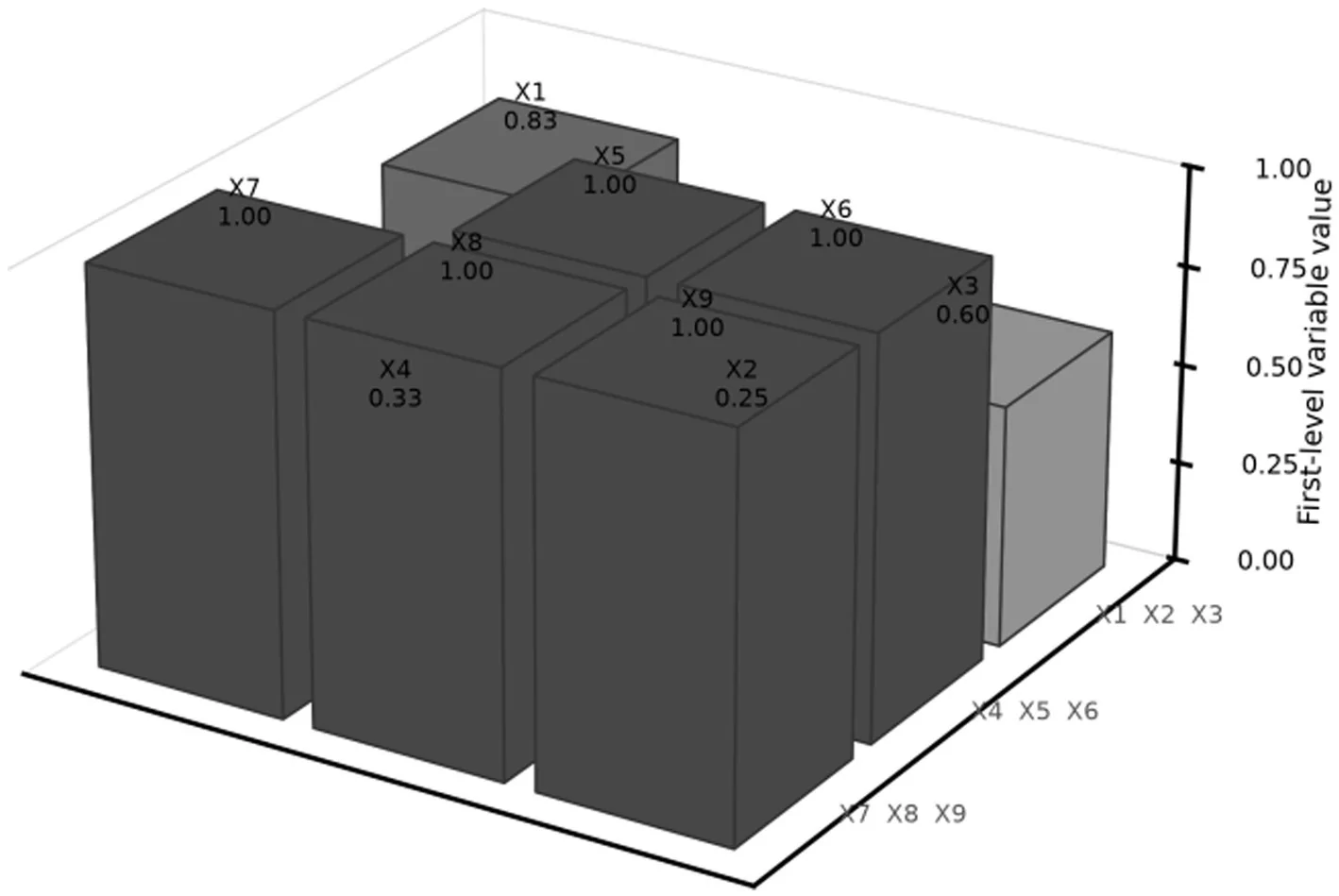
Semantic network map of the local-policy corpus. Nodes are substantive terms and edges indicate co-occurrence within the same policy provision; node size is proportional to term frequency. The map is computed from the same eleven local implementation policies used for Table 2 and is intended to characterize their shared thematic structure; the national framework document P1 is part of the PMC scoring sample but not this text-mining corpus. The map was used to check whether the candidate indicators covered the principal content of the local policies, not to generate the indicators. Edges are shown when two terms co-occur in at least 30 provisions so that the network remains legible. The co-occurrence unit, threshold and plotting script are documented in Supplementary material S3.7.1.

## Empirical analysis of the quantitative evaluation of school sports coach position policies in China

### Variable selection and parameter setting

#### Construction of the indicator framework

Candidate evaluation indicators were drawn from two sources: prior applications of the PMC Index Model in education and sport policy evaluation (18–20), and close reading of the policy corpus. Word-frequency analysis has also been used to characterize Chinese vocational-education policy texts (23). The word-frequency and semantic-network analyses were used to characterize the thematic emphases of the corpus and to verify that the candidate indicators covered its principal content; they were not used to generate the indicators, since term frequency indicates what policy texts discuss most often rather than which design features an evaluation should assess. The final indicator set was determined through a two-round Delphi consultation.

#### Delphi consultation

A panel of 15 experts was constituted, comprising nine university-based experts (professors and associate professors) specializing in physical education teaching and training, sports event organization and management, the integration of sports and education, and athlete psychology, together with six front-line practitioners (senior and first-class school physical education teachers, several holding coaching or national refereeing credentials). Two panel members are retired athletes, one in each group. Two rounds of Delphi consultation were conducted, with a response rate of 100% in both rounds. Indicator retention was judged from the arithmetic mean of expert ratings (concentration of opinion), the coefficient of variation (dispersion), and Kendall’s coefficient of concordance W (coordination), considered together with the panel’s written comments on each indicator. No single numerical cut-off was pre-specified as an automatic retention rule; statistical coordination was treated as acceptable when Kendall’s W was significant at *p* < 0.05. Between rounds, mean ratings increased from 3.800–4.733 to 4.000–4.933 for first-level indicators and from 3.800–4.800 to 4.067–4.933 for second-level indicators; coefficients of variation narrowed from 0.097–0.227 to 0.052–0.217 at the first level; and Kendall’s W increased from 0.382 to 0.430 at the first level and from 0.373 to 0.489 at the second level, with *p* < 0.001 in both rounds. The expert authority coefficient was Cr = 0.883, with familiarity coefficient Cs = 0.827 and judgment-basis coefficient Ca = 0.940, above the conventional threshold of 0.70. The complete Delphi procedure, including the panel roster, round-by-round response data and statistical tables, is reported in Supplementary material S1.

The consultation yielded 10 first-level variables (Table 3). Nine of them are decomposed into second-level indicators, 38 in total, and these nine enter the PMC computation. The tenth, X10 (policy disclosure), has no second-level indicators and is excluded on four grounds. First, it is not measurable independently of the sampling frame: because the inclusion criteria require that a document’s full official text be publicly obtainable, every included document satisfies X10 by construction, and excluded documents cannot be assessed on it at all. Second, and following from this, X10 would contribute a constant to every policy’s index, raising absolute scores without contributing to the comparison between policies. Third, policy disclosure is a property of how a document is promulgated rather than of what it specifies, whereas the other nine variables all assess the internal design of the policy instrument; aggregating a procedural fact about publication with substantive design features into a single index conflates two different categories of information. Fourth, and most simply, X10 was never decomposed into second-level indicators, so there is nothing to code: a first-level value is computed as the mean of its second-level indicators, and for X10 that mean is undefined. X10 is listed in Table 3 as part of the conceptual framework, with its exclusion from the computation recorded in the row itself and in the table note. All counts of second-level indicators in this article — 38 throughout — refer to the nine computed variables.

**Table 3:** First-level and second-level indicators of the school sports coach position policy evaluation framework.

First-level variable	Second-level variables	Source or basis
X1 Policy Nature	X1:1 Forecasting; X1:2 Regulation; X1:3 Recommendation; X1:4 Description; X1:5 Guidance; X1:6 Others	Adapted from Zhang and Qie (20) and related studies, and determined through the Delphi consultation
X2 Policy Timeliness	X2:1 Short-term; X2:2 Medium-term; X2:3 Long-term; X2:4 Within the Current Year	Adapted from Zhang and Qie (20) and related studies, and determined through the Delphi consultation
X3 Policy Focus	X3:1 Qualification Requirements; X3:2 Scope of Application; X3:3 Professional Title System; X3:4 Job Responsibilities; X3:5 Implementation Measures	Drawn from prior PMC applications and close reading of the policy corpus, and determined through the Delphi consultation
X4 Policy Level	X4:1 National; X4:2 Provincial (including autonomous regions and municipalities directly under central government); X4:3 Sub-provincial	Drawn from prior PMC applications and close reading of the policy corpus, and determined through the Delphi consultation
X5 Policy Functions	X5:1 Strengthening; X5:2 Guiding; X5:3 Encouraging; X5:4 Improving; X5:5 Carrying Out; X5:6 Promoting	Drawn from prior PMC applications and close reading of the policy corpus, and determined through the Delphi consultation
X6 Participating Actors	X6:1 Government; X6:2 Schools; X6:3 Society	Drawn from prior PMC applications and close reading of the policy corpus, and determined through the Delphi consultation
X7 Guarantee and Incentives	X7:1 Supervision and Inspection; X7:2 Organizational Leadership; X7:3 Publicity and Guidance; X7:4 Talent Support; X7:5 Others	Drawn from prior PMC applications and close reading of the policy corpus, and determined through the Delphi consultation
X8 Policy Evaluation	X8:1 Clear Objectives; X8:2 Sufficient Basis; X8:3 Scientific Plan; X8:4 Detailed Content	Adapted from Zhang and Geng (8) and related studies, and determined through the Delphi consultation
X9 Policy Domain	X9:1 Education; X9:2 Sports	Adapted from Cai et al. (19) and related studies, and determined through the Delphi consultation
X10 Policy Disclosure	No second-level indicators specified	Retained in the conceptual framework; excluded from the PMC computation (see Methods and the table note)

The framework comprises 10 first-level variables. The nine that are decomposed into second-level indicators — 38 in total — enter the PMC computation. X10 (policy disclosure) has no second-level indicators and is excluded from the computation: it is not measurable independently of the sampling frame, since the inclusion criteria require that every retained document be publicly obtainable; it would contribute a constant to every index; and it describes how a document is promulgated rather than what it specifies (see Methods). It is listed here so that the framework is reported in full. X4 was redefined during revision on the single dimension of the administrative level of the issuing authority, replacing a formulation that conflated administrative rank with geographic scope and could not classify national-level documents. The indicator set was finalized through a two-round Delphi consultation with 15 experts, reported in full in Supplementary material S1; the coding rule for each indicator is given in Supplementary material S2.

#### Revised definition of X4 (policy level)

In its original formulation this variable conflated two distinct dimensions — the administrative rank of the issuing authority and the geographic scope of application — and its categories could not classify national-level documents. It has been redefined on a single dimension, the administrative level of the issuing authority, with three ordered categories: national (issued by central departments), provincial (issued by provincial, autonomous-region or municipality-level authorities), and sub-provincial (issued by prefecture-, city- or district-level authorities). Where a document is issued jointly by several authorities at the same level, the level of that group applies. This definition classifies every document in the sample without ambiguity. All X4 values reported in Table 4 were coded under the revised definition.

**Table 4:** PMC index, concavity index and rank of the 12 policies, with descriptive statistics for each first-level variable.

Sample	X1	X2	X3	X4	X5	X6	X7	X8	X9	PMC index	Concavity index	Rank	Evaluation level
P15	0.83	0.25	0.80	0.33	1.00	1.00	1.00	1.00	1.00	7.22	1.78	1	Excellent
P8	0.83	0.25	0.60	0.33	1.00	1.00	1.00	1.00	1.00	7.02	1.98	2	Excellent
P18	0.83	0.00	1.00	0.33	1.00	1.00	0.80	0.75	1.00	6.72	2.28	3	Good
P4	0.83	0.00	0.80	0.33	0.83	1.00	1.00	0.75	1.00	6.55	2.45	4	Good
P13	0.83	0.25	0.80	0.33	0.83	0.67	0.80	1.00	1.00	6.52	2.48	5	Good
P9	0.83	0.00	1.00	0.33	1.00	1.00	0.80	0.50	1.00	6.47	2.53	6	Good
P3	0.83	0.00	1.00	0.33	0.67	1.00	0.80	0.50	1.00	6.13	2.87	7	Good
P2	0.83	0.00	0.80	0.33	0.83	0.67	0.80	0.75	1.00	6.02	2.98	8	Good
P7	0.83	0.00	1.00	0.33	0.67	1.00	0.60	0.50	1.00	5.93	3.07	9	Good
P12	0.50	0.00	0.80	0.33	0.67	1.00	0.60	0.75	1.00	5.65	3.35	10	Good
P17	0.50	0.00	1.00	0.33	1.00	0.67	0.40	0.75	1.00	5.65	3.35	10	Good
P1	0.67	0.00	1.00	0.33	0.67	1.00	0.40	0.50	1.00	5.57	3.43	12	Good
Mean	0.76	0.06	0.88	0.33	0.85	0.92	0.75	0.73	1.00	6.29	2.71		
SD	0.127	0.108	0.128	0.000	0.144	0.144	0.202	0.190	0.000	0.523	0.523		
Min	0.50	0.00	0.60	0.33	0.67	0.67	0.40	0.50	1.00	5.57	1.78		
Max	0.83	0.25	1.00	0.33	1.00	1.00	1.00	1.00	1.00	7.22	3.43		

X1 policy nature; X2 policy timeliness; X3 policy focus; X4 policy level; X5 policy functions; X6 participating actors; X7 guarantee and incentives; X8 policy evaluation; X9 policy domain. PMC index = ΣX1–X9; concavity index = 9 − PMC. Grading follows the thresholds adopted in the Methods: 5.00–6.99 good, 7.00–8.99 excellent. Policies are ordered by PMC index; P12 and P17 are tied at 5.65 and share rank 10. X4 and X9 have zero standard deviation and therefore no discriminating power in this sample; the consequences are examined in the Results and tested in Table 5. X2 retains two distinct values and so is not constant, but its mean of 0.06 is a substantive finding about the corpus rather than a coding gap: only P8, P13 and P15 state a horizon for their own arrangements, in each case the 14th Five-Year Plan period. All X2 and X4 values were coded under the revised definitions of those variables (Supplementary material S2) and are not comparable with values computed under the original definitions.

### Calculation of the PMC index

#### Coding procedure

Second-level indicators were coded binary (1/0): a value of 1 was assigned where the policy text contains content satisfying the indicator, and 0 otherwise. Coding was governed by a written coding manual (Supplementary material S2) specifying, for each of the 38 indicators, the decision rule for assigning 1 rather than 0. Three conventions were applied throughout. First, the coding unit is the promulgated policy text alone; interpretive documents, news reports and replies to legislative suggestions were excluded from the coding evidence. Second, every code of 1 required a recorded text locator — the article number and the quoted phrase supporting it — so that each assignment is auditable. Every positive code was checked against the final corpus after adjudication: each supporting quotation was required to appear verbatim in the policy it is attributed to. All 321 pass. The matching procedure and the per-document results are reported in Supplementary material S4. Third, genuinely ambiguous content was coded 0 and flagged for adjudication rather than resolved in favor of the higher score, on the reasoning that the opposite convention systematically inflates scores across a sample. Coding was carried out by a single coder. Reliability was therefore assessed by blind re-coding rather than by independent double coding: the coder re-coded all 12 documents on all 38 indicators from the manual at an interval from the original coding, working from the policy texts alone and without reference to the earlier coding sheet or to the previously computed values, and the two passes were compared cell by cell. Agreement is reported as intra-coder (test–retest) consistency. Inconsistencies between passes were resolved by re-reading the policy text against the manual; the pre-adjudication statistics are the reliability estimate and the post-adjudication codes constitute the analyzed dataset. At the item level, agreement was 0.9100 across the indicators whose decision rules were unchanged between the passes. Two variables were coded under rules revised between passes — X2, for which the revised manual excludes commencement clauses, validity periods, individual tenure conditions and standing annual obligations, and X4, redefined as a strict administrative hierarchy — so their disagreements measure the rule change rather than coder inconsistency, and agreement across the full nine-variable set was correspondingly lower at 0.8414. Cohen’s κ is not interpretable for these data: the first-pass marginal that can be reconstructed from the original coding is degenerate, which forces expected agreement to equal observed agreement and κ to zero however closely the passes actually agree. Percentage agreement is therefore the informative statistic available for this design. The derivation, the cell-level comparison and the agreement figures computed at each level of aggregation are reported in Supplementary material S2. Test–retest consistency establishes that the decision rules are specified precisely enough to be reapplied reproducibly, but it does not establish inter-coder reliability, since a consistently held misreading of a rule would recur in both passes; this constraint is stated in the Limitations. To make the coding externally checkable rather than resting on the reliability coefficient alone, the complete item-level coding sheet — the value assigned to each of the 456 policy-by-indicator cells, with the article locator and quoted phrase supporting every code of 1 — is published as Supplementary material S4. First-level variable values were then computed as the unweighted mean of the corresponding second-level indicators. The PMC index was then obtained by summing all first-level variables according to Equations 1–4, where i denotes the first-level variables and j denotes the second-level variables. Subsequently, the S index (degree of concavity) was calculated using Equation 5. The grading criteria were established with reference to the policy evaluation standards proposed by Chen (21). According to this classification, scores ranging from 5.00 to 6.99 indicate a good level, while scores ranging from 7.00 to 8.99 indicate an excellent level (Table 4).

#### Discrimination check

Before interpreting the results, each first-level variable was examined for discriminating power by computing its mean, standard deviation, minimum and maximum across the sample (reported in Table 4). A variable whose values show zero or negligible variance contributes a constant to every policy’s index: it raises absolute scores without differentiating between policies, and any interpretation resting on it therefore describes the indicator rather than the policies. Variables identified in this way are reported explicitly and their contribution to the aggregate quantified.

#### Sensitivity analysis

Because the PMC index weights all first-level variables equally by construction, the resulting ranking may depend on which variables are included. To test this, the index and the ranking were recomputed under five alternative specifications, each omitting one or more variables identified by the discrimination check or affected by the revised X4 definition. Each alternative ranking was compared with the baseline using Spearman’s rank correlation coefficient. Grading thresholds were rescaled in proportion to the number of retained variables, each threshold being multiplied by k/9 where k is the number of first-level variables retained in the specification, so that grade assignments remain comparable across specifications. Results are reported in Table 5.

X∼N[0,1] (1)

X={XR:[0∼1]} (2)

$$X_{i}(\sum_{j=1}^{n}\frac{X_{\mathrm{ij}}}{n(X_{\mathrm{ij}})})$$ (3)

$$\mathrm{PMC}=[\begin{array}{l} X_{1}(\sum_{i=1}^{6}\frac{X_{1i}}{6})+X_{2}(\sum_{i=1}^{4}\frac{X_{2i}}{4})+X_{3}(\sum_{i=1}^{5}\frac{X_{3i}}{5})+\\ X_{4}(\sum_{i=1}^{3}\frac{X_{4i}}{3})+X_{5}(\sum_{i=1}^{6}\frac{X_{5i}}{6})+X_{6}(\sum_{i=1}^{3}\frac{X_{6i}}{3})+\\ X_{7}(\sum_{i=1}^{5}\frac{X_{7i}}{5})+X_{8}(\sum_{i=1}^{4}\frac{X_{8i}}{4})+X_{9}(\sum_{i=1}^{2}\frac{X_{9i}}{2}) \end{array}]$$ (4)

S=9−PMC (5)

**Table 5:** Sensitivity of the policy ranking to the specification of the indicator framework.

Scenario	Specification	Variables retained	Spearman ρ with baseline	Excellent	Good
Baseline	Full nine-variable specification	9	—	2	10
S1	X4 (policy level) removed — zero variance	8	1.0000	3	9
S2	X9 (policy domain) removed — zero variance	8	1.0000	0	12
S3	X4 and X9 both removed	7	1.0000	2	10
S4	X2 (policy timeliness) removed — lowest mean, narrow range	8	0.9926	6	6
S5	X2, X4 and X9 all removed	6	0.9926	8	4

Each scenario recomputes the PMC index and rank after omitting the stated variables. Grade thresholds are rescaled by k/9, where k is the number of retained first-level variables, and Spearman’s ρ compares each scenario with the baseline ranking. X4 and X9 have zero variance in the baseline sample, while X2 has limited dispersion. The unchanged or near-unchanged rank correlations show that the relative ranking is robust, whereas the grade distribution is specification-sensitive.

### PMC surface construction and visualization

Based on the PMC index (22), a model surface was constructed, with the nine first-level indicators arranged into a 3 × 3 PMC surface matrix (Figure 3).

**Figure 3:**
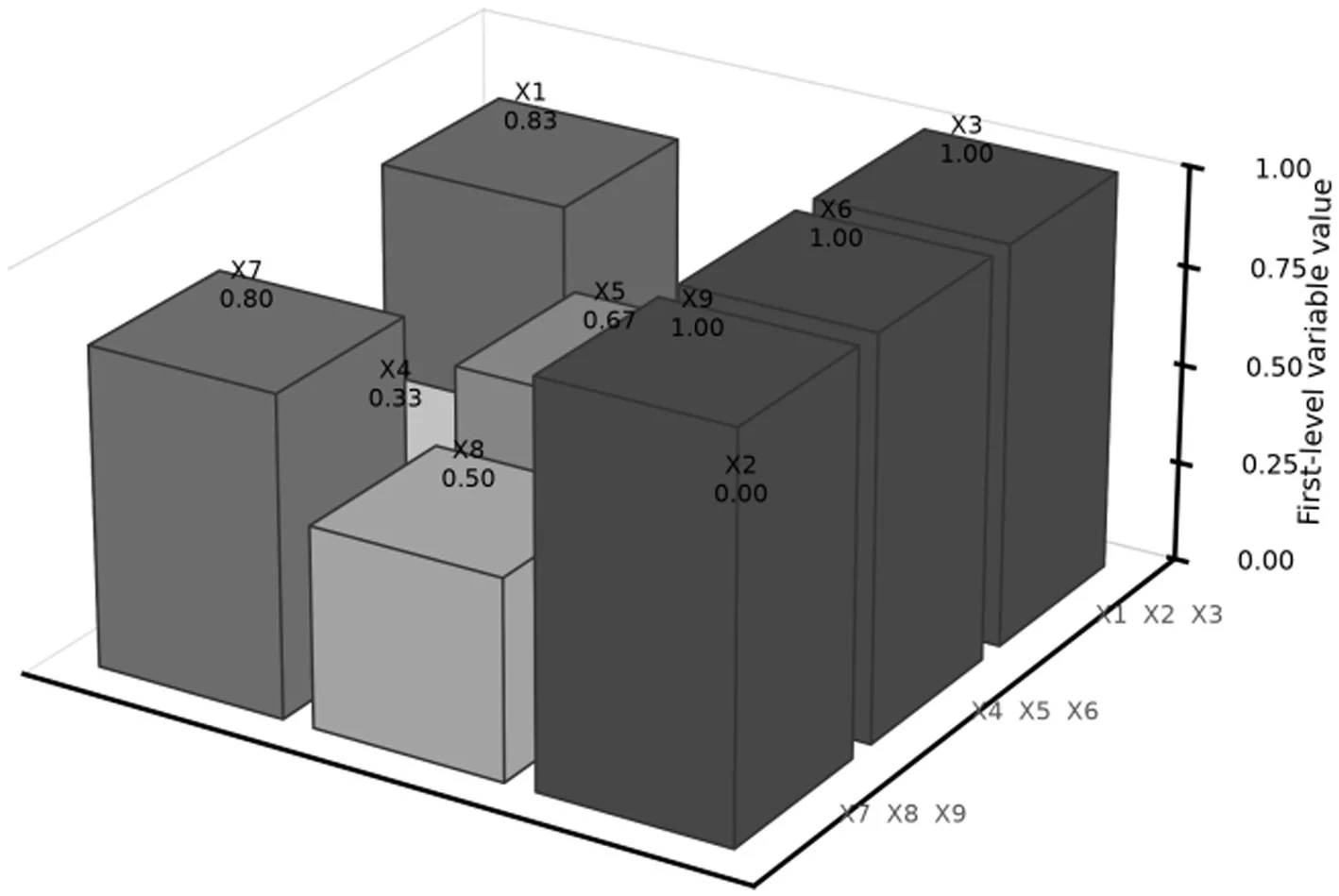
Arrangement of the nine first-level variables in the 3 × 3 PMC surface matrix. The arrangement is identical in every surface diagram, so that a depression always occupies the same position for the same variable and surfaces may be compared directly against one another.

## Results of the quantitative evaluation of school sports coach position policies in China

The PMC indices of the 12 policies were ranked and the mean values of the first-level variables examined in order to identify design features common to the policy set. Seven policies were then selected for closer analysis — the national document (P1), the autonomous-region document (P15), the two municipality documents (P18, P17) and three provincial documents spanning the range of provincial scores (P8, the highest-scoring document in the sample; P3, mid-ranked; and P7, in the lower quartile) — in order to show how variable content is configured at different administrative levels and at different points in the score distribution.

### Macro-level analysis

The 12 policies achieved a mean PMC index of 6.29 (SD 0.523), ranging from 5.57 to 7.22. Ten fall in the good band; only P15 (Inner Mongolia, 7.22) and P8 (Zhejiang, 7.02) reach the excellent band under the thresholds adopted here, and the national four-ministry document P1 scores lowest at 5.57. Read at face value, this distribution indicates a moderate and rather uniform standard of policy design. It should be read with two qualifications, however. First, ten of the twelve policies sit in a single grade band and the two above the threshold clear it by 0.22 and 0.02 points, so the excellent/good classification separates almost nothing, and the analysis below therefore rests on the variable-level profile rather than on the grade. The concavity index is reported alongside the PMC index for comparability with other PMC studies, but by Equation 5 it is a linear transformation of the index and carries no information the index does not; it is not treated here as separate evidence. Second, the PMC index registers whether a policy addresses a given design dimension, not how stringently, specifically or feasibly it does so: a document referring to supervision in general terms scores identically to one that specifies an inspection mechanism with defined consequences. The scores accordingly speak to structural completeness of policy design, and should not be read as evidence about implementation.

At the variable level, the highest mean scores were recorded for policy domain (X9, 1.00), participating actors (X6, 0.92) and policy focus (X3, 0.88), followed by policy functions (X5, 0.85) and policy nature (X1, 0.76). Radar charts of the nine variables for the policies discussed individually are given in Figure 4. Every document in the sample is issued jointly by sports and education authorities and addresses both domains, delimits its focus to post setting, qualification, appointment and title assessment, and names the departments and schools responsible. The lowest mean scores were recorded for policy timeliness (X2, 0.06), policy level (X4, 0.33), policy evaluation (X8, 0.73) and guarantee and incentives (X7, 0.75). X4 is not a substantive finding: its three indicators are mutually exclusive by construction, so each document satisfies exactly one of the three and necessarily contributes 1/3 to the variable. X4 therefore has no discriminating power in this specification of the PMC model, and would have none in any sample coded under a mutually exclusive rule — the constancy follows from the variable’s structure, not from the composition of this sample (see the discrimination check below). The substantive weaknesses of the policy set are the remaining three: nine of the twelve documents attach no time horizon at all to their own arrangements, few state an objective with a verifiable time point, quantity or coverage or set out a staged implementation plan, and supervision and incentive mechanisms are weakly specified.

**Figure 4:**
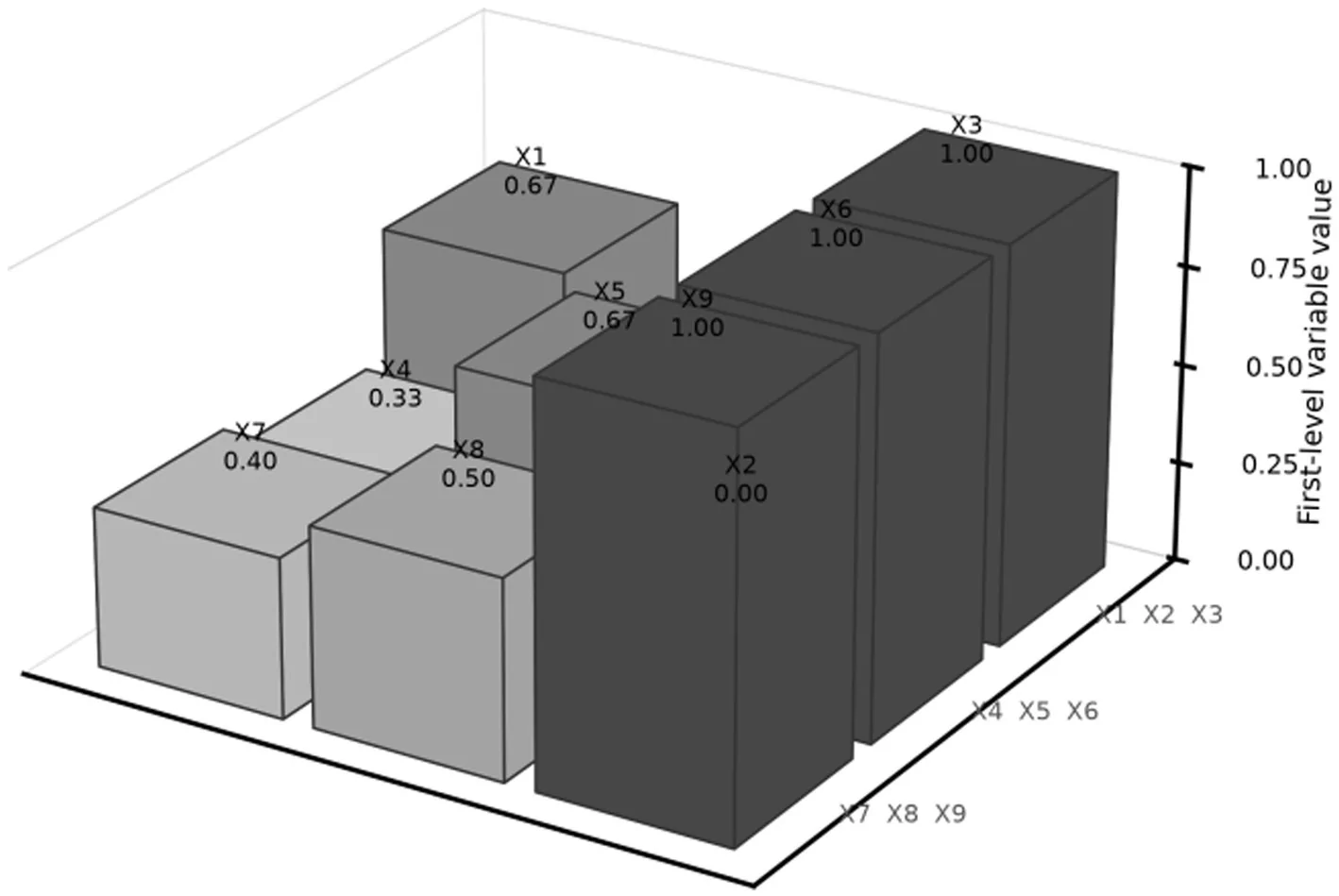
Radar charts of the nine first-level variable scores for the seven policies analyzed individually, ordered by PMC index. In each panel the solid polygon is the policy and the shaded polygon the mean of the 12 policies, so that the panel shows where the policy lies above or below the sample. Axes run *X1* to *X9* clockwise from the top, with the radius scaled 0 to 1. Values are those reported in Table 4. Across the panels, *X2* (policy timeliness) and *X4* (policy level) are drawn in toward the center in every policy, while *X9* (policy domain) and *X6* (participating actors) reach or approach the outer ring throughout.

The discrimination check qualifies two of these Figures. X4 (policy level) and X9 (policy domain) both have zero variance across the sample (Table 4): every document scores exactly 0.33 on X4 and exactly 1.00 on X9. Neither is a substantive finding about the policies. X4 comprises three indicators — national, provincial, and sub-provincial — and the revised definition (S2.3) treats them as a strict administrative hierarchy in which each document occupies exactly one level. Under a mutually exclusive rule of this kind the variable necessarily contributes 1/3 to every document, whatever the sample: its uniform value is a property of the variable’s structure within the PMC specification, not an observation about these twelve policies. A variable defined in this way cannot discriminate between documents, and the appropriate inference is about the specification rather than about the policy set. X9 comprises two indicators, education and sport, and because every document in this policy area is issued jointly by sports and education authorities, both are necessarily satisfied. Its uniform maximum is a structural property of the document type and of the sampling frame that selects for it, not a strength of the policies: a document that did not span both domains would not be a school coaching-position policy in the first place. It should not be read as evidence that these policies perform well on inter-sectoral coverage. Between them the two variables contribute a fixed 1.33 to every policy’s index — about a fifth of a typical score — while contributing nothing to the comparison between policies. Three further variables (X1, X2 and X3) have standard deviations below 0.13. For X1 and X3 this reflects genuine convergence in how the documents are drafted. For X2 the low mean (0.06) rather than the low spread is the finding: nine of the twelve documents attach no time horizon at all to their own arrangements, and the three that do (P8, P13, P15) specify only a medium-term horizon tied to the 14th Five-Year Plan period. The coding manual (S2.2) excludes commencement and validity clauses, tenure and probation periods attached to individuals, and standing annual obligations, none of which states when the document’s own measures are to be completed; the original coding had counted such clauses, which is why every policy previously scored a uniform 0.50 on X2.

The sensitivity analysis (Table 5) tests what follows from this. Removing X4, removing X9, or removing both leaves the ranking of the 12 policies completely unchanged (Spearman ρ = 1.0000 in all three specifications), although the grade distribution shifts because the scale is rescaled against a smaller maximum. Removing X2 as well alters the ranking only marginally (ρ = 0.9926). Two conclusions follow. The relative assessment — which is what the analysis uses to identify design strengths and weaknesses — is determined by the discriminating variables and is robust to the removal of the constant ones. The grade assignment, by contrast, is not robust: on an unchanged ranking the number of policies rated excellent swings from none (X9 removed) to eight (X2, X4 and X9 removed) purely as a function of which constant components are dropped and how the scale is rescaled. That is a further reason for basing interpretation on the variable-level profile rather than on the aggregate score or the grade band.

### Micro-level analysis

To display the configuration of variable content within individual policies, the first-level variable scores of all 12 policies are presented as a heat map (Figure 5), and PMC surface diagrams are given for five cases (Figures 6–10): the national document P1, which is also the lowest-scoring document in the sample; P3 (Fujian), a mid-ranked provincial document; P8 (Zhejiang), the second-highest scoring document; P15 (Inner Mongolia), the highest-scoring document and the autonomous-region case; and P18 (Shanghai), a municipality case. The heat map is the primary comparative display, since it shows all 108 variable values simultaneously and permits direct comparison across policies; the surface diagrams illustrate the concavity representation characteristic of the PMC method. Seven policies are discussed individually below, grouped by administrative level; radar charts are given for all seven in Figure 4, and five are additionally illustrated with PMC surfaces, the number being limited so that the surfaces remain legible at print size.

**Figure 5:**
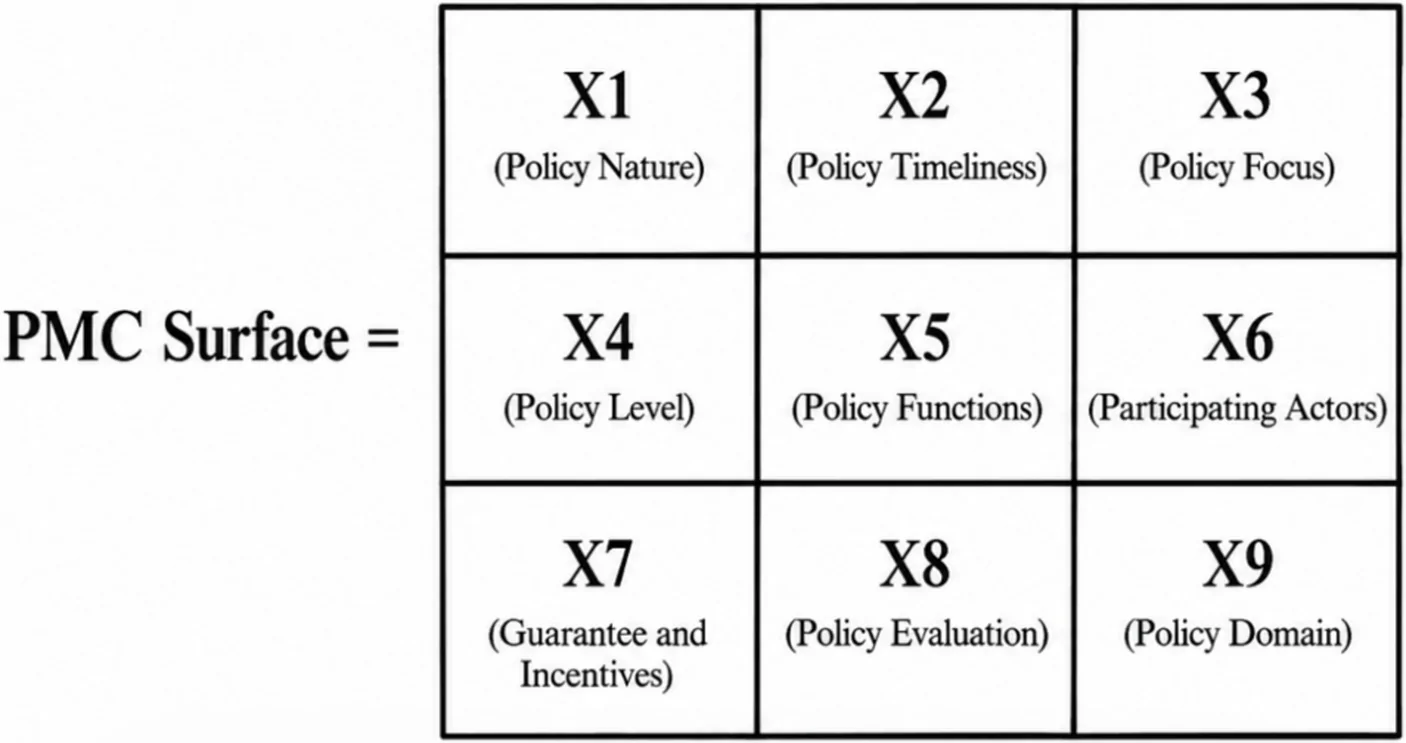
Heat map of first-level variable scores across the 12 policies. Rows are policies in descending order of PMC index; columns are the nine computed first-level variables, with the PMC index reported to the right of the map. Darker shading indicates a higher variable score. The two uniform columns identify the variables with no discriminating power — *X4* is 0.33 for every policy and *X9* is 1.00 for every policy. The near-empty *X2* column shows the common weakness on policy timeliness, where only *P8*, *P13* and *P15* score above zero. What separates the policies from one another is the variation in guarantee and incentives (*X7*), policy evaluation (*X8*) and policy functions (*X5*).

**Figure 6:**
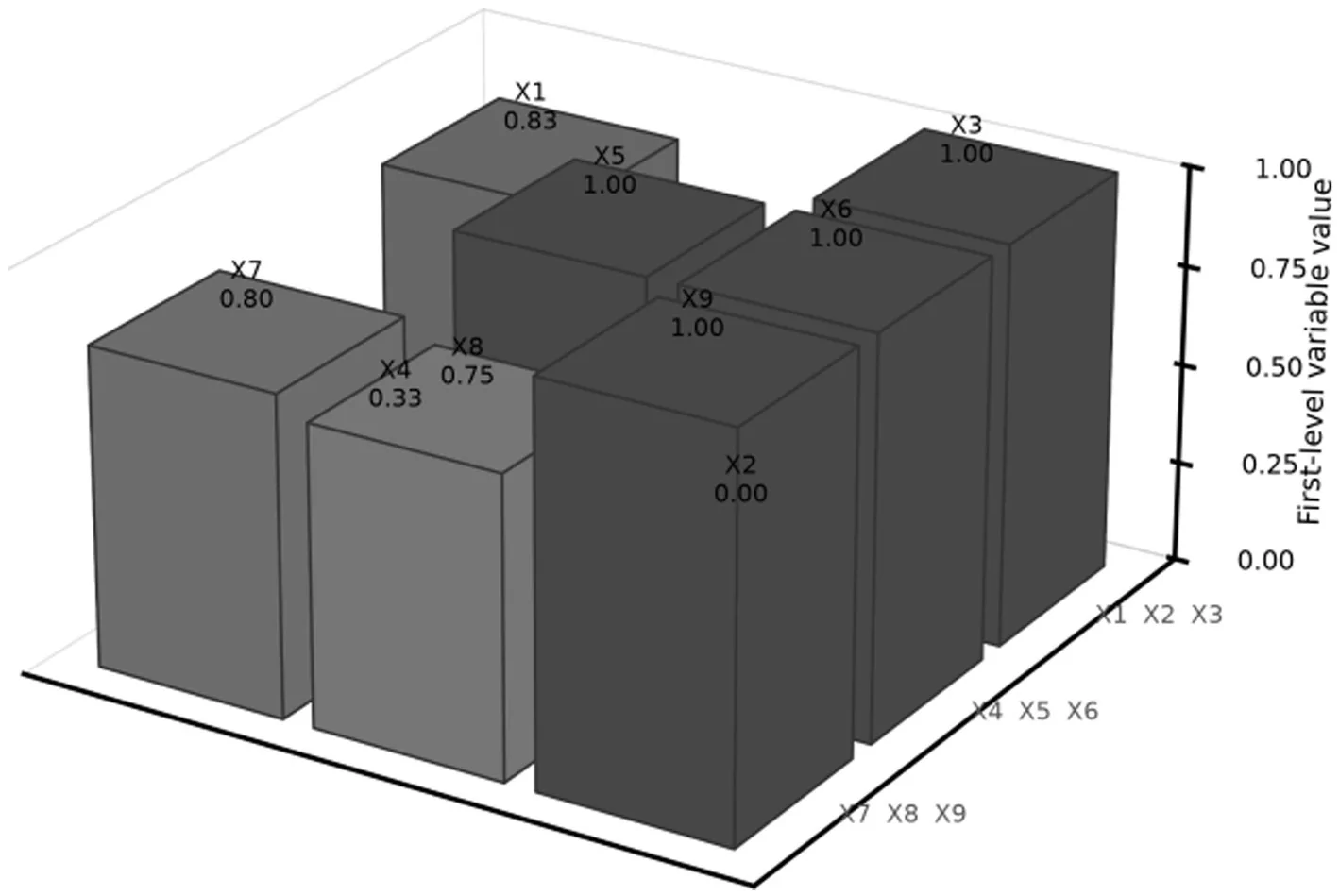
PMC surface diagram of P1, the National Four-Ministry policy and the lowest-scoring document in the sample (PMC 5.57, concavity 3.43, rank 12). Variables are arranged as in Figure 3 and surface height is proportional to the variable value. Depressions mark variables scoring below the sample mean: Policy timeliness (*X2*, 0.00), guarantee and incentives (*X7*, 0.40) and policy evaluation (*X8*, 0.50), together with policy functions (*X5*, 0.67) and policy nature (*X1*, 0.67). *X7* is the joint lowest value on that variable in the sample, which is notable given that the provincial documents implement this one.

**Figure 7:**
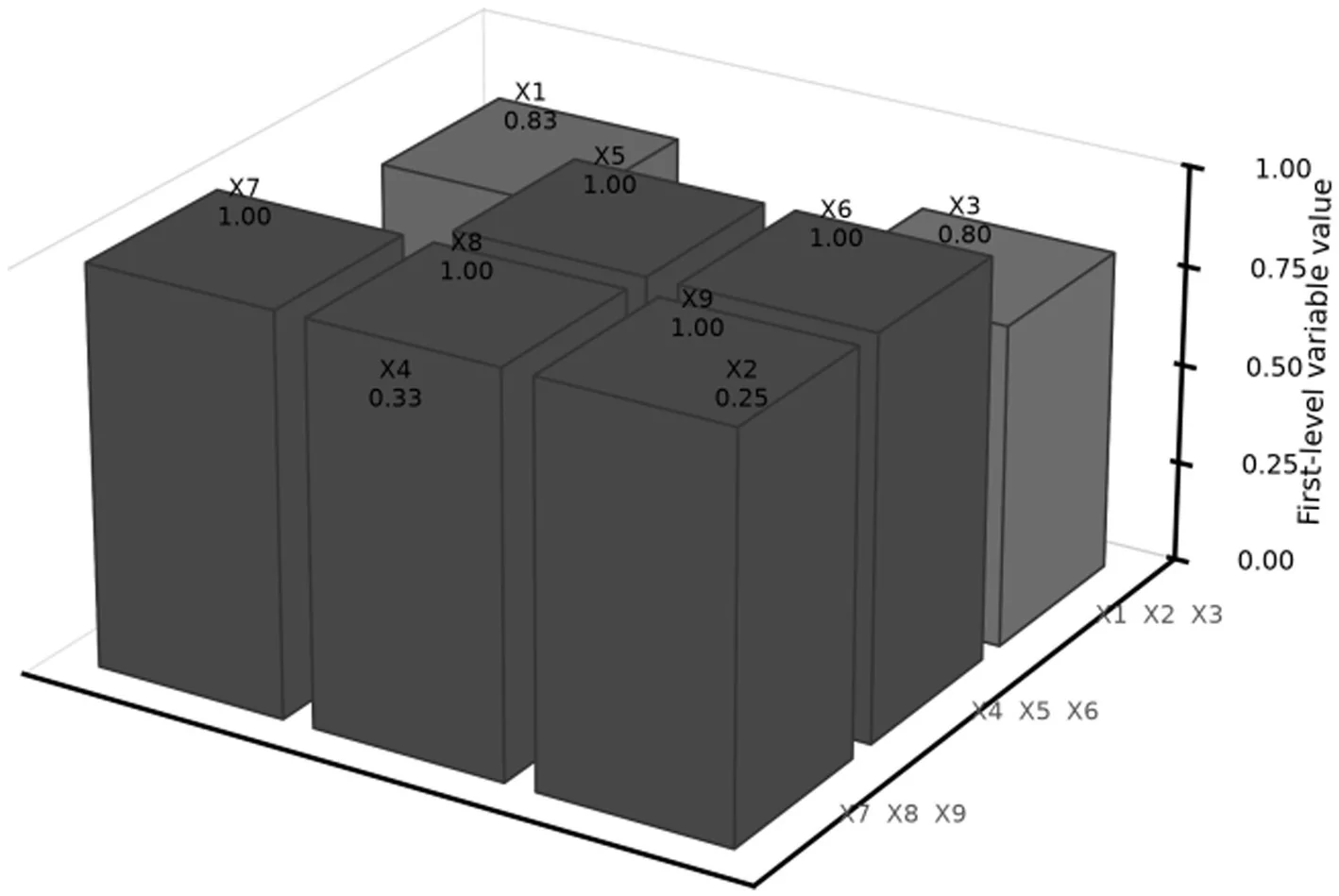
PMC surface diagram of P3, Fujian (PMC 6.13, concavity 2.87, rank 7). Arrangement as in Figure 3. Policy focus (*X3*), participating actors (*X6*), and policy domain (*X9*) reach 1.00, while depressions appear on policy timeliness (*X2*, 0.00), policy evaluation (*X8*, 0.50), and policy functions (*X5*, 0.67).

**Figure 8:**
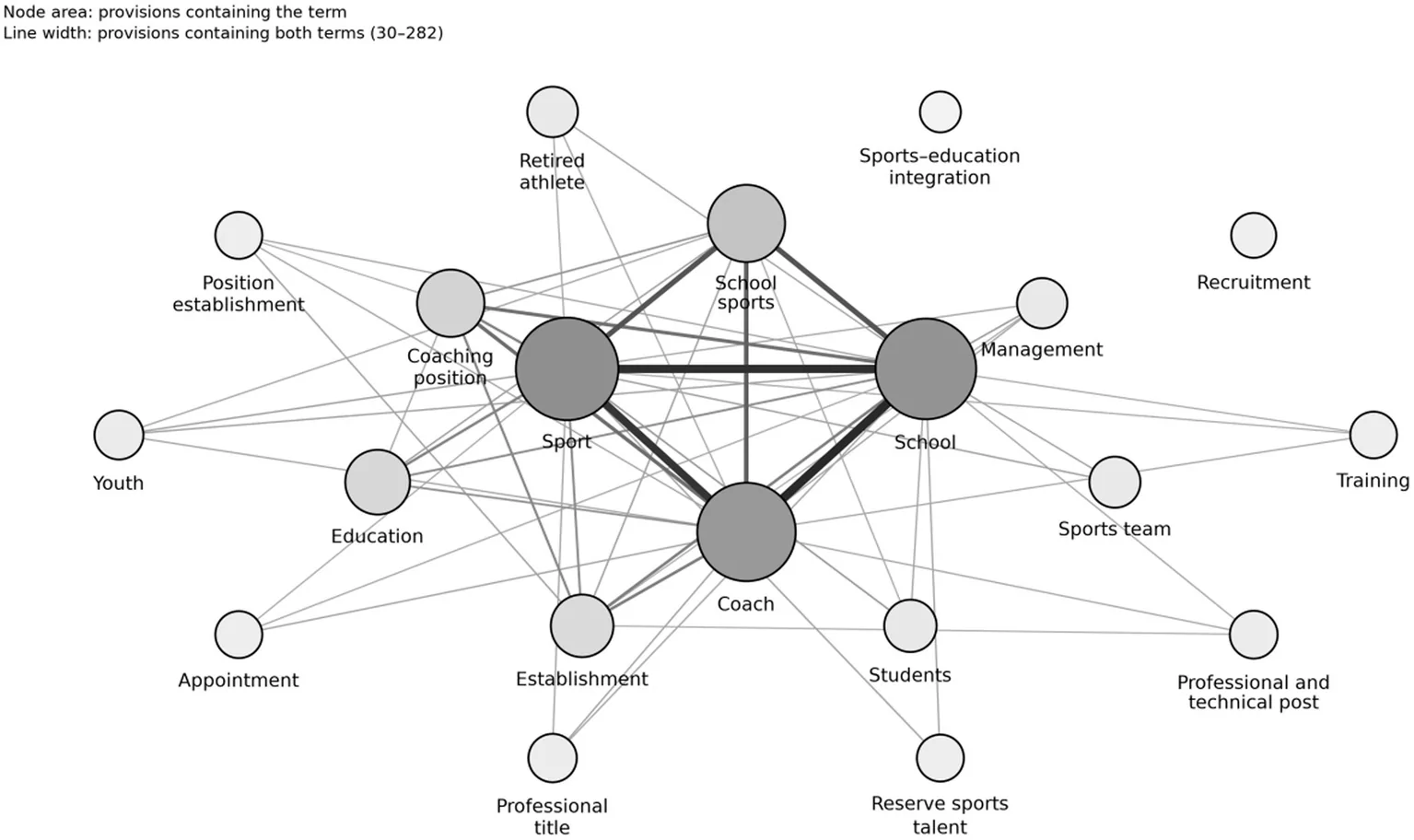
PMC surface diagram of P8, Zhejiang (PMC 7.02, concavity 1.98, rank 2), one of the two documents in the excellent band. Arrangement as in Figure 3. Five variables reach 1.00 — Policy functions (*X5*), participating actors (*X6*), guarantee and incentives (*X7*), policy evaluation (*X8*) and policy domain (*X9*) — and policy focus (*X3*, 0.60) is the only variable below the sample mean. Policy timeliness stands at 0.25, one of only three non-zero values on that variable.

**Figure 9:**
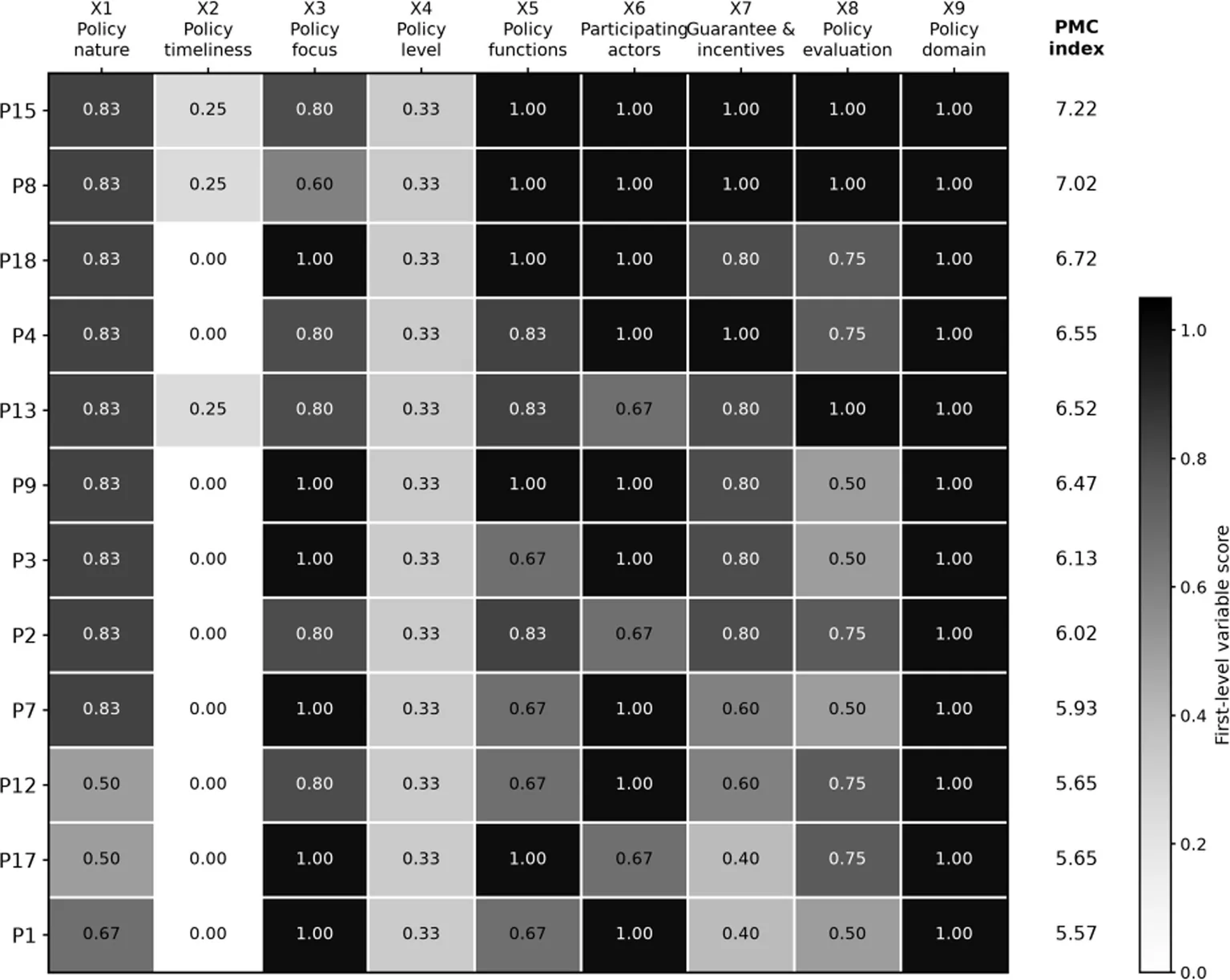
PMC surface diagram of P15, Inner Mongolia Autonomous Region, the highest-scoring document (PMC 7.22, concavity 1.78, rank 1). Arrangement as in Figure 3. The same five variables as in P8 reach 1.00, policy timeliness is again 0.25, and only policy focus (*X3*, 0.80) falls below the sample mean.

**Figure 10:**
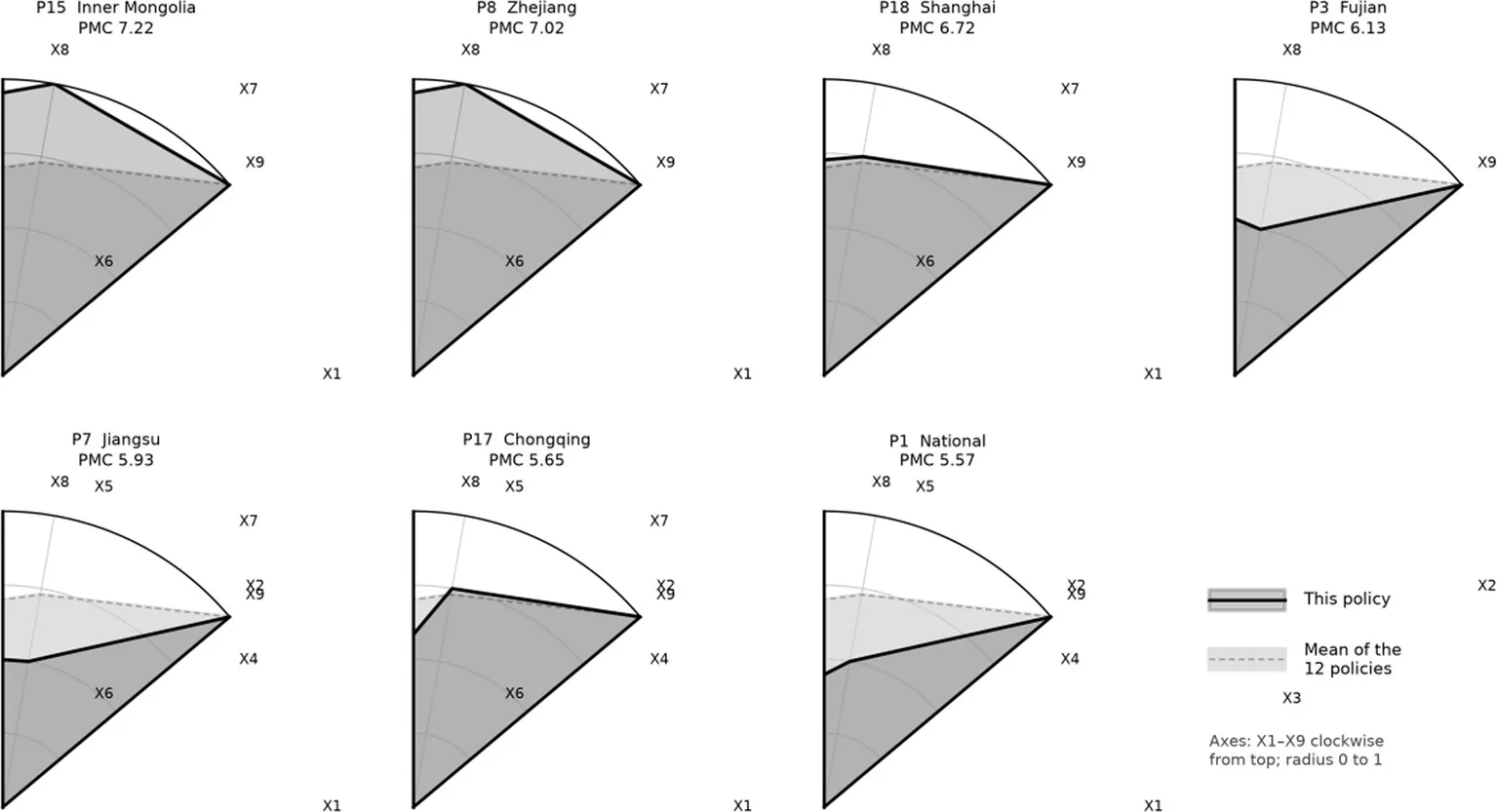
PMC surface diagram of P18, Shanghai (PMC 6.72, concavity 2.28, rank 3). Arrangement as in Figure 3. Policy focus (X3), policy functions (X5), participating actors (X6) and policy domain (X9) reach 1.00; the single depression below the sample mean is policy timeliness (X2, 0.00); policy evaluation (X8, 0.75) and guarantee and incentives (X7, 0.80) sit just above it.

### National-level policy analysis

National-level policies, as foundational documents, provide guidance for local policymaking. They typically proceed from the overall national strategy for education and sport and establish the basic directions, principles, and minimum standards.

Policy P1 obtained a PMC index of 5.57 with a concavity index of 3.43, ranking twelfth of twelve and placing it in the good band. It is a national implementation policy jointly issued by four ministries and commissions to advance the integration of sports and education through the establishment of sports coach positions in primary and secondary schools. Five first-level variables score below the sample mean: policy timeliness (X2, 0.00), guarantee and incentives (X7, 0.40), policy evaluation (X8, 0.50), policy functions (X5, 0.67) and policy nature (X1, 0.67); policy focus (X3), participating actors (X6) and policy domain (X9) all reach 1.00. That the document the provincial policies implement scores lowest in the sample calls for care in interpretation. The score reflects the document’s framework-setting function and its correspondingly low level of operational detail, not inferior drafting or implementation quality: a national framework document is not expected to specify what local documents are to determine. What is informative is which dimensions the gap falls on — a stated horizon, a stated objective, a staged scheme and specified supervision — since these are precisely the ones the provincial documents cannot supply from above. The comparison of a framework document with the implementing documents beneath it also illustrates a general limitation of a single aggregate index: documents performing different functions in a policy hierarchy are not commensurable on one scale, and the national document’s rank should be read with that in mind. Overall, the document exhibits the guiding character typical of national-level policy: it establishes a framework within which provincial and municipal authorities are to develop their own arrangements, and its provisions are correspondingly general. Its design weaknesses lie in the limited specification of forward planning, the incompleteness of provisions on continuing professional development, and the absence of defined supervision and inspection procedures, while incentive mechanisms are stated in general terms without operational detail.

Although the policy stipulates that schools establishing full-time coaching posts may create such positions within their approved staffing quotas, in practice most schools are allowed to establish only one or two positions. For large schools or those offering a wide variety of sports programs, this number may be insufficient to meet actual teaching and training needs. By contrast, schools with weaker sports foundations or lower levels of commitment to physical education may be less motivated to establish such posts, thereby leading to uneven resource allocation. In addition, the policy does not sufficiently account for regional disparities in economic development and educational resources. As a result, economically less-developed areas may face greater difficulties in implementation, such as staffing shortages and inadequate financial support, which may weaken both policy implementation and policy diffusion. The document also allows schools to establish part-time coaching posts, yet the management complexity associated with such arrangements deserves greater attention. Part-time coaches may come from different professional institutions or social organizations, and their working hours, level of commitment, and degree of engagement with school sports may vary considerably. Ensuring that they can effectively fulfill their responsibilities and comply with school teaching and administrative requirements remains an issue that needs to be addressed. Moreover, beyond initial induction training, the policy does not clearly provide sufficient support or institutional guarantees for coaches’ continuing professional development throughout their careers. These are features of the policy text rather than observed outcomes; whether they translate into implementation difficulties is a question that requires implementation data. As a matter of policy design, however, the document would be strengthened by more fully specified guarantee and incentive provisions and by defined arrangements for supervising implementation across provinces and municipalities.

### Municipality-level policy analysis

Municipalities directly under central government administer a smaller and more uniformly urban territory than provinces, and their documents address a single tier of district-level authorities rather than a range of prefecture-level ones. Whether this is reflected in policy design is an empirical question, and the two documents in the sample (P17, P18) answer it differently: they rank third and joint tenth of twelve, so administrative type does not by itself account for design completeness. Both are examined below.

#### Analysis of policy P18

Policy P18, Implementation Opinions on the Management of Professional and Technical Posts for School Coaches in Shanghai, obtained a PMC index of 6.72 with a concavity index of 2.28, ranking third. Policy focus (X3), policy functions (X5), participating actors (X6) and policy domain (X9) all reach 1.00, and the only variable below the sample mean is policy timeliness (X2, 0.00). The document’s distinguishing design features concern resource coordination: it provides for a shared employment mechanism for coaches within the “through-train” school system for sports talent development, coordinates training tasks across educational stages, and provides for the sharing of coaching resources between schools. It also broadens recruitment channels by providing for cooperation with social organizations and for the recruitment of outstanding retired elite athletes, and it envisages a regular mechanism under which front-line coaches work at the grassroots level to identify and select reserve athletes. District-level implementation practice in Shanghai — including annual recruitment targets in Yangpu District and the engagement of external coaches in specialist disciplines by individual schools in Jing’an District — is consistent with these provisions, although such practices lie outside the policy text and are reported here as context rather than as coded evidence. The document’s principal design weakness is that it sets no completion horizon for the arrangements it introduces: it scores 0.00 on policy timeliness, as do eight other documents in the sample. Its provisions state what is to be done and by whom, but not by when.

#### Analysis of policy P17

Policy P17, Implementation Plan of Chongqing Municipality for Establishing Coaching Positions in Primary, Secondary, and Higher Education Institutions, obtained a PMC index of 5.65 with a concavity index of 3.35, tied with P12 at rank 10. Four variables score below the sample mean: policy timeliness (X2, 0.00), guarantee and incentives (X7, 0.40), policy nature (X1, 0.50) and participating actors (X6, 0.67); policy focus (X3), policy functions (X5) and policy domain (X9) reach 1.00. The document is oriented specifically toward the transition of retired athletes into school coaching work, providing for special recruitment events, transition training programs and training bases, and for adjustment of the structural proportion of relevant posts. Chongqing has organized training programs for retired athletes in each year since 2023, including joint provision with Sichuan; as with P18, this implementation activity is reported as context and did not enter the coding. In design terms, the document’s principal weakness is the absence of a supervision mechanism spanning the full sequence of post establishment, recruitment, training and assessment. Without specified procedures at these stages, the document does not establish how compliance with its own requirements is to be verified — a design gap, not an observed failure.

### Autonomous region–level policy analysis

One autonomous-region document remains in the sample after screening. Because comparison between autonomous regions is therefore not possible, the following is presented as a single-case analysis rather than as a characterization of autonomous-region policy in general.

Policy P15 of the Inner Mongolia Autonomous Region obtained a PMC index of 7.22 with a concavity index of 1.78, the highest score in the sample and one of the two documents in the excellent band. Five variables reach 1.00 — policy functions (X5), participating actors (X6), guarantee and incentives (X7), policy evaluation (X8) and policy domain (X9) — and policy focus (X3, 0.80) is the only variable below the sample mean. It is also one of only three documents scoring above zero on policy timeliness (X2, 0.25), by undertaking that post establishment and staffing are to be in place over the course of the 14th Five-Year Plan period. Its ranking rests on the completeness of its guarantee and evaluation provisions rather than on breadth of coverage. Given the region’s territorial extent and the internal disparities in economic development and sports resource distribution that its own text acknowledges, the residual gap is one of differentiation rather than of omission: the guarantee provisions are specified, but they apply uniformly and do not distinguish between banners and counties whose fiscal and staffing capacity differs substantially, so the same obligations fall on units least able to meet them.

### Provincial-level policy analysis

Provincial-level policies are typically formulated considering local conditions, including the distribution of educational resources, the foundations of sports development, and the availability of talent reserves. However, differences across provinces in economic development, educational capacity, and sports traditions lead to variation in policy priorities. Three provincial policies are examined here: P8 (Zhejiang), the highest-scoring provincial document and one of the two in the excellent band, P3 (Fujian), which sits mid-range, and P7 (Jiangsu), the lowest-scoring provincial document. Together they span the range of provincial designs while holding the administrative level constant.

#### Analysis of policy P8

Policy P8, Implementation Opinions on the Management of Professional and Technical Posts for Sports Coaches in Primary, Secondary, and Higher Education Institutions in Zhejiang Province, obtained a PMC index of 7.02 with a concavity index of 1.98, ranking second and placing it in the excellent band. It attains maximum values on policy functions (X5), participating actors (X6), guarantee and incentives (X7), policy evaluation (X8) and policy domain (X9) — the strongest profile in the sample on the guarantee and evaluation dimensions, shared only with P15 (Inner Mongolia). Only three documents reach 1.00 on X7 and only three on X8, and P8 and P15 are the only ones to do both. It is also one of the three documents scoring above zero on policy timeliness (X2, 0.25), undertaking in its organizational clause that post establishment and staffing are to be in place over the 14th Five-Year Plan period. The single value below the sample mean is policy focus (X3, 0.60), the lowest on that variable in the sample: the document addresses post setting, qualification and appointment but says least about the substantive scope of coaching work itself.

The Zhejiang document specifies its staffing arrangements in more concrete terms than most others in the sample, and subordinate documents within the province set out arrangements of the same kind. For example, in some parts of Hangzhou, public schools at the compulsory education stage with more than 600 students have established at least one full-time or part-time sports coaching post in accordance with the policy, thereby providing both institutional justification and staffing support for coach recruitment. In cities such as Quzhou, local authorities may recruit or introduce two sports coaches from among retired athletes within the province and incorporate them into the public staffing system. In addition, Zhejiang has broadened the channels for sports talent recruitment by opening school coaching posts to sports talent trained through social organizations, while encouraging outstanding retired athletes and other sports professionals from across China and even abroad to work in Zhejiang. Read as policy design, the document’s strength lies in specifying its provisions at an operational level: it states the conditions under which posts are to be established, identifies the channels through which coaches may be recruited, and assigns responsibility for each. The implementation practices described above are drawn from municipal documents and reports and are given as context; they were not used as coding evidence for P8.

#### Analysis of policy P7

Policy P7, Implementation Opinions of Jiangsu Province on Establishing Coaching Positions in Schools, obtained a PMC index of 5.93 with a concavity index of 3.07, ranking ninth. Policy focus (X3, 1.00) and participating actors (X6, 1.00) both reach the maximum, and policy nature (X1, 0.83) is above the sample mean. The shortfall is concentrated in four variables scoring below the mean: policy timeliness (X2, 0.00), policy functions (X5, 0.67), guarantee and incentives (X7, 0.60) and policy evaluation (X8, 0.50). The document sets no horizon for its own arrangements, states no objective with a verifiable time point, quantity or coverage, and specifies no staged implementation plan; its five-year references — a validity period running to 8 September 2028 and a requirement that newly appointed coaches remain in post for at least five years — concern the document’s own currency and the individual coach’s tenure rather than the schedule of the measures it introduces.

Subordinate documents and programs in Jiangsu provide for social participation in school sport alongside the provincial policy. For example, the “Champion Coaches Enter Campus” initiative jointly led by the Changzhou Sports Bureau and Education Bureau integrates resources from schools, teachers, and sports-school coaches to meet students’ diverse sporting needs and to promote a new pattern of sports–education integration in Changzhou. The Jiangsu policy states that coaching performance during service may be recognized as valid evidence for professional title evaluation. However, in practice, the quantification and assessment of these indicators remain difficult, and the lack of clear, specific, and operable standards may lead to subjectivity and uncertainty in evaluation, thereby weakening both accurate appraisal and effective incentives. Moreover, the document sets uniform requirements for all prefecture-level cities in the province without differentiating by local fiscal or staffing capacity, although its own text elsewhere acknowledges variation in conditions across the province. Where capacity is uneven, a policy that does not differentiate its requirements leaves the areas with least capacity subject to the same obligations as those with most. This is a feature of the policy design as drafted; whether it produces differential implementation is an empirical question the present study cannot answer, since testing it would require regional socioeconomic and implementation data that this study does not use.

#### Analysis of policy P3

Policy P3, Implementation Measures of Fujian Province for Establishing Coaching Positions in Schools, obtained a PMC index of 6.13 with a concavity index of 2.87, ranking seventh of twelve. Policy focus (X3, 1.00) and participating actors (X6, 1.00) reach the maximum and policy nature (X1, 0.83) is above the sample mean. Three variables fall below it: policy functions (X5, 0.67), policy evaluation (X8, 0.50) and policy timeliness (X2, 0.00). The X8 value reflects the absence of a stated objective, of an explicit justification for the measures and of a staged implementation scheme; guarantee and incentives (X7, 0.80) is at the upper end of the sample but still short of a specified supervision mechanism with defined consequences.

The document provides provincial-level authorization for establishing coaching posts but specifies the supporting arrangements less fully than the higher-scoring documents. It does not set out a regulatory framework for coach recruitment, training and assessment, nor procedures for supervising teaching quality and job performance; and it does not differentiate its requirements according to local fiscal capacity, although capacity varies substantially within the province. Furthermore, although the policy proposes a “two-way introduction” mechanism between physical education teachers and school sports coaches, actual operation may be constrained by the limited number of available posts, thereby restricting mobility between the two types of positions.

## Discussion

### Principal findings

Across the 12 policy documents examined, policy design is structurally complete on most dimensions and consistently incomplete on three. Every document delimits its domain and focus and names the actors responsible: policy domain (X9) is uniformly at the maximum, participating actors averages 0.92 and policy focus 0.88. Ten of the twelve documents fall in the good band and two in the excellent band. Design weaknesses are concentrated in policy timeliness (X2, mean 0.06), policy evaluation (X8, 0.73) and guarantee and incentives (X7, 0.75). The pattern is consistent rather than idiosyncratic: nine of the twelve set no horizon at all for their own arrangements and the remaining three set only a medium-term one; four score 0.50 on policy evaluation, lacking a stated objective and a staged scheme alike; and on guarantee and incentives the national four-ministry document itself scores lowest at 0.40. The sensitivity analysis establishes that this profile does not depend on the specification of the framework, since the ranking is unchanged by removal of the two non-discriminating variables (ρ = 1.0000) and nearly unchanged by removal of X2 as well (ρ = 0.9926).

### Why these weaknesses arise

The three weak dimensions are not independent defects of individual documents; each is traceable to a feature of how this policy area is institutionally organized, which is why the pattern recurs across documents issued by different authorities.

The uniform absence of medium- and long-term planning follows from the position these documents occupy in the policy hierarchy. Eleven of the twelve are local implementation documents that operationalize central directives, and they inherit their timetable from those directives rather than setting one. Their function is to specify how a national requirement is to be carried out locally, and the planning horizon is treated as given at the level above. This explains why the weakness is uniform, and it also indicates where the remedy lies: at the national level rather than the provincial one. The three documents that do state a horizon (P8, P13, P15) illustrate the point, since each does so by referring to the 14th Five-Year Plan period — that is, by adopting a nationally set planning frame rather than constructing one of their own. The national document P1, for its part, states no horizon at all, so there is no timetable at the top of the hierarchy for the others to inherit.

The weakness on policy evaluation (X8) has the same origin. Its four components are a stated objective, a cited basis, a staged or structured plan, and operationally specific content. All twelve documents cite their basis and all provide specific content; what is missing is the first and third components, which are precisely the forward-looking ones. Only three state an objective containing a verifiable time point, coverage or quantity, and four set out no staged or structurally differentiated plan at all — neither a sequence of phases nor different obligations for different categories of school. A document that inherits its horizon from above has no occasion to formulate either, so the low X8 values are the same institutional fact as the low X2 values seen from another angle rather than an independent defect. Participating actors (X6) shows the complementary pattern: because the instrument works through the public-institution staffing system, the operative decisions on quotas and title criteria belong to government departments, and all twelve documents assign roles to those departments and to schools. Nine also assign tasks to social organizations, clubs or professional agencies; the three that do not (P2, P13, P17) mention clubs only within the coach’s own job duties.

The weak specification of supervision follows from the division of labor within the Chinese administrative system, where supervision and performance assessment are ordinarily assigned to separate assessment instruments issued by the departments responsible for personnel management. Position-establishment documents create posts; they do not usually contain the mechanisms by which compliance is audited. This has an implication for evaluation: an assessment of supervision provision confined to position-establishment documents will systematically understate the supervision actually in place, and a full account would require the associated assessment documents to be analyzed alongside them.

Policy nature (X1, mean 0.76) is not among the weak dimensions but shows a related gap: the documents are predominantly regulatory, descriptive and guiding, while forecasting content is largely absent. This is consistent with the absence of a planning horizon — a document that does not state when its own arrangements are to be completed has little occasion to forecast.

A methodological point follows from the comparison between P1 and the documents that implement it. A framework document and an implementing document are not designed to do the same thing: the first establishes principles and delegates specification, the second supplies it. An index that rewards operational detail therefore scores them on a dimension along which they are not commensurable, and P1’s position at the foot of the ranking is in part a consequence of that rather than a finding about the document. Applications of the PMC model to samples spanning several tiers of a policy hierarchy would do well either to compare within tiers or to report the tier alongside the score, as we do here.

### International comparison

The design weaknesses identified are not all equally specific to the Chinese setting. The absence of long-horizon planning and the weak specification of supervision are recurrent findings in policy analyses of school-based physical activity provision across national settings, where implementation frequently outruns the policy frameworks intended to sustain it (6, 17). Establishing which actors participate in provision, by contrast, is more institutionally specific. Where school coaching is organized through contracting or certification markets, external providers are necessarily parties to the arrangement, and the policy question becomes one of quality assurance and access rather than of departmental coordination (7). The Chinese instrument reverses this: coordination among government departments is comparatively well specified in these documents, whereas the terms on which non-governmental actors participate are not. The case therefore extends policy-design research rather than merely localizing it, by showing what a coaching policy looks like when coaching posts are embedded in a public-sector staffing and professional-title system: the design problems that become central are establishment quotas, title criteria and inter-departmental coordination, and the ones that recede are certification and contracting. A further question that arises in both settings is the boundary between the physical education teacher’s role and the coach’s (3). The documents examined here assign coaches primarily to training and competition work, but the division of responsibilities where the two roles operate in the same school is not specified in most of them.

What distinguishes the higher-scoring documents is not the breadth of the dimensions they cover, since almost all cover the same ones, but whether they specify the forward-looking components. The two documents in the excellent band (P15, P8) are the two that combine a stated horizon for their own arrangements with a complete score on policy evaluation (X8 = 1.00) and on guarantee and incentives (X7 = 1.00); the four lowest-ranked (P7, P12, P17, P1) all score 0.50 or 0.75 on policy evaluation and 0.60 or below on guarantee and incentives. It should be noted that these are properties of the documents, and that a well-specified document is a precondition for coordinated implementation rather than a guarantee of it.

On policy timeliness, nine of the 12 documents attach no horizon whatever to their own arrangements, and the three that do (P8, P13, P15) specify only the medium term, in each case by reference to the 14th Five-Year Plan period. None specifies a short-term completion deadline, a current-year task or a long-term trajectory. The mean of 0.06 is the lowest of the nine first-level variables by a wide margin, and it should be read as a characterization of the policy area as a whole rather than as a means of ranking documents within it: at the time of study, this was a field in which the design of coaching posts was specified in detail but the schedule on which those posts were to be filled was largely left unstated. It bears emphasis that this figure is not the presence of a timetable understated by conservative coding. Under the revised rules (S2.2) commencement and validity clauses, tenure and probation periods attached to individuals, and standing annual obligations do not count, because none of them says when the document’s own measures are to be completed. The original coding counted such clauses, which is what produced the uniform 0.50 reported previously.

### Policy implications

1. Specify the professional-title evaluation criteria for school sports coaches in operational terms. Several documents provide that coaching performance during service may count toward title evaluation, but do not state how it is to be measured. Absent defined and measurable criteria, the provision cannot function as intended as an incentive, since neither coaches nor evaluators can determine in advance what satisfies it. Specifying the criteria — what is counted, over what period, and against what benchmark — is a concrete drafting task, and one that addresses the weakness recorded on guarantee and incentives (X7).
2. Expand cooperation between schools and professional sports institutions to introduce high-quality coaching resources into schools. The development of the school sports coaching workforce requires the joint efforts of the government, schools, and social organizations. Given the current shortage of professional sports education services in schools, cross-sector collaboration should be strengthened in order to provide schools with qualified sports coaches and lay a stronger foundation for the cultivation of students’ sports literacy.
3. State a completion horizon for the arrangements each document introduces, beginning at the national level. Nine of the twelve documents set no horizon at all, and the three that do adopt the 14th Five-Year Plan period rather than a schedule of their own; the national four-ministry document, which the provincial documents implement, states none. The most direct remedy is therefore for the national document to set out a staged trajectory — by when posts are to be established, in which categories of school, and to what coverage — against which local arrangements can be planned. Provincial documents would then need to convert that trajectory into their own multi-year arrangements adapted to local fiscal and staffing capacity, in place of the open-ended formulations that currently prevail across the policy set.

## Limitations

### Indicator selection

Although the indicator framework was refined through two Delphi rounds with statistical consensus checks, the candidate pool was derived from prior PMC applications and from the authors’ reading of the corpus, and therefore embodies analytic choices that other researchers might make differently. Delphi consensus establishes agreement among the panel consulted; it does not establish that the indicator set is uniquely correct.

Coding judgment and the absence of independent double coding. Binary coding requires a decision as to whether policy content addresses an indicator. The coding manual, the requirement of a recorded text locator for every positive code, the convention of coding ambiguous content 0, and the adjudication of inconsistencies were adopted to constrain that judgment, but not to eliminate it. More importantly, the coding was performed by a single coder, and the reliability reported here is test–retest consistency rather than inter-coder reliability. This is a genuine limitation and not a formality: a test–retest design detects unstable application of a decision rule, but a rule that the coder has consistently misread, or a construct such as policy focus that the coder interprets in a defensible but idiosyncratic way, will be reproduced identically in both passes, so stable error cannot be distinguished from stable correctness. Only an independent second reader can make that distinction. We mitigate the constraint by publishing the complete item-level coding sheet with textual warrants (Supplementary material S4), which allows readers to examine individual judgments and to re-code the sample themselves, but we do not claim that this substitutes for independent double coding. Replication of the coding by researchers outside this team would be a worthwhile check on the findings reported here.

### Properties of the PMC index

Three limitations of the method bear on interpretation. First, the index weights all first-level variables equally by construction, which implies that policy timeliness and policy domain contribute as much to the aggregate as guarantee and incentives — an assumption of convenience rather than one derived from theory. Second, because each first-level variable is the unweighted mean of its second-level indicators, variables composed of few indicators are more volatile than those composed of many: X9, with two indicators, moves in steps of 0.50, whereas X1, with six, moves in steps of about 0.17. Third, and most consequentially, the index registers the presence of policy content rather than its stringency, quality or feasibility. A document referring to supervision in general terms is scored identically to one specifying an inspection procedure with defined consequences. High scores therefore indicate completeness of policy design, and nothing more than that.

### Equal weighting and binary coding

The PMC index assigns equal weight to every first-level variable, so policy timeliness contributes as much to the aggregate as guarantee and incentives, although the two are unlikely to matter equally for how a policy works. The weighting is a convention of the method, not a finding of this study, and the sensitivity analysis tests the ranking against it rather than defending it. Binary coding compounds the point: an indicator records whether a document addresses a dimension, not how far it goes, so a document mentioning supervision in general terms and one specifying a responsible agency, an inspection frequency and a consequence for non-compliance receive the same code. Differences in stringency of that kind are invisible to the index, and the grade bands rest on thresholds adopted from prior applications rather than on empirically derived cut points in these data.

### Non-discriminating indicators

Two variables, policy level (X4) and policy domain (X9), had zero variance in this sample and contributed a fixed 1.33 to every score, as reported in the Results. Their inclusion raises the mean without affecting the ranking. X9 might discriminate in a wider sample — one including sub-provincial documents, which are not necessarily issued jointly by sports and education authorities. X4 would not: its categories are mutually exclusive by construction, so it contributes 1/3 to every document in any sample coded under this rule, and it is better regarded as a classification recorded alongside the index than as a component of it. Policy timeliness (X2) is a different case: it retains two distinct values and so is not constant, but with nine of twelve documents at zero it does little to separate them. That near-uniformity is a finding about the corpus rather than a defect of the instrument, and the sensitivity analysis (Table 5) confirms the ranking barely moves when X2 is removed. It does mean, however, that a policy set in which horizons were more commonly stated would be needed before X2’s discriminating capacity could be assessed.

### No implementation or outcome evidence

The study analyses policy texts. It cannot establish whether posts were created, whether coaches were recruited or retained, whether implementation matched policy intent, or whether any change followed in students’ physical activity or health. Claims of that kind require implementation data and are not made here.

### Sample coverage

Six documents were excluded because their full official texts could not be obtained or because they failed the inclusion criteria on other grounds. The sample therefore covers publicly obtainable formal policies rather than the complete policy population, and it is weighted toward national and provincial documents because sub-provincial implementation rules are unevenly published. One consequence is that only one autonomous-region document remains, so the study cannot support comparison between autonomous regions or characterize autonomous-region policy as a class.

### Institutional specificity

The indicator framework encodes features of the Chinese administrative system: establishment-based staffing, the professional-title system, and the vertical relationship between national, provincial and sub-provincial documents. The findings describe policy design within that setting and should not be read as directly transferable to systems in which school coaching is organized through other instruments.

### Cross-sectional design

The sample covers documents issued between 2022 and 2024, an early phase in the development of this policy area, and the 2026 verification round confirmed sources rather than extending that window. Provinces that had not issued a document by the end of 2024 are therefore absent, and any document issued or revised since falls outside the sample. Policy design is likely to change as implementation experience accumulates, so the findings characterize the policy set as it stood in that period rather than the current policy landscape or a settled framework.

## Conclusion

This study evaluated the design of 12 formally promulgated policy documents on the establishment of school sports coach positions in China, using a PMC index framework of nine computed first-level variables and 38 s-level indicators developed through a two-round Delphi consultation. These policies are comparatively complete across most of the design dimensions measured: every document delimits its domain and focus, cites its basis, provides operationally specific content and names the actors responsible. Design weaknesses are concentrated and consistent across the sample, in policy timeliness (mean 0.06), policy evaluation (0.73) and guarantee and incentives (0.75). The mean aggregate index is 6.29, with ten documents in the good band and two in the excellent band; the grade bands carry little interpretable information, since ten of the twelve fall in one band and the sensitivity analysis shows the number graded excellent ranging from 0 to 8 across specifications on an unchanged ranking. Two variables were found to have no discriminating power in this sample — policy level, because the revised coding assigns each document exactly one administrative level, and policy domain, because every document is issued jointly by sports and education authorities — and the sensitivity analysis showed that removing them leaves the ranking of the policies unchanged, indicating that the assessment rests on the seven discriminating variables and that the narrow spread of absolute scores is partly an artefact of constant components. Three implications follow for policy design: a completion horizon for the arrangements introduced needs to be stated, at the national level as well as locally, since the provincial documents take their planning frame from the national one and none of the three that state a horizon constructs one of its own; objectives and staged implementation schemes require specification, these being the components of policy evaluation the documents most often omit; and title-evaluation criteria need to be stated in measurable form if they are to function as incentives. Because the analysis is confined to policy texts, it establishes what these policies specify and how coherently, not what they have achieved. Whether coaching posts have been created, filled and sustained, and with what consequences for school sport, requires implementation data — post establishment records, recruitment statistics and outcome measures — and is the necessary next stage of research in this area.

## Data Availability

The original contributions presented in the study are included in the article/supplementary material, further inquiries can be directed to the corresponding authors.

## Footnotes

1
www.gov.cn
