# Supplementary material for: Evaluation of school sports coach position policies in China based on the PMC Index Model
Source: Front Public Health. 2026 Sep 18;14:1880356. doi: 10.3389/fpubh.2026.1880356 (PMC13630741; doi:10.3389/fpubh.2026.1880356)
Supplement: Supplementary file 1 [file Supplementary_file_1.docx]

**Supplementary Material S1. Delphi Expert Consultation Procedure**

This supplement summarises the Delphi procedure used to refine the PMC evaluation framework, including panel composition, response rates, consensus statistics, and the basis for retaining indicators.

# **S1.1 Procedure and expert panel**

1. Draft framework construction. Candidate indicators were developed from policy-text analysis and prior PMC-based studies relevant to school sports coach positions.

2. Expert panel. Fifteen experts were selected to combine academic expertise with practical experience in physical education, coaching, sports management, and school practice.

3. Two-round consultation. Experts rated candidate indicators and provided written comments; wording was revised between rounds where comments showed ambiguity.

4. Finalisation. The final indicator framework was determined after considering rating concentration, dispersion, concordance, and written comments together.

**Table S1. Composition of the expert panel (N = 15)**

| **No.** | **Name** | **Title / Position** | **Research field and relevant achievements** | **Affiliation** |
| --- | --- | --- | --- | --- |
| 1 | Li Chengliang | Professor / Vice-President | Sports event organization and management; Executive Committee member, Chinese Basketball Association | Shenyang Sport University |
| 2 | Cai Yujun | Professor / Dean of Academic Affairs | Physical education teaching and training; International-level gymnastics referee | Shanghai University of Sport |
| 3 | Zhai Feng | Professor / Dean | Integration of sports and education; Head of provincial university athletics team | China University of Mining and Technology |
| 4 | Xue Hao | Professor | Traditional ethnic sports; retired athlete | Nanjing Sport Institute |
| 5 | Fan Bin | Professor / Associate Dean | Football teaching and training; head coach of football team | Huaibei Normal University |
| 6 | Wang Qing | Associate Professor | Theory and methods of athletics training; lecturer, Chinese Athletics Association | Huaibei Normal University |
| 7 | Wang Xunling | Associate Professor | Physical education teaching and evaluation; head of athletics course group | Huaibei Normal University |
| 8 | Ji Chaoxin | Associate Professor | Skating teaching and training; national-level referee | Northeastern University |
| 9 | Gao Huanyu | Associate Professor | Psychology of winter-sport athletes; national-level referee | Shenyang Sport University |
| 10 | Xu Jingzheng | Senior Teacher / Vice-Principal | Leading figure in physical education, Huaibei | Huaibei No. 1 Middle School |
| 11 | Zhang Houxue | Full Senior Teacher | Head of Huaibei municipal PE master-teacher studio; municipal teaching researcher | Huaibei Experimental Senior High School |
| 12 | Wang Hongwei | First-Class Teacher | Basketball teaching and training; retired athlete; championship coach, provincial senior-high boys' basketball | Huaibei Experimental Senior High School |
| 13 | Zhang Hui | First-Class Teacher | Football teaching and training; head coach, municipal youth football team | Huaibei Experimental Senior High School |
| 14 | Sun Jian | First-Class Teacher / Head of PE Group | Badminton teaching and training | Huaibei No. 5 Middle School |
| 15 | Xu Wei | Senior Teacher / Head of PE Group | Physical education teaching and training | Huaibei Meiyuan School |

The panel comprised nine university-based experts and six front-line practitioners.

# **S1.2 Delphi response and consensus**

All 15 experts returned questionnaires in both rounds (100% response rate). Mean ratings increased between rounds, coefficients of variation narrowed, and Kendall's W increased at both indicator levels; all reported W tests were significant at p < 0.001.

**Table S2. Questionnaire response (N = 15)**

| **Consultation round** | **Distributed** | **Returned** | **Response rate** |
| --- | --- | --- | --- |
| Round 1 | 15 | 15 | 100% |
| Round 2 | 15 | 15 | 100% |

**Table S3. Concentration of expert opinion after two Delphi rounds (N = 15)**

| **Indicator level** | **Mean range (Round 1)** | **Mean range (Round 2)** |
| --- | --- | --- |
| First-level indicators | 3.800–4.733 | 4.000–4.933 |
| Second-level indicators | 3.800–4.800 | 4.067–4.933 |

**Table S4. Coordination of expert opinion, Round 1 (N = 15)**

| **Indicator level** | **CV range** | **Kendall's W** | **p** |
| --- | --- | --- | --- |
| First-level indicators | 0.097–0.227 | 0.382 | < 0.001 |
| Second-level indicators | 0.086–0.227 | 0.373 | < 0.001 |

**Table S5. Coordination of expert opinion, Round 2 (N = 15)**

| **Indicator level** | **CV range** | **Kendall's W** | **p** |
| --- | --- | --- | --- |
| First-level indicators | 0.052–0.217 | 0.430 | < 0.001 |
| Second-level indicators | 0.052–0.207 | 0.489 | < 0.001 |

Note on Kendall's W. The surviving SPSS output supports the W values and p-values reported above, but its stored degrees of freedom do not correspond to the final 10 first-level variables and 38 second-level indicators. Therefore, χ² and degrees of freedom are not reported here.

# **S1.3 Expert authority**

Expert authority was summarised by the familiarity coefficient (Cs) and judgment-basis coefficient (Ca). Cs = 0.827 and Ca = 0.940, giving an authority coefficient Cr = 0.883.

**Table S6. Expert familiarity with the subject**

| **Familiarity level** | **Not familiar** | **Slightly familiar** | **Moderately familiar** | **Familiar** | **Very familiar** |
| --- | --- | --- | --- | --- | --- |
| Number of experts | 0 | 0 | 5 | 3 | 7 |
| Assigned weight | 0.2 | 0.4 | 0.6 | 0.8 | 1.0 |
| Familiarity coefficient | C_s = 0.827 |  |  |  |  |

**Table S7. Basis of expert judgment**

| **Basis of judgment** | **Large (n / weight)** | **Medium (n / weight)** | **Small (n / weight)** | **Score** |
| --- | --- | --- | --- | --- |
| Practical experience | 11 / 0.5 | 4 / 0.4 | 0 / 0.3 | 0.473 |
| Theoretical analysis | 10 / 0.3 | 5 / 0.2 | 0 / 0.1 | 0.267 |
| Knowledge of domestic and international peers | 2 / 0.1 | 1 / 0.1 | 12 / 0.1 | 0.100 |
| Intuition | 4 / 0.1 | 2 / 0.1 | 9 / 0.1 | 0.100 |
| Judgement-basis coefficient | C_a = 0.940 |  |  |  |

# **S1.4 Indicator retention and outcome**

No fixed numerical cut-off was pre-specified. Indicator retention was based on the arithmetic mean, coefficient of variation, Kendall's W and its significance, together with experts' written comments. Indicators judged unclear were revised and re-rated rather than automatically removed.

The final framework contains 10 first-level variables. Nine are decomposed into 38 second-level indicators and enter the PMC calculation; X10 (policy disclosure) is retained conceptually but has no second-level indicators and is not computed.
