# Supplementary material for: Evaluation of school sports coach position policies in China based on the PMC Index Model
Source: Front Public Health. 2026 Sep 18;14:1880356. doi: 10.3389/fpubh.2026.1880356 (PMC13630741; doi:10.3389/fpubh.2026.1880356)
Supplement: Supplementary file 2 [file Supplementary_file_2.docx]

**Supplementary Material S2. Coding Manual for Policy Text Evaluation**

This manual specifies the decision rules used to assign binary values (0/1) to the 38 second-level indicators of the PMC framework and summarises the intra-coder test–retest check used in this study.

# **S2.1 General coding rules**

Unit of analysis. The full promulgated policy document, including its title, issuing notice, numbered articles, and annexes forming part of the same document. Separately published interpretations, press releases, Q&A responses, and news reports are not coding evidence.

Assignment rule. Code 1 when the policy contains an explicit substantive provision addressing the indicator; code 0 when the indicator is absent, appears only in a cited higher-level title, or remains genuinely ambiguous.

Evidence rule. Every code of 1 must have an article/section locator and a quoted phrase from the promulgated text.

Borderline rule. Ambiguous content is coded 0 rather than resolved in the direction of a higher score.

Blind re-coding rule. The second coding pass was conducted from the policy texts and this manual without consulting the earlier coding sheet or previously computed scores.

# **S2.2 Indicator-level decision rules**

X1 Policy Nature (6 items). Code what the document actually does; a single provision may support more than one item.

| **Item** | **Code 1 when the text…** | **Code 0 when…** |
| --- | --- | --- |
| X1:1 Forecasting | states expected future conditions, projected demand, or targets tied to a future date (e.g. "by 2030 the number of established posts will reach…") | contains only present-tense requirements |
| X1:2 Regulation | imposes binding requirements, prohibitions, qualification thresholds, or compliance consequences | uses only hortatory language ("encourage", "support") |
| X1:3 Recommendation | offers optional or advisory arrangements that localities *may* adopt | all provisions are mandatory |
| X1:4 Description | describes the existing situation, background conditions, or the problem being addressed | contains no situational description |
| X1:5 Guidance | sets direction, principles, or guiding ideology for subordinate bodies to follow | contains no directional/principled statement |
| X1:6 Others | contains a nature not covered above (e.g. authorising, interpretive, transitional provisions) | none applies |

X2 Policy Timeliness (4 items). Code only a time horizon for implementation of the document's own arrangements. Do not count general commencement/validity clauses, individual tenure or probation periods, or standing recurring obligations as implementation horizons.

| **Item** | **Code 1 when the text…** |
| --- | --- |
| X2:1 Short-term | specifies arrangements within roughly 1–2 years, or names a completion deadline in that range |
| X2:2 Medium-term | specifies arrangements over roughly 3–5 years, including references to the Five-Year Plan period ("十四五期间") |
| X2:3 Long-term | specifies arrangements beyond roughly 5 years, or names a target year more than 5 years ahead |
| X2:4 Within the current year | names a task to be completed within the calendar year of issuance |

Under this rule, only P8, P13 and P15 state a qualifying horizon (the Five-Year Plan period); the remaining nine policies score 0.00 on X2.

X3 Policy Focus (5 items).

| **Item** | **Code 1 when the text…** |
| --- | --- |
| X3:1 Qualification requirements | states who may hold the post (education level, athletic grade, training/certification requirements) |
| X3:2 Scope of application | states which school types, levels, or organizations the policy covers |
| X3:3 Professional title system | makes provision on title ranks, title-review criteria, or correspondence to post grades. A bare statement that titles are "to be formulated separately" is coded 0 |
| X3:4 Job responsibilities | enumerates the duties of the coaching post |
| X3:5 Implementation measures | specifies concrete procedures for establishing posts, recruiting, or managing personnel |

X4 Policy Level (3 items). The final definition is a strict administrative hierarchy so each document occupies exactly one level.

| **Item** | **Code 1 when the issuing authority is…** |
| --- | --- |
| X4:1 National | a central ministry, commission, or the State Council / its offices |
| X4:2 Provincial | a province, autonomous region, or municipality directly under central government, or its departments |
| X4:3 Sub-provincial / affiliated institutions | a prefecture, city, district, or an institution directly subordinate to the issuing level |

X5 Policy Functions (6 items). Code 1 where substantive provisions perform the stated function: X5:1 Strengthening; X5:2 Guiding; X5:3 Encouraging; X5:4 Improving; X5:5 Carrying out; X5:6 Promoting. Coding is based on the function performed, not the presence of a corresponding verb.

X6 Participating Actors (3 items).

| **Item** | **Code 1 when the text assigns a role or task to…** |
| --- | --- |
| X6:1 Government | government bodies or departments (including co-issuing departments) |
| X6:2 Schools | schools or school administrative units |
| X6:3 Society | social organizations, enterprises, clubs, professional agencies, or service purchasing from them |

X7 Guarantee and Incentives (5 items).

| **Item** | **Code 1 when the text provides for…** |
| --- | --- |
| X7:1 Supervision and inspection | monitoring, inspection, assessment of implementation, or consequences of non-compliance |
| X7:2 Organizational leadership | leadership bodies, inter-departmental coordination mechanisms, or division of responsibility |
| X7:3 Publicity and guidance | publicity, awareness-raising, or dissemination arrangements |
| X7:4 Talent support | staffing quotas, funding, remuneration, career development, or training guarantees |
| X7:5 Others | other guarantee or incentive measures not covered above |

X8 Policy Evaluation (4 items).

| **Item** | **Code 1 when the document…** |
| --- | --- |
| X8:1 Clear objectives | states explicit, identifiable objectives |
| X8:2 Sufficient basis | cites the higher-level documents or legal basis on which it rests |
| X8:3 Scientific plan | sets out an internally consistent, staged or structured plan |
| X8:4 Detailed content | provides operationally specific content rather than general statements |

X9 Policy Domain (2 items). X9:1 Education is coded 1 when the document addresses the education sector; X9:2 Sports is coded 1 when it addresses the sports sector. All sampled policies score 1.00 on X9 because the sampling frame is inherently cross-sectoral.

# **S2.3 Revision to X4**

The original X4 mixed administrative rank and geographic type and could not classify the national document consistently. It was therefore redefined as National / Provincial / Sub-provincial. Provinces, autonomous regions, and municipalities directly under central government are treated as peers at the provincial level. All X4 values in the final analysis use this revised rule.

# **S2.4 Test–retest reliability and adjudication**

Coding was performed by a single coder. Reliability was therefore assessed by blind re-coding rather than independent double coding. The second pass covered all 12 policies × 38 indicators, and disagreements were resolved by re-reading the policy text against this manual.

The original coding retained first-level variable values rather than all first-pass item-level codes. Item-level agreement can therefore be calculated only for decisions whose first-pass values are reconstructible without ambiguity. The primary item-level figure is Po = 0.9100 on 200 determinate decisions across the seven variables whose rules were unchanged between coding passes; it is not an agreement rate over all 456 cells.

**Table S8. Test–retest agreement before adjudication**

| **Level of comparison** | **Basis** | **Agreement** |
| --- | --- | --- |
| Variable level, all nine variables | 108 policy × variable cells | 43.5% identical (47/108) |
| Variable level, seven unchanged-rule variables | 84 cells | 56.0% identical (47/84) |
| Item level, all nine variables | 227 determinate items | Po = 0.8414 |
| Item level, seven unchanged-rule variables | 200 determinate items | Po = 0.9100 |

X2 and X4 are shown separately because their decision rules were revised between coding passes; disagreements on those variables therefore reflect both rule revision and coding inconsistency.

Adjudication changed 32 of the 456 item cells (27 from 1 to 0 and 5 from 0 to 1), reducing positive codes from 343 to 321. The final item-level values, locators, and quoted warrants are published directly in Supplementary Material S4. All 321 positive warrants were checked against the corresponding policy text.
