# Supplementary material for: Evaluation of school sports coach position policies in China based on the PMC Index Model
Source: Front Public Health. 2026 Sep 18;14:1880356. doi: 10.3389/fpubh.2026.1880356 (PMC13630741; doi:10.3389/fpubh.2026.1880356)
Supplement: Supplementary file 3 [file Supplementary_file_3.docx]

**Supplementary Material S3. Policy Retrieval, Corpus Construction and Text Preprocessing**

This supplement documents the policy retrieval process, sample composition, corpus construction, preprocessing decisions, and the settings used for the final word-frequency and semantic-network analyses.

# **S3.1 Retrieval sources**

1. Central People's Government portal (www.gov.cn).

2. Central department portals: General Administration of Sport of China, Ministry of Education, and Ministry of Human Resources and Social Security.

3. Provincial-level department portals: provincial, autonomous-region and municipality sports, education, human-resources/social-security, and institutional-organization authorities.

4. PKUlaw database, used only to cross-check the existence and current version of documents located through official portals.

Initial retrieval was conducted in October–December 2024. A verification round in August 2026 re-obtained each retained document from an issuing-authority portal and checked the bibliographic fields used in the manuscript.

# **S3.2 Search terms**

| **Chinese term** | **English gloss** |
| --- | --- |
| 学校设置教练员岗位 | establishing coaching positions in schools |
| 学校体育教练员岗位设置 | establishment of school sports coach positions |
| 体育教练员专业技术岗位 | sports coach professional and technical posts |
| 退役运动员 担任 学校体育教师／教练员 | retired athletes serving as school PE teachers / coaches |

# **S3.3 Retrieval record for the scored sample**

Table S3 records the original Chinese titles, document numbers where verifiable, and dates used for the 12 policies included in PMC scoring. Unverified document numbers are left unstated rather than inferred.

**Table S3. Retrieval record for the retained scored sample**

| **ID** | **Region** | **Original Chinese title** | **Document number** | **Signature date** | **Published / effective** |
| --- | --- | --- | --- | --- | --- |
| P1 | National | 《关于在学校设置教练员岗位的实施意见》 | 体人规字〔2023〕3号 | 2023-01-16 | — |
| P2 | Anhui | 《关于做好从省优秀运动队退役运动员中选拔学校体育教师和体育教练员有关工作的通知》 | 皖体人〔2022〕9号 | 2022-01-18 | published 2022-02-08 |
| P3 | Fujian | 《福建省学校设置教练员岗位的实施办法》 | Not stated in available text | 2023-04-14 | — |
| P4 | Guangdong | 《关于在全省大中小学校设置体育教练员岗位的实施细则》 | 粤体规〔2024〕1号 | 2024-01-15 | — |
| P7 | Jiangsu | 《关于在学校设置教练员岗位的实施意见》 | 苏体规〔2023〕2号 | 2023-08-29 | published 2023-09-01; effective 2023-09-08 |
| P8 | Zhejiang | 《浙江省大中小学校体育教练员专业技术岗位设置管理实施意见》 | 浙体人〔2022〕275号 | 2022-10-12 | published 2022-10-20; effective 2022-12-01 |
| P9 | Jiangxi | 《江西省学校设置体育教练员专业技术岗位的实施意见》 | Not stated in available text | 2024-07-25 | — |
| P12 | Henan | 《河南省大中小学校教练员岗位设置管理的实施意见》 | Not stated in available text | 2023-08-01 | published 2023-08-16 |
| P13 | Jilin | 《吉林省大中小学校体育教练员专业技术岗位设置管理实施办法》 | Not stated in available text | 2023-08-25 | published 2023-09-13 |
| P15 | Inner Mongolia | 《关于在内蒙古自治区大中小学校设置体育教练员专业技术岗位的实施意见》 | Not stated in available text | 2023-12-21 | — |
| P17 | Chongqing | 《重庆市大中小学校设置教练员岗位实施方案》 | Not stated in available text | 2023-08-29 | — |
| P18 | Shanghai | 《关于上海市学校教练员专业技术岗位设置管理的实施意见》 | 沪体人〔2024〕82号 | 2024-04-30 | published 2024-05-08 |

Nine of the eleven local implementation policies cite the 2023 national document (体人规字〔2023〕3号) as their issuing authority. P2 and P8 predate it and instead cite the 2020 national policy documents.

# **S3.4 Corpus composition and file disposition**

The text-mining corpus contains the 11 retained local implementation policies. The national policy P1 is included in PMC scoring but excluded from the local-policy text corpus so national framework language does not alter local word frequencies. Records P11 and P16 were excluded from the study because full promulgated text could not be obtained.

**Table S4. Disposition of retrieved source files**

| **Source file** | **Disposition** | **ID** | **Region** | **Reason** |
| --- | --- | --- | --- | --- |
| 上海市.docx | included | P18 | Shanghai | — |
| 内蒙古自治区（含解读）.docx | included | P15 | Inner Mongolia | — |
| 吉林省.docx | included | P13 | Jilin | — |
| 安徽省.docx | included | P2 | Anhui | — |
| 广东省（含出台前后解读）.docx | included | P4 | Guangdong | — |
| 江苏省（含解读）.docx | included | P7 | Jiangsu | — |
| 江西省.docx | included | P9 | Jiangxi | — |
| 河南省.docx | included | P12 | Henan | — |
| 浙江省（含解读）.docx | included | P8 | Zhejiang | — |
| 福建省.docx | included | P3 | Fujian | — |
| 重庆市.docx | included | P17 | Chongqing | — |
| 广东省《…实施细则》.pdf | duplicate format | (P4) | Guangdong | Same policy as the .docx source; the .docx version was used |
| 福建省《…实施办法》.pdf | duplicate format | (P3) | Fujian | Same policy as the .docx source; the .docx version was used |
| 山东省（教练员晋级规定）.docx | quarantined | (P5) | Shandong | Professional-title evaluation standard issued as a draft for comment: not a position-establishment policy, and dated outside the study window |
| 湖北省（征求意见稿）.docx | quarantined | (P6) | Hubei | Draft issued for public comment; not formally promulgated |
| 甘肃省（政策解读…）.docx | quarantined | (P10) | Gansu | Policy interpretation only (republished by Baiyin Municipal Sports Bureau); the measures themselves not obtained |
| 广西自治区.docx | quarantined | (P14) | Guangxi | News summary plus a reply to an NPC representative's suggestion; no text of the measures |
| 浙江省…职称评价标准.doc | quarantined | — | Zhejiang | Professional-title evaluation standard, not a position-establishment policy (the Zhejiang policy itself is P8) |
| 安徽《…选拔…的通知》.pdf | quarantined | — | Anhui | Substantive text below the minimum-length threshold; no full policy text (the Anhui policy itself is P2) |
| 苏州：关于设置教练员岗位的答复.pdf | quarantined | — | Suzhou | Reply to a suggestion, not a policy document; sub-provincial authority not within the sampling frame |
| .DS_Store | skipped | — | — | System file |

# **S3.5 Preprocessing pipeline**

Stage 1 — Text extraction. Text was extracted while preserving paragraph structure.

Stage 2 — Deduplication. Duplicate file formats were collapsed, and repeated paragraphs were removed only within the same document. Cross-document deduplication was not used in the final corpus because local policies may legitimately reproduce central-policy language.

Stage 3 — Separation of interpretive material. Policy interpretations, Q&A material, replies, news text, and other commentary appended to policy files were excluded from the analysis corpus.

Stage 4 — Web-page furniture removal. Navigation text, view counters, index fields, print controls, source URLs, and similar portal material were removed.

Stage 5 — Noise stripping. HTML tags/entities, URLs, e-mail addresses, control characters, emoji, and non-policy formatting artefacts were removed.

Stage 6 — Character normalization. Full-width forms were normalised to half-width where appropriate while preserving substantive Chinese policy text.

Stage 7 — Navigation-line removal. Portal menu lines were identified structurally rather than by keyword so policy vocabulary was not deleted merely because it also appeared in navigation labels.

Stage 8 — Key-field check. Each retained document was checked for title, issuing authority, date, region, and a complete substantive policy body.

**Table S5. Per-document cleaning statistics**

| **ID** | **Region** | **Body chars** | **HTML** | **URL** | **Emoji** | **Web furniture** | **Full-width converted** | **Dup. paragraphs** | **Interpretive chars separated** | **Nav lines removed** | **Final chars** |
| --- | --- | --- | --- | --- | --- | --- | --- | --- | --- | --- | --- |
| P2 | Anhui | 2,512 | 0 | 0 | 0 | 10 | 95 | 2 | 0 | 0 | 2,461 |
| P3 | Fujian | 2,218 | 1 | 0 | 0 | 0 | 105 | 0 | 0 | 0 | 2,232 |
| P4 | Guangdong | 3,354 | 0 | 0 | 0 | 0 | 254 | 0 | 2,439 | 0 | 3,369 |
| P7 | Jiangsu | 2,513 | 0 | 0 | 0 | 0 | 207 | 1 | 1,164 | 4 | 2,446 |
| P8 | Zhejiang | 3,197 | 0 | 0 | 0 | 6 | 338 | 0 | 4,210 | 0 | 3,143 |
| P9 | Jiangxi | 3,196 | 1 | 1 | 0 | 11 | 153 | 0 | 0 | 8 | 3,091 |
| P12 | Henan | 2,413 | 0 | 0 | 0 | 0 | 124 | 0 | 0 | 0 | 2,385 |
| P13 | Jilin | 3,267 | 0 | 1 | 0 | 13 | 151 | 1 | 0 | 33 | 3,087 |
| P15 | Inner Mongolia | 3,101 | 0 | 0 | 0 | 2 | 186 | 0 | 1,628 | 0 | 3,073 |
| P17 | Chongqing | 1,972 | 0 | 0 | 0 | 0 | 101 | 0 | 0 | 0 | 1,980 |
| P18 | Shanghai | 2,882 | 0 | 1 | 0 | 4 | 137 | 2 | 0 | 4 | 2,868 |
| Total |  | 30,625 | 2 | 3 | 0 | 46 | 1,851 | 6 | 9,441 | 49 | 30,135 |

The final local-policy corpus contains 30,135 Chinese characters after cleaning and exclusion of interpretive material.

# **S3.6 Final text-analysis settings**

Word-frequency analysis. The cleaned corpus was segmented using a domain dictionary so policy-specific compounds such as 学校体育 (school sport), 体育教练员 (sports coach), 教练员岗位 (coaching position), 专业技术岗位 (professional and technical post), and 退役运动员 (retired athlete) were treated as single terms. Non-specific function words, general verbs/adjectives, and quantifier phrases were screened after segmentation. The final corpus produced 8,105 counted tokens and 1,049 distinct types; the 20 highest-frequency substantive terms are reported in Table 2 of the manuscript.

Semantic-network analysis. Co-occurrence was defined within a policy provision, operationalised by line breaks and sentence-final punctuation. The cleaned corpus produced 507 co-occurrence units with a median length of 52 characters. An edge is displayed when two terms co-occur in at least 30 provisions; node size reflects term frequency and edge width reflects co-occurrence frequency.

Revision note. The final Table 2 and Figure 2 were recomputed from the corrected 11-document local-policy corpus described above. The final corpus uses within-document deduplication and excludes interpretive material before both analyses.
