# Supplementary material for: Evaluation of school sports coach position policies in China based on the PMC Index Model
Source: Front Public Health. 2026 Sep 18;14:1880356. doi: 10.3389/fpubh.2026.1880356 (PMC13630741; doi:10.3389/fpubh.2026.1880356)
Supplement: Supplementary file 4 [file Supplementary_file_4.docx]

**Supplementary Material S4. Item-Level Coding Sheet and Adjudication Summary**

Manuscript: Evaluation of School Sports Coach Position Policies in China Based on the PMC Index Model

This supplement contains the final item-level coding used to calculate the PMC indices: all 456 policy-by-indicator cells (12 policies × 38 second-level indicators), with a locator and quoted warrant for every positive code. Coding rules are given in Supplementary Material S2 and policy retrieval/corpus procedures in Supplementary Material S3.

# **S4.1 Sample and indicator structure**

| **Code** | **Region / issuing level** | **Indicators coded** |
| --- | --- | --- |
| P1 | National (four central departments) | 38 |
| P2 | Anhui Province | 38 |
| P3 | Fujian Province | 38 |
| P4 | Guangdong Province | 38 |
| P7 | Jiangsu Province | 38 |
| P8 | Zhejiang Province | 38 |
| P9 | Jiangxi Province | 38 |
| P12 | Henan Province | 38 |
| P13 | Jilin Province | 38 |
| P15 | Inner Mongolia Autonomous Region | 38 |
| P17 | Chongqing Municipality | 38 |
| P18 | Shanghai Municipality | 38 |

First-level variables and indicator counts: X1 Policy Nature (6); X2 Policy Timeliness (4); X3 Policy Focus (5); X4 Policy Level (3); X5 Policy Functions (6); X6 Participating Actors (3); X7 Guarantee and Incentives (5); X8 Policy Evaluation (4); X9 Policy Domain (2).

# **S4.2 Final coding sheet**

A value of 1 indicates that the promulgated policy text satisfies the corresponding rule in S2; 0 indicates absence or unresolved ambiguity. Locators refer to the promulgated policy text. The values below are the final post-adjudication codes used in the manuscript.

## **P1 — National (four central departments)**

| **Indicator** | **Meaning** | **Code** | **Locator** | **Quoted warrant** |
| --- | --- | --- | --- | --- |
| X1:1 | Forecasting | 0 | — | — |
| X1:2 | Regulation | 1 | Art. 3, Establishment of posts (三、岗位设置) | 各地人力资源社会保障部门应在专业技术岗位总量及结构比例方面给予支持。 |
| X1:3 | Recommendation | 1 | Art. 8, Organization and implementation (八、组织实施) | 各地可根据本意见研究制定实施细则,鼓励各地先行先试,积极探索,制定符合本地实际的学校体育工作机制和学校教练员职称评价标准体系。 |
| X1:4 | Description | 1 | Art. 8, Organization and implementation (八、组织实施) | 在学校设置教练员岗位是对学校体育工作力量的有力加强,是体教融合的重要举措,事关青少年身心健康和全面发展。 |
| X1:5 | Guidance | 1 | Art. 1, Guiding principles (一、指导思想) | 一、指导思想 以习近平新时代中国特色社会主义思想为指导,全面贯彻党的教育方针,以服务学生全面发展、增强综合素质为目标,坚持健康第一的教育理念,通过在学校工作的教练员(以下简称“学校教练员”)加强学校体育工作力量,提升青少年体育锻炼质量和… |
| X1:6 | Others | 0 | — | — |
| X2:1 | Short-term | 0 | — | — |
| X2:2 | Medium-term | 0 | — | — |
| X2:3 | Long-term | 0 | — | — |
| X2:4 | Within current year | 0 | — | — |
| X3:1 | Qualification requirements | 1 | Art. 6, Qualification requirements (六、任职条件) | 六、任职条件 学校教练员应具备以下基本条件: |
| X3:2 | Scope of application | 1 | Art. 2, Scope of application (二、适用范围) | 二、适用范围 本意见适用于义务教育阶段学校、普通高中、职业院校、普通高校。 |
| X3:3 | Professional title system | 1 | Art. 5, Professional title system (五、职称体系) | 五、职称体系 学校教练员的职称层级、岗位等级和评价标准按照《人力资源社会保障部 体育总局关于深化体育专业人员职称制度改革的指导意见》(人社部发〔2020〕76号)有关规定执行。 |
| X3:4 | Job responsibilities | 1 | Art. 4, Duties of the post (四、岗位职责) | 四、岗位职责 学校教练员按照学校体育工作计划,发挥专业特长,参与体育教学和训练工作。 |
| X3:5 | Implementation measures | 1 | Art. 7, Appointment to posts (七、岗位聘用) | 七、岗位聘用 (一)学校按现有规定程序要求制定学校教练员岗位设置实施方案,根据按需设岗、公开招聘、择优聘用的原则,开展岗位聘用工作。 |
| X4:1 | National | 1 | Issuing authority / title (发文机关/标题) | 体育总局 中央编办 教育部 人力资源社会保障部 |
| X4:2 | Provincial | 0 | — | — |
| X4:3 | Sub-provincial or affiliated | 0 | — | — |
| X5:1 | Strengthening | 1 | Title / transmittal notice (标题/发文通知) | 为深入贯彻落实中共中央办公厅、国务院办公厅《关于全面加强和改进新时代学校体育工作的意见》(中办发〔2020〕36号)和《体育总局 教育部关于印发深化体教融合 促进青少年健康发展意见的通知》(体发〔2020〕1号)精神,进一步加强学校体育… |
| X5:2 | Guiding | 0 | — | — |
| X5:3 | Encouraging | 1 | Art. 8, Organization and implementation (八、组织实施) | 各地可根据本意见研究制定实施细则,鼓励各地先行先试,积极探索,制定符合本地实际的学校体育工作机制和学校教练员职称评价标准体系。 |
| X5:4 | Improving | 0 | — | — |
| X5:5 | Carrying out | 1 | Art. 7, Appointment to posts (七、岗位聘用) | (一)学校按现有规定程序要求制定学校教练员岗位设置实施方案,根据按需设岗、公开招聘、择优聘用的原则,开展岗位聘用工作。 |
| X5:6 | Promoting | 1 | Title / transmittal notice (标题/发文通知) | 为深入贯彻落实中共中央办公厅、国务院办公厅《关于全面加强和改进新时代学校体育工作的意见》(中办发〔2020〕36号)和《体育总局 教育部关于印发深化体教融合 促进青少年健康发展意见的通知》(体发〔2020〕1号)精神,进一步加强学校体育… |
| X6:1 | Government | 1 | Art. 7, Appointment to posts (七、岗位聘用) | (三)体育部门负责做好退役运动员转型学校教练员培训工作,教育部门在学校教练员入职后加强思想政治、职业道德和教学培训,提高教育教学能力水平。 |
| X6:2 | Schools | 1 | Title / transmittal notice (标题/发文通知) | 体育总局 中央编办 教育部 人力资源社会保障部关于在学校设置教练员岗位的实施意见 |
| X6:3 | Society | 1 | Art. 3, Establishment of posts (三、岗位设置) | 有条件的地区可以通过购买服务方式,与相关专业机构等社会力量合作向学校提供体育教育教学服务,缓解体育师资不足问题。 |
| X7:1 | Supervision and inspection | 0 | — | — |
| X7:2 | Organizational leadership | 1 | Art. 8, Organization and implementation (八、组织实施) | 各地要高度重视,加强组织领导。 |
| X7:3 | Publicity and guidance | 0 | — | — |
| X7:4 | Talent support | 1 | Art. 3, Establishment of posts (三、岗位设置) | 确有必要设立专职教练员岗位的学校,在核定的编制和专业技术岗位总量及结构比例内设置,专岗专用,纳入专业技术岗位进行管理。 |
| X7:5 | Others | 0 | — | — |
| X8:1 | Clear objectives | 0 | — | — |
| X8:2 | Sufficient basis | 1 | Art. 3, Establishment of posts (三、岗位设置) | 学校可根据工作实际,设立专(兼)职教练员岗位。 |
| X8:3 | Scientific plan | 0 | — | — |
| X8:4 | Detailed content | 1 | Art. 6, Qualification requirements (六、任职条件) | 六、任职条件 学校教练员应具备以下基本条件: (一)具有良好的思想政治素质和道德品质,遵纪守法,遵守职业道德规范,身心健康,举止文明。 (二)热爱教育事业,为人师表,关爱学生,遵循教育规律和学生成长规律。 (三)热爱体育事业,了解相应运… |
| X9:1 | Education | 1 | Title / transmittal notice (标题/发文通知) | 体育总局 中央编办 教育部 人力资源社会保障部关于在学校设置教练员岗位的实施意见 |
| X9:2 | Sports | 1 | Title / transmittal notice (标题/发文通知) | 体育总局 中央编办 教育部 人力资源社会保障部关于在学校设置教练员岗位的实施意见 |

First-level values: X1 0.67; X2 0.00; X3 1.00; X4 0.33; X5 0.67; X6 1.00; X7 0.40; X8 0.50; X9 1.00. PMC index 5.57; concavity index 3.43.

## **P2 — Anhui Province**

| **Indicator** | **Meaning** | **Code** | **Locator** | **Quoted warrant** |
| --- | --- | --- | --- | --- |
| X1:1 | Forecasting | 1 | Art. 6, Strengthening support measures (六、强化保障措施) | (一)各地要加大力度配齐配强大中小学体育专业师资队伍,未配齐的地区和学校每年应划出一定比例用于招聘体育教师和体育教练员。 |
| X1:2 | Regulation | 1 | Art. 3, Sports coach posts (三、体育教练员岗位) | 共建、联办高水平运动队的学校,建有高水平优秀运动队的高等院校,应根据运动项目需要适当增加体育教练员岗位。 |
| X1:3 | Recommendation | 1 | Art. 5, Recruitment (五、人员招聘) | 鼓励各地各校面向我省具有相应教师资格的优秀运动队退役运动员定向招聘体育教师。 |
| X1:4 | Description | 1 | Title / transmittal notice (标题/发文通知) | 为贯彻落实《中央办公厅 国务院办公厅关于全面加强和改进新时代学校体育工作的意见》(中办发〔2020〕36号)、《国家体育总局 教育部关于印发深化体教融合 促进青少年健康发展意见的通知》(体发〔2020〕1号)和省政府《关于印发体育强省建… |
| X1:5 | Guidance | 1 | Art. 1, General requirements (一、总体要求) | 以习近平新时代中国特色社会主义思想为指导,全面贯彻党的教育方针,坚持健康第一的教育理念,充分发挥党委领导和政府主导作用,深化体教融合,以服务学生全面发展、增强综合素质为目标,积极引导优秀运动队退役运动员从事学校体育工作,充实优化我省学校… |
| X1:6 | Others | 0 | — | — |
| X2:1 | Short-term | 0 | — | — |
| X2:2 | Medium-term | 0 | — | — |
| X2:3 | Long-term | 0 | — | — |
| X2:4 | Within current year | 0 | — | — |
| X3:1 | Qualification requirements | 1 | Art. 4, Duties and qualification requirements (四、岗位职责与任职条件) | 四、岗位职责与任职条件 体育教练员主要负责学校专项体育运动工作计划的制定与实施;负责组织实施青少年学生健康体适能训练、体育专项技能培训;负责组织青少年学生开展体育活动和竞赛活动;负责体育运动康复等理论知识及技术技能的传授;负责优秀体育后… |
| X3:2 | Scope of application | 1 | Art. 2, Scope of application (二、适用范围) | 二、适用范围 全省各级各类公办学校,包括义务教育学校、普通高中、中等职业学校、高等学校、各级各类体校等,适用本通知。 |
| X3:3 | Professional title system | 0 | — | — |
| X3:4 | Job responsibilities | 1 | Art. 4, Duties and qualification requirements (四、岗位职责与任职条件) | 体育教练员主要负责学校专项体育运动工作计划的制定与实施;负责组织实施青少年学生健康体适能训练、体育专项技能培训;负责组织青少年学生开展体育活动和竞赛活动;负责体育运动康复等理论知识及技术技能的传授;负责优秀体育后备人才的发现和培养。 |
| X3:5 | Implementation measures | 1 | Art. 5, Recruitment (五、人员招聘) | 各地各校应在岗位总量和结构比例内,根据按需设岗、公开招聘、竞聘上岗、择优聘用的原则,开展体育教练员聘用工作。 |
| X4:1 | National | 0 | — | — |
| X4:2 | Provincial | 1 | Issuing authority / signature block (发文机关/落款) | 安徽省教育厅 安徽省人力资源和社会保障厅 安徽省体育局 皖体人〔2022〕9号 |
| X4:3 | Sub-provincial or affiliated | 0 | — | — |
| X5:1 | Strengthening | 1 | Title / transmittal notice (标题/发文通知) | 为贯彻落实《中央办公厅 国务院办公厅关于全面加强和改进新时代学校体育工作的意见》(中办发〔2020〕36号)、《国家体育总局 教育部关于印发深化体教融合 促进青少年健康发展意见的通知》(体发〔2020〕1号)和省政府《关于印发体育强省建… |
| X5:2 | Guiding | 0 | — | — |
| X5:3 | Encouraging | 1 | Art. 5, Recruitment (五、人员招聘) | 鼓励各地各校面向我省具有相应教师资格的优秀运动队退役运动员定向招聘体育教师。 |
| X5:4 | Improving | 1 | Art. 6, Strengthening support measures (六、强化保障措施) | 同时,各级体育、教育行政部门要畅通体育教师序列与教练员序列职称转评机制,完善教练员职称评聘标准。 |
| X5:5 | Carrying out | 1 | Art. 4, Duties and qualification requirements (四、岗位职责与任职条件) | 负责组织青少年学生开展体育活动和竞赛活动;负责体育运动康复等理论知识及技术技能的传授;负责优秀体育后备人才的发现和培养。 |
| X5:6 | Promoting | 1 | Title / transmittal notice (标题/发文通知) | 为贯彻落实《中央办公厅 国务院办公厅关于全面加强和改进新时代学校体育工作的意见》(中办发〔2020〕36号)、《国家体育总局 教育部关于印发深化体教融合 促进青少年健康发展意见的通知》(体发〔2020〕1号)和省政府《关于印发体育强省建… |
| X6:1 | Government | 1 | Art. 6, Strengthening support measures (六、强化保障措施) | 省级体育、教育部门负责组织实施,组织学习教育学、教育心理学等教育基础知识及教育实践课程,提升教学能力水平和教育综合素质。 |
| X6:2 | Schools | 1 | Title / transmittal notice (标题/发文通知) | 选拔学校体育教师和体育教练员有关工作的通知 |
| X6:3 | Society | 0 | — | — |
| X7:1 | Supervision and inspection | 1 | Art. 7, Strengthening organizational leadership (七、切实加强组织领导) | 对未按规定配备学校体育教练员的,视情况取消体育传统项目学校、体育特色学校、校园足球特色学校称号、高等院校高水平运动员招收资格等。 |
| X7:2 | Organizational leadership | 1 | Art. 7, Strengthening organizational leadership (七、切实加强组织领导) | 学校体育教练员人才队伍建设,事关全省青少年身心健康全面发展,事关教育体育人才资源配置和全省学校体育工作成效,各级教育、人社、体育行政部门要统筹联合、分工协作、精心组织,及时研究解决工作中出现的各类情况和问题,确保学校体育工作持续健康发展。 |
| X7:3 | Publicity and guidance | 1 | Art. 1, General requirements (一、总体要求) | 以习近平新时代中国特色社会主义思想为指导,全面贯彻党的教育方针,坚持健康第一的教育理念,充分发挥党委领导和政府主导作用,深化体教融合,以服务学生全面发展、增强综合素质为目标,积极引导优秀运动队退役运动员从事学校体育工作,充实优化我省学校… |
| X7:4 | Talent support | 1 | Art. 2, Scope of application (二、适用范围) | 所称优秀运动队退役运动员,是指纳入我省机构编制部门管理,经省人力资源和社会保障厅批准,由省体育局招聘到省优秀运动队,专业从事某项体育运动训练并参加比赛,取得良好运动成绩或运动技术等级,并已办理停训退役手续的运动员。 |
| X7:5 | Others | 0 | — | — |
| X8:1 | Clear objectives | 0 | — | — |
| X8:2 | Sufficient basis | 1 | Art. 3, Sports coach posts (三、体育教练员岗位) | 全省各级各类公办学校根据学校体育工作需要,在核定的事业单位岗位总量及专业技术岗位结构比例内,设置体育教练员岗位,纳入专业技术岗位序列管理。 |
| X8:3 | Scientific plan | 1 | Art. 3, Sports coach posts (三、体育教练员岗位) | 各级各类学校可根据师生比例和发展需要设置一定数量体育教练员专业技术岗位。属于高水平体育后备人才基地学校、体育传统项目学校、体育特色学校、校园足球特色学校等,体育教练员岗位数量一般不少于2个。共建、联办高水平运动队的学校,建有高水平优秀运动队的高等院校,应根据运动项目需要适当增加体育教练员岗位。 |
| X8:4 | Detailed content | 1 | Art. 4, Duties and qualification requirements (四、岗位职责与任职条件) | 四、岗位职责与任职条件 体育教练员主要负责学校专项体育运动工作计划的制定与实施;负责组织实施青少年学生健康体适能训练、体育专项技能培训;负责组织青少年学生开展体育活动和竞赛活动;负责体育运动康复等理论知识及技术技能的传授;负责优秀体育后… |
| X9:1 | Education | 1 | Title / transmittal notice (标题/发文通知) | 各市教育局、人力资源社会保障局、体育行政部门,各省属高等学校及有关单位: |
| X9:2 | Sports | 1 | Title / transmittal notice (标题/发文通知) | 省体育局 |

First-level values: X1 0.83; X2 0.00; X3 0.80; X4 0.33; X5 0.83; X6 0.67; X7 0.80; X8 0.75; X9 1.00. PMC index 6.02; concavity index 2.98.

## **P3 — Fujian Province**

| **Indicator** | **Meaning** | **Code** | **Locator** | **Quoted warrant** |
| --- | --- | --- | --- | --- |
| X1:1 | Forecasting | 0 | — | — |
| X1:2 | Regulation | 1 | Art. 3, Establishment of posts (三、岗位设置) | 各地人力资源社会保障部门应在专业技术岗位总量及结构比例方面给予支持。 |
| X1:3 | Recommendation | 1 | Art. 8, Organization and implementation (八、组织实施) | 支持和鼓励市、县(区)根据发展需要,研究确定将具有重大突出贡献的优秀退役运动员纳入人才引进等政策范围。 |
| X1:4 | Description | 1 | Title / transmittal notice (标题/发文通知) | 为贯彻落实中共中央办公厅、国务院办公厅《关于全面加强和改进新时代学校体育工作的意见》(中办发〔2020〕36 号)、《体育总局 中央编办 教育部 人力资源社会保障部关于在学校设置教练员岗位的实施意见》(体人规字〔2023〕3 号)和《体… |
| X1:5 | Guidance | 1 | Art. 1, Guiding principles (一、指导思想) | 一、指导思想 以习近平新时代中国特色社会主义思想为指导,全面贯彻党的教育方针,以服务学生全面发展、增强综合素质为目标,坚持健康第一的教育理念,通过在学校工作的教练员加强学校体育工作力量,提升青少年体育锻炼质量和水平,帮助青少年享受乐趣、… |
| X1:6 | Others | 1 | Art. 9, Commencement and authority for interpretation (九、本实施意见自印发之日起实施,由省委编办、省教育厅、省人社厅、省体育局按照职能职责负责解释。) | 九、本实施意见自印发之日起实施,由省委编办、省教育厅、省人社厅、省体育局按照职能职责负责解释。 |
| X2:1 | Short-term | 0 | — | — |
| X2:2 | Medium-term | 0 | — | — |
| X2:3 | Long-term | 0 | — | — |
| X2:4 | Within current year | 0 | — | — |
| X3:1 | Qualification requirements | 1 | Art. 6, Qualification requirements (六、任职条件) | 六、任职条件 学校教练员岗位的基本任职条件: |
| X3:2 | Scope of application | 1 | Art. 2, Scope of application (二、适用范围) | 二、适用范围 全省义务教育阶段学校、普通高中、职业院校、普通高校。 |
| X3:3 | Professional title system | 1 | Art. 5, Professional title system (五、职称体系) | 学校教练员的职称层级、岗位等级和评价标准按照《人力资源社会保障部 体育总局关于深化体育专业人员职称制度改革的指导意见》(人社部发〔2020〕76号)有关规定执行。学校教练员执教期间,学生体质和运动能力提升情况、体育后备人才培养情况、学校体育赛事活动组织情况、学校体育社团管理情况等,均可作为其职称评审有效业绩。 |
| X3:4 | Job responsibilities | 1 | Art. 4, Duties of the post (四、岗位职责) | 四、岗位职责 学校教练员按照学校体育工作计划,发挥专业特长,参与体育教学和训练工作。 |
| X3:5 | Implementation measures | 1 | Art. 7, Appointment to posts (七、岗位聘用) | 七、岗位聘用 (一)学校在岗位总量和结构比例内,按照学校岗位设置实施方案,根据按需设岗、公开招聘、择优聘用的原则,开展教练员岗位聘用工作。 |
| X4:1 | National | 0 | — | — |
| X4:2 | Provincial | 1 | Art. 9, Authority for interpretation (九、解释机关) | 九、本实施意见自印发之日起实施,由省委编办、省教育厅、省人社厅、省体育局按照职能职责负责解释。 |
| X4:3 | Sub-provincial or affiliated | 0 | — | — |
| X5:1 | Strengthening | 1 | Title / transmittal notice (标题/发文通知) | 为贯彻落实中共中央办公厅、国务院办公厅《关于全面加强和改进新时代学校体育工作的意见》(中办发〔2020〕36 号)、《体育总局 中央编办 教育部 人力资源社会保障部关于在学校设置教练员岗位的实施意见》(体人规字〔2023〕3 号)和《体… |
| X5:2 | Guiding | 0 | — | — |
| X5:3 | Encouraging | 1 | Art. 8, Organization and implementation (八、组织实施) | 支持和鼓励市、县(区)根据发展需要,研究确定将具有重大突出贡献的优秀退役运动员纳入人才引进等政策范围。 |
| X5:4 | Improving | 0 | — | — |
| X5:5 | Carrying out | 1 | Art. 7, Appointment to posts (七、岗位聘用) | (一)学校在岗位总量和结构比例内,按照学校岗位设置实施方案,根据按需设岗、公开招聘、择优聘用的原则,开展教练员岗位聘用工作。 |
| X5:6 | Promoting | 1 | Title / transmittal notice (标题/发文通知) | 为贯彻落实中共中央办公厅、国务院办公厅《关于全面加强和改进新时代学校体育工作的意见》(中办发〔2020〕36 号)、《体育总局 中央编办 教育部 人力资源社会保障部关于在学校设置教练员岗位的实施意见》(体人规字〔2023〕3 号)和《体… |
| X6:1 | Government | 1 | Art. 8, Organization and implementation (八、组织实施) | (二)体育部门负责做好退役运动员转型学校教练员培训工作,可推荐优秀退役运动员担任学校实习教练员(实习期不超过1 年);教育部门在学校教练员入职后加强思想政治、职业道德和教学培训,提高教育教学能力水平。 |
| X6:2 | Schools | 1 | Title / transmittal notice (标题/发文通知) | 福建省学校设置教练员岗位的实施办法 |
| X6:3 | Society | 1 | Art. 3, Establishment of posts (三、岗位设置) | 有条件的地区可以通过购买服务方式,与相关专业机构等社会力量合作向学校提供体育教育教学服务,缓解体育师资不足问题。 |
| X7:1 | Supervision and inspection | 1 | Art. 8, Organization and implementation (八、组织实施) | (一)学校教练员人才队伍建设,事关全省青少年身心健康全面发展和竞技体育优秀后备人才培养成效,各级机构编制、教育、人力资源社会保障和体育等部门要加强组织领导,强化部门职责,分工协作、联合督导,及时研究解决工作中出现的情况和问题。 |
| X7:2 | Organizational leadership | 1 | Art. 8, Organization and implementation (八、组织实施) | (一)学校教练员人才队伍建设,事关全省青少年身心健康全面发展和竞技体育优秀后备人才培养成效,各级机构编制、教育、人力资源社会保障和体育等部门要加强组织领导,强化部门职责,分工协作、联合督导,及时研究解决工作中出现的情况和问题。 |
| X7:3 | Publicity and guidance | 0 | — | — |
| X7:4 | Talent support | 1 | Art. 3, Establishment of posts (三、岗位设置) | 被命名为省级及以上“高水平体育后备人才基地学校”和“体育传统特色学校”、具有高水平运动队招生资格的高等院校,新增1 个及以上编制用于设置专职学校教练员岗位招聘取得一级及以上运动员技术等级的退役运动员,专岗专用,纳入专业技术岗位进行管理。 |
| X7:5 | Others | 1 | Art. 9, Commencement and authority for interpretation (九、本实施意见自印发之日起实施,由省委编办、省教育厅、省人社厅、省体育局按照职能职责负责解释。) | 九、本实施意见自印发之日起实施,由省委编办、省教育厅、省人社厅、省体育局按照职能职责负责解释。 |
| X8:1 | Clear objectives | 0 | — | — |
| X8:2 | Sufficient basis | 1 | Art. 3, Establishment of posts (三、岗位设置) | 学校可根据工作实际,设立专(兼)职教练员岗位。 |
| X8:3 | Scientific plan | 0 | — | — |
| X8:4 | Detailed content | 1 | Art. 6, Qualification requirements (六、任职条件) | 学校教练员岗位的基本任职条件: (一)具有良好的思想政治素质和道德品质,遵纪守法,遵守职业道德规范,身心健康,举止文明。(二)热爱教育事业,为人师表,关爱学生,遵循教育规律和学生成长规律。(三)热爱体育事业,了解相应运动项目的竞赛规程及裁判规则,熟悉相应年龄段学生的运动生理、心理特点。 |
| X9:1 | Education | 1 | Title / transmittal notice (标题/发文通知) | 为贯彻落实中共中央办公厅、国务院办公厅《关于全面加强和改进新时代学校体育工作的意见》(中办发〔2020〕36 号)、《体育总局 中央编办 教育部 人力资源社会保障部关于在学校设置教练员岗位的实施意见》(体人规字〔2023〕3 号)和《体… |
| X9:2 | Sports | 1 | Title / transmittal notice (标题/发文通知) | 为贯彻落实中共中央办公厅、国务院办公厅《关于全面加强和改进新时代学校体育工作的意见》(中办发〔2020〕36 号)、《体育总局 中央编办 教育部 人力资源社会保障部关于在学校设置教练员岗位的实施意见》(体人规字〔2023〕3 号)和《体… |

First-level values: X1 0.83; X2 0.00; X3 1.00; X4 0.33; X5 0.67; X6 1.00; X7 0.80; X8 0.50; X9 1.00. PMC index 6.13; concavity index 2.87.

## **P4 — Guangdong Province**

| **Indicator** | **Meaning** | **Code** | **Locator** | **Quoted warrant** |
| --- | --- | --- | --- | --- |
| X1:1 | Forecasting | 0 | — | — |
| X1:2 | Regulation | 1 | Art. 1, Scope of establishment (一、设置范围) | (二)各类国家级体育后备人才基地学校,省级以上体育传统特色学校,体教合作共建学校,承担全国以上运动会备战任务的学校,义务教育阶段未达到体育教师配比的学校,招收运动训练专业、武术与民族传统体育专业的普通高等院校,招收高水平运动队的普通高等… |
| X1:3 | Recommendation | 1 | Art. 7, Support measures (七、保障措施) | 鼓励各级各类体校与体育传统特色学校合作共建,实施“一校两牌”。 |
| X1:4 | Description | 1 | Art. 7, Support measures (七、保障措施) | 省优秀运动队退役运动员,在奥运会上取得前八名和亚运会奥运项目上取得前六名,符合学校体育教练员任职条件的,由人事关系所在地按照有关规定在设置体育教练员岗位的公办学校给予政策性安置。 |
| X1:5 | Guidance | 1 | Art. 8, Organization and implementation (八、组织实施) | 各地市、县(区)要高度重视,坚持“健康第一”的教育理念,通过在学校设置体育教练员岗位,推动广大青少年文化学习和体育锻炼协调发展。 |
| X1:6 | Others | 1 | Art. 8, Organization and implementation (八、组织实施) | 本细则自2024年2月20日起实施,有效期5年,由省体育局、省教育厅会同有关部门负责解释。 |
| X2:1 | Short-term | 0 | — | — |
| X2:2 | Medium-term | 0 | — | — |
| X2:3 | Long-term | 0 | — | — |
| X2:4 | Within current year | 0 | — | — |
| X3:1 | Qualification requirements | 1 | Art. 4, Qualification requirements (四、任职条件) | 四、任职条件 任职学校体育教练员应具备以下条件: |
| X3:2 | Scope of application | 0 | — | — |
| X3:3 | Professional title system | 1 | Art. 5, Professional title system (五、职称体系) | 五、职称体系 学校体育教练员职称纳入体育专业人员职称系列,职称层级、岗位等级和评价标准按照《人力资源社会保障部 体育总局关于深化体育专业人员职称制度改革的指导意见》(人社部发〔2020〕76号)有关规定执行。 |
| X3:4 | Job responsibilities | 1 | Art. 3, Duties of the post (三、岗位职责) | 三、岗位职责 学校体育教练员按照学校体育工作计划,发挥专业特长,具体负责运动专项训练和竞赛组织工作,协助开展学校体育教学。 |
| X3:5 | Implementation measures | 1 | Art. 6, Appointment to posts (六、岗位聘用) | 六、岗位聘用 (一)学校按现有规定程序要求制定学校体育教练员岗位设置实施方案,根据按需设岗、公开招聘、择优聘用的原则,开展岗位聘用工作。 |
| X4:1 | National | 0 | — | — |
| X4:2 | Provincial | 1 | Issuing authority / signature block (发文机关/落款) | 广东省体育局 中共广东省委机构编制委员会办公室 广东省教育厅 广东省人力资源和社会保障厅 粤体规〔2024〕1号 |
| X4:3 | Sub-provincial or affiliated | 0 | — | — |
| X5:1 | Strengthening | 1 | Title / transmittal notice (标题/发文通知) | 根据《中华人民共和国体育法》关于在学校设立体育教练员岗位的相关规定,依据中共中央办公厅、国务院办公厅《关于全面加强和改进新时代学校体育工作的意见》(中办发〔2020〕36号)、《体育总局 教育部关于印发深化体教融合 促进青少年健康发展意… |
| X5:2 | Guiding | 0 | — | — |
| X5:3 | Encouraging | 1 | Art. 7, Support measures (七、保障措施) | 鼓励各级各类体校与体育传统特色学校合作共建,实施“一校两牌”。 |
| X5:4 | Improving | 1 | Art. 7, Support measures (七、保障措施) | (一)完善项目布局,搭建学校体育教练员作用发挥平台。 |
| X5:5 | Carrying out | 1 | Art. 3, Duties of the post (三、岗位职责) | 学校体育教练员按照学校体育工作计划,发挥专业特长,具体负责运动专项训练和竞赛组织工作,协助开展学校体育教学。 |
| X5:6 | Promoting | 1 | Title / transmittal notice (标题/发文通知) | 根据《中华人民共和国体育法》关于在学校设立体育教练员岗位的相关规定,依据中共中央办公厅、国务院办公厅《关于全面加强和改进新时代学校体育工作的意见》(中办发〔2020〕36号)、《体育总局 教育部关于印发深化体教融合 促进青少年健康发展意… |
| X6:1 | Government | 1 | Art. 6, Appointment to posts (六、岗位聘用) | (三)体育部门做好退役运动员转型学校体育教练员培训工作,教育部门在学校体育教练员入职后加强思想政治、职业道德和教学培训,提高教育教学能力水平。 |
| X6:2 | Schools | 1 | Title / transmittal notice (标题/发文通知) | 全省大中小学校设置体育教练员 |
| X6:3 | Society | 1 | Art. 2, Establishment of posts (二、岗位设置) | 可以通过购买服务的方式,与相关专业机构等社会力量合作向学校提供体育教育教学服务,缓解体育师资不足问题。 |
| X7:1 | Supervision and inspection | 1 | Art. 8(3), Formulation of implementation plans (八、组织实施（三）制定实施方案) | (三)制定实施方案。各地市、县(区)可依据本细则研究制定实施方案,立足实际、积极探索、统筹推进,不断完善学校体育工作机制和考核评价体系。 |
| X7:2 | Organizational leadership | 1 | Art. 8, Organization and implementation (八、组织实施) | (二)加强组织领导。 |
| X7:3 | Publicity and guidance | 1 | Art. 7, Support measures (七、保障措施) | 积极引导各级各类学校建立相应项目运动队,体育传统特色学校、体教合作共建学校应当建立高水平运动队。 |
| X7:4 | Talent support | 1 | Art. 2, Establishment of posts (二、岗位设置) | 全省大中小学校根据实际工作情况设置专(兼)职体育教练员岗位,专岗专用,按规定纳入专业技术岗位进行管理。有条件的地区,可以适当增加编制或在专业技术岗位总量及结构比例范围内统筹;可以通过购买服务的方式,与相关专业机构等社会力量合作向学校提供体育教育教学服务,缓解体育师资不足问题。 |
| X7:5 | Others | 1 | Art. 8, Organization and implementation (八、组织实施) | 本细则自2024年2月20日起实施,有效期5年,由省体育局、省教育厅会同有关部门负责解释。 |
| X8:1 | Clear objectives | 0 | — | — |
| X8:2 | Sufficient basis | 1 | Title / transmittal notice (标题/发文通知) | 根据《中华人民共和国体育法》关于在学校设立体育教练员岗位的相关规定,依据中共中央办公厅、国务院办公厅《关于全面加强和改进新时代学校体育工作的意见》(中办发〔2020〕36号)、《体育总局 教育部关于印发深化体教融合 促进青少年健康发展意… |
| X8:3 | Scientific plan | 1 | Art. 1, Scope of establishment (一、设置范围) | 一、设置范围 (一)全省义务教育阶段学校、普通高中、职业院校、普通高校根据实际需要,增设体育教练员岗位。 |
| X8:4 | Detailed content | 1 | Art. 4, Qualification requirements (四、任职条件) | 四、任职条件 任职学校体育教练员应具备以下条件: (一)基本条件。 (二)学历及专业条件。 任职学校体育教练员应具备大学本科以上学历,具有省级以上优秀运动队、职业体育俱乐部运动队、高等院校高水平运动队、地市级以上体育运动学校等三年以上专… |
| X9:1 | Education | 1 | Title / transmittal notice (标题/发文通知) | 委员会办公室 广东省教育厅 广东省 |
| X9:2 | Sports | 1 | Title / transmittal notice (标题/发文通知) | 广东省体育局 中共广东省委机构编制 |

First-level values: X1 0.83; X2 0.00; X3 0.80; X4 0.33; X5 0.83; X6 1.00; X7 1.00; X8 0.75; X9 1.00. PMC index 6.55; concavity index 2.45.

## **P7 — Jiangsu Province**

| **Indicator** | **Meaning** | **Code** | **Locator** | **Quoted warrant** |
| --- | --- | --- | --- | --- |
| X1:1 | Forecasting | 0 | — | — |
| X1:2 | Regulation | 1 | Art. 3, Establishment of posts (三、岗位设置) | 各级人力资源社会保障部门应在专业技术岗位总量及结构比例方面给予支持。 |
| X1:3 | Recommendation | 1 | Art. 3, Establishment of posts (三、岗位设置) | 鼓励各级各类学校通过购买服务方式,与相关专业机构等社会力量合作,缓解体育师资不足的问题。 |
| X1:4 | Description | 1 | Art. 8, Organization and implementation (八、组织实施) | (一)在学校设置教练员岗位是对学校体育工作的有力加强,是体教融合的重要举措,事关全省青少年身心健康和全面发展。 |
| X1:5 | Guidance | 1 | Art. 1, Guiding principles (一、指导思想) | 一、指导思想 以习近平新时代中国特色社会主义思想为指导,以服务学生全面发展、增强综合素质为目标,全面贯彻党的教育方针,坚持健康第一的教育理念,通过在学校工作的教练员(以下简称“学校教练员”)加强学校体育工作力量,提升青少年体育锻炼质量和… |
| X1:6 | Others | 1 | Art. 8, Organization and implementation (八、组织实施) | 本意见自2023年9月8日起实施,有效期至2028年9月8日。 |
| X2:1 | Short-term | 0 | — | — |
| X2:2 | Medium-term | 0 | — | — |
| X2:3 | Long-term | 0 | — | — |
| X2:4 | Within current year | 0 | — | — |
| X3:1 | Qualification requirements | 1 | Art. 5, Qualification requirements (五、任职条件) | 五、任职条件 学校教练员应具备以下条件: |
| X3:2 | Scope of application | 1 | Art. 2, Scope of application (二、适用范围) | 二、适用范围 本意见适用于中小学、中等职业学校(含技工院校,下同)和普通高校。 |
| X3:3 | Professional title system | 1 | Art. 7, Support measures (七、保障措施) | (三)学校教练员的职称层级、岗位等级和评价标准按照《人力资源社会保障部 体育总局关于深化体育专业人员职称制度改革的指导意见》(人社部发〔2020〕76号)有关规定执行。 |
| X3:4 | Job responsibilities | 1 | Art. 4, Duties of the post (四、岗位职责) | 四、岗位职责 学校教练员应服从学校工作安排,按照学校工作计划,发挥专业特长,参与学校体育工作。 |
| X3:5 | Implementation measures | 1 | Art. 6, Appointment to posts (六、岗位聘用) | 六、岗位聘用 (一)学校按规定程序要求制定学校教练员岗位设置实施方案,根据按需设岗、公开招聘、择优聘用的原则,开展岗位聘用工作。 |
| X4:1 | National | 0 | — | — |
| X4:2 | Provincial | 1 | Issuing authority / signature block (发文机关/落款) | 江苏省体育局 中共江苏省委机构编制委员会办公室 江苏省教育厅 江苏省人力资源和社会保障厅 苏体规〔2023〕2号 |
| X4:3 | Sub-provincial or affiliated | 0 | — | — |
| X5:1 | Strengthening | 1 | Title / transmittal notice (标题/发文通知) | 为深入贯彻落实《体育总局 中央编办 教育部 人力资源社会保障部关于在学校设置教练员岗位的实施意见》 (体人规字〔2023〕3号)精神,进一步深化体教融合,加强学校体育工作,建立和完善优秀体育后备人才培养体系,现就在学校设置教练员岗位提出… |
| X5:2 | Guiding | 0 | — | — |
| X5:3 | Encouraging | 1 | Art. 3, Establishment of posts (三、岗位设置) | 鼓励各级各类学校通过购买服务方式,与相关专业机构等社会力量合作,缓解体育师资不足的问题。 |
| X5:4 | Improving | 1 | Title / transmittal notice (标题/发文通知) | 为深入贯彻落实《体育总局 中央编办 教育部 人力资源社会保障部关于在学校设置教练员岗位的实施意见》 (体人规字〔2023〕3号)精神,进一步深化体教融合,加强学校体育工作,建立和完善优秀体育后备人才培养体系,现就在学校设置教练员岗位提出… |
| X5:5 | Carrying out | 1 | Art. 6, Appointment to posts (六、岗位聘用) | (一)学校按规定程序要求制定学校教练员岗位设置实施方案,根据按需设岗、公开招聘、择优聘用的原则,开展岗位聘用工作。 |
| X5:6 | Promoting | 0 | — | — |
| X6:1 | Government | 1 | Art. 6, Appointment to posts (六、岗位聘用) | (三)体育部门负责做好退役运动员转型学校教练员相关培训工作,教育部门在学校教练员入职后加强思想政治、职业道德和教学培训。 |
| X6:2 | Schools | 1 | Title / transmittal notice (标题/发文通知) | 省体育局 省委编办 省教育厅 省人力资源社会保障厅关于印发《关于在学校设置教练员岗位的实施意见》的通知 |
| X6:3 | Society | 1 | Art. 3, Establishment of posts (三、岗位设置) | 鼓励各级各类学校通过购买服务方式,与相关专业机构等社会力量合作,缓解体育师资不足的问题。 |
| X7:1 | Supervision and inspection | 1 | Art. 6(2), Organization and implementation (六、组织实施（二）) | 学校体育教练员队伍建设应纳入体育传统特色学校建设评选指标。 |
| X7:2 | Organizational leadership | 1 | Art. 8, Organization and implementation (八、组织实施) | 各级机构编制、教育、人力资源社会保障、体育等部门要提高认识,凝聚共识,分工协作,共同支持保障,形成推进合力。 |
| X7:3 | Publicity and guidance | 0 | — | — |
| X7:4 | Talent support | 1 | Art. 3, Establishment of posts (三、岗位设置) | 设立专职教练员岗位的学校,在核定的编制和专业技术岗位总量及结构比例内设置,专岗专用,纳入专业技术岗位进行管理。鼓励各级各类学校通过购买服务方式,与相关专业机构等社会力量合作,缓解体育师资不足的问题。 |
| X7:5 | Others | 0 | — | — |
| X8:1 | Clear objectives | 0 | — | — |
| X8:2 | Sufficient basis | 1 | Art. 3, Establishment of posts (三、岗位设置) | 学校可根据工作实际,设立专(兼)职教练员岗位。 |
| X8:3 | Scientific plan | 0 | — | — |
| X8:4 | Detailed content | 1 | Art. 5, Qualification requirements (五、任职条件) | 学校教练员应具备以下条件: (一)具有良好的思想政治素质和道德品质,遵纪守法,遵守职业道德规范,身心健康,举止文明。(二)热爱教育事业,为人师表,关爱学生,遵循教育规律和学生成长规律。(三)热爱体育事业,熟练掌握相应运动项目的竞赛规程及裁判规则,熟悉相应年龄段学生的运动生理、心理特点。(四)中小学、中等职业学校的教练员,应取得大学本科及以上学历和一级及以上运动员技术等级。 |
| X9:1 | Education | 1 | Title / transmittal notice (标题/发文通知) | 省体育局 省委编办 省教育厅 省人力资源社会保障厅关于印发《关于在学校设置教练员岗位的实施意见》的通知 |
| X9:2 | Sports | 1 | Title / transmittal notice (标题/发文通知) | 江苏省体育局 |

First-level values: X1 0.83; X2 0.00; X3 1.00; X4 0.33; X5 0.67; X6 1.00; X7 0.60; X8 0.50; X9 1.00. PMC index 5.93; concavity index 3.07.

## **P8 — Zhejiang Province**

| **Indicator** | **Meaning** | **Code** | **Locator** | **Quoted warrant** |
| --- | --- | --- | --- | --- |
| X1:1 | Forecasting | 0 | — | — |
| X1:2 | Regulation | 1 | Art. 2, Duties and qualification requirements (二、岗位职责与任职条件) | 任职学校体育教练员应具备大学本科以上学历,小学体育教练员学历要求可放宽到大学专科以上,且须经省级学校体育教练员任职岗前培训考核合格。 |
| X1:3 | Recommendation | 1 | Art. 1, Scope of and requirements for establishment (一、岗位设置范围与要求) | 义务教育阶段学生数600人以上的公办学校、普通高中、中等职业学校、高等院校根据学校体育工作需要,原则上设置1个以上专兼职体育教练员岗位,鼓励民办学校从实际出发设置体育教练员岗位。 |
| X1:4 | Description | 1 | Title / transmittal notice (标题/发文通知) | 为贯彻落实中共中央办公厅、国务院办公厅《关于全面加强和改进新时代学校体育工作的意见》(中办发〔2020〕36号),体育总局、教育部《关于深化体教融合促进青少年健康发展的意见》(体发〔2020〕1号)以及浙江省委办公厅、省政府办公厅《关于… |
| X1:5 | Guidance | 1 | Art. 5, Strengthening organization and implementation (五、加强组织实施) | 各市、县(市、区)要坚持“健康第一”的教育理念,通过学校设立教练员岗位加强学校体育工作,推动青少年文化学习和体育锻炼协调发展。 |
| X1:6 | Others | 1 | Art. 5, Strengthening organization and implementation (五、加强组织实施) | (十五)本意见自2022年12月1日起实施,由省体育局、省教育厅会同有关部门负责解释。 |
| X2:1 | Short-term | 0 | — | — |
| X2:2 | Medium-term | 1 | Art. 5(13), Strengthening organizational leadership (五、加强组织实施（十三）加强组织领导) | 各级编制、教育、人力社保、财政、体育等部门要加强组织领导,按照部门职责分工协作、联合督导,确保“十四五”期间学校体育教练员岗位设置和人员配备逐步到位。 |
| X2:3 | Long-term | 0 | — | — |
| X2:4 | Within current year | 0 | — | — |
| X3:1 | Qualification requirements | 1 | Art. 2, Duties and qualification requirements (二、岗位职责与任职条件) | 二、岗位职责与任职条件 (三)岗位职责。学校体育教练员主要承担学生体育运动专项技能训练,传授运动损伤防护康复等知识技能;承担学校运动队训练管理、开展运动训练、参加体育竞赛、选拔输送优秀体育后备人才、培养高水平运动员;指导组织各级各类体育… |
| X3:2 | Scope of application | 0 | — | — |
| X3:3 | Professional title system | 1 | Art. 3, Post management (三、岗位管理) | 学校体育教练员职称层级分别与事业单位专业技术岗位等级相对应,正高级对应专业技术岗位一至四级,副高级对应专业技术岗位五至七级,中级对应专业技术岗位八至十级,初级对应专业技术岗位十一至十三级。 |
| X3:4 | Job responsibilities | 1 | Art. 2, Duties and qualification requirements (二、岗位职责与任职条件) | 学校体育教练员主要承担学生体育运动专项技能训练,传授运动损伤防护康复等知识技能;承担学校运动队训练管理、开展运动训练、参加体育竞赛、选拔输送优秀体育后备人才、培养高水平运动员;指导组织各级各类体育赛事、校园体育社团活动、青少年体育俱乐部… |
| X3:5 | Implementation measures | 0 | — | — |
| X4:1 | National | 0 | — | — |
| X4:2 | Provincial | 1 | Issuing authority / signature block (发文机关/落款) | 中共浙江省委机构编制委员会办公室 浙江省教育厅 浙江省人力资源和社会保障厅 浙江省财政厅 浙江省体育局 浙体人〔2022〕275号 |
| X4:3 | Sub-provincial or affiliated | 0 | — | — |
| X5:1 | Strengthening | 1 | Title / transmittal notice (标题/发文通知) | 为贯彻落实中共中央办公厅、国务院办公厅《关于全面加强和改进新时代学校体育工作的意见》(中办发〔2020〕36号),体育总局、教育部《关于深化体教融合促进青少年健康发展的意见》(体发〔2020〕1号)以及浙江省委办公厅、省政府办公厅《关于… |
| X5:2 | Guiding | 1 | Art. 4(10), Opening career development pathways for school sports coaches (四、强化措施保障（十）畅通学校体育教练员队伍职业发展通道) | 建立各级各类体校与大中小学校合作机制,具备条件的体校可设立学校教练员培训基地,或组织体校教练员到大中小学校开展业务指导。 |
| X5:3 | Encouraging | 1 | Art. 1, Scope of and requirements for establishment (一、岗位设置范围与要求) | 义务教育阶段学生数600人以上的公办学校、普通高中、中等职业学校、高等院校根据学校体育工作需要,原则上设置1个以上专兼职体育教练员岗位,鼓励民办学校从实际出发设置体育教练员岗位。 |
| X5:4 | Improving | 1 | Art. 4, Strengthening support measures (四、强化措施保障) | 逐步完善中小学校“健康知识+基本运动技能+专项运动技能”学校体育教学模式,让每位学生掌握2项以上运动技能。 |
| X5:5 | Carrying out | 1 | Art. 2, Duties and qualification requirements (二、岗位职责与任职条件) | 承担学校运动队训练管理、开展运动训练、参加体育竞赛、选拔输送优秀体育后备人才、培养高水平运动员;指导组织各级各类体育赛事、校园体育社团活动、青少年体育俱乐部建设等工作。 |
| X5:6 | Promoting | 1 | Title / transmittal notice (标题/发文通知) | 为贯彻落实中共中央办公厅、国务院办公厅《关于全面加强和改进新时代学校体育工作的意见》(中办发〔2020〕36号),体育总局、教育部《关于深化体教融合促进青少年健康发展的意见》(体发〔2020〕1号)以及浙江省委办公厅、省政府办公厅《关于… |
| X6:1 | Government | 1 | Art. 4, Strengthening support measures (四、强化措施保障) | 统筹安排全省青少年体育赛事,体育、教育部门共同制订年度竞赛计划,统一注册资格,分别组织实施各项赛事活动,建立健全分学段(小学、初中、高中、大学)的四级青少年体育赛事体系,完善省、市、县三级竞赛制度。 |
| X6:2 | Schools | 1 | Title / transmittal notice (标题/发文通知) | 中共浙江省委机构编制委员会办公室等5部门关于印发《浙江省大中小学校体育教练员专业技术岗位设置管理实施意见》的通知 |
| X6:3 | Society | 1 | Art. 4, Strengthening support measures (四、强化措施保障) | 探索采取政府购买服务等方式,支持相关专业机构等社会力量提供体育训练、竞赛活动组织等服务,缓解学校体育教练员力量不足问题。 |
| X7:1 | Supervision and inspection | 1 | Art. 5, Strengthening organization and implementation (五、加强组织实施) | 各级编制、教育、人力社保、财政、体育等部门要加强组织领导,按照部门职责分工协作、联合督导,确保“十四五”期间学校体育教练员岗位设置和人员配备逐步到位。 |
| X7:2 | Organizational leadership | 1 | Art. 5, Strengthening organization and implementation (五、加强组织实施) | (十三)加强组织领导。 |
| X7:3 | Publicity and guidance | 1 | Art. 4, Strengthening support measures (四、强化措施保障) | 各级政府要优化教育支出结构,完善投入机制,鼓励引导社会资本支持学校体育发展,切实保障学校体育教练员工作开展的必要支出。 |
| X7:4 | Talent support | 1 | Art. 4, Strengthening support measures (四、强化措施保障) | 各级编制、教育、人力社保、财政、体育等部门要加强组织领导,按照部门职责分工协作、联合督导,确保“十四五”期间学校体育教练员岗位设置和人员配备逐步到位。 |
| X7:5 | Others | 1 | Art. 5, Strengthening organization and implementation (五、加强组织实施) | (十五)本意见自2022年12月1日起实施,由省体育局、省教育厅会同有关部门负责解释。 |
| X8:1 | Clear objectives | 1 | Art. 4(7), Building platforms for the work of school sports coaches (四、强化措施保障（七）积极搭建学校体育教练员作用发挥平台) | 逐步完善中小学校“健康知识+基本运动技能+专项运动技能”学校体育教学模式,让每位学生掌握2项以上运动技能。 |
| X8:2 | Sufficient basis | 1 | Art. 1, Scope of and requirements for establishment (一、岗位设置范围与要求) | 义务教育阶段学生数600人以上的公办学校、普通高中、中等职业学校、高等院校根据学校体育工作需要,原则上设置1个以上专兼职体育教练员岗位,鼓励民办学校从实际出发设置体育教练员岗位。 |
| X8:3 | Scientific plan | 1 | Art. 5(13), Strengthening organizational leadership (五、加强组织实施（十三）加强组织领导) | 各级编制、教育、人力社保、财政、体育等部门要加强组织领导,按照部门职责分工协作、联合督导,确保“十四五”期间学校体育教练员岗位设置和人员配备逐步到位。 |
| X8:4 | Detailed content | 1 | Art. 2, Duties and qualification requirements (二、岗位职责与任职条件) | 二、岗位职责与任职条件 (三)岗位职责。学校体育教练员主要承担学生体育运动专项技能训练,传授运动损伤防护康复等知识技能;承担学校运动队训练管理、开展运动训练、参加体育竞赛、选拔输送优秀体育后备人才、培养高水平运动员;指导组织各级各类体育… |
| X9:1 | Education | 1 | Title / transmittal notice (标题/发文通知) | 各市党委编办、教育局、人力资源和社会保障局、财政局、体育部门,省有关单位: |
| X9:2 | Sports | 1 | Title / transmittal notice (标题/发文通知) | 省体育局 |

First-level values: X1 0.83; X2 0.25; X3 0.60; X4 0.33; X5 1.00; X6 1.00; X7 1.00; X8 1.00; X9 1.00. PMC index 7.02; concavity index 1.98.

## **P9 — Jiangxi Province**

| **Indicator** | **Meaning** | **Code** | **Locator** | **Quoted warrant** |
| --- | --- | --- | --- | --- |
| X1:1 | Forecasting | 0 | — | — |
| X1:2 | Regulation | 1 | Art. 3, Principles of and requirements for establishment (三、岗位设置原则与要求) | 5.各地人力资源和社会保障部门应在专业技术岗位总量及结构比例方面给予支持。 |
| X1:3 | Recommendation | 1 | Art. 6, Support measures (六、保障措施) | 1.鼓励各级体校教练员兼任学校体育教练员、担任校外体育辅导员,定期进校园带训运动队、参与教师培训、组织课外体育活动、指导体育社团活动,为学生提供专项运动技能培训服务,并可按规定领取报酬。 |
| X1:4 | Description | 1 | Title / transmittal notice (标题/发文通知) | 为进一步深化体教融合,加强学校体育工作,完善江西体育后备人才培养体系,有效缓解各级各类学校体育专业人才不足问题,拓宽江西优秀退役运动员职业转型发展通道,根据中共中央办公厅、国务院办公厅《关于全面加强和改进新时代学校体育工作的意见》(中办… |
| X1:5 | Guidance | 1 | Art. 1, Guiding principles (一、指导思想) | 一、指导思想 以习近平新时代中国特色社会主义思想为指导,全面贯彻党的教育方针,以服务学生全面发展、增强综合素质为目标,坚持健康第一的教育理念,通过设置学校体育教练员岗位加强学校体育工作力量,提升青少年体育锻炼水平,帮助青少年增强体质、健… |
| X1:6 | Others | 1 | Art. 7, Organization and implementation (七、组织实施) | (四)本意见自印发之日起实施,由省体育局、省委编办、省教育厅、省人力资源和社会保障厅按照职能职责负责解释。 |
| X2:1 | Short-term | 0 | — | — |
| X2:2 | Medium-term | 0 | — | — |
| X2:3 | Long-term | 0 | — | — |
| X2:4 | Within current year | 0 | — | — |
| X3:1 | Qualification requirements | 1 | Art. 4, Duties and qualification requirements (四、岗位职责与任职条件) | 四、岗位职责与任职条件 (一)岗位职责 |
| X3:2 | Scope of application | 1 | Art. 2, Scope of application (二、适用范围) | 二、适用范围 本意见适用于义务教育阶段学校、普通高中、职业院校、普通高校。 |
| X3:3 | Professional title system | 1 | Art. 6, Support measures (六、保障措施) | 学校体育教练员的职称层级、岗位等级和评价标准按照《人力资源社会保障部 体育总局关于深化体育专业人员职称制度改革的指导意见》(人社部发〔2020〕76号)和《体育总局办公厅关于贯彻〈关于深化体育专业人员职称制度改革的指导意见〉有关问题的通… |
| X3:4 | Job responsibilities | 1 | Art. 4, Duties and qualification requirements (四、岗位职责与任职条件) | (一)岗位职责 学校体育教练员按照学校体育工作计划,发挥专业特长,参与体育教学和训练工作。 |
| X3:5 | Implementation measures | 1 | Art. 5(1), Principles of appointment (五、岗位聘用（一）聘用原则) | 学校在岗位总量和结构比例内,按照学校岗位设置实施方案,采取按需设岗、面向社会公开招聘、择优聘用的原则,开展体育教练员岗位聘用工作。聘用应注重思想道德品质和运动训练实绩考评。 |
| X4:1 | National | 0 | — | — |
| X4:2 | Provincial | 1 | Art. (4), Authority for interpretation ((四)解释机关) | (四)本意见自印发之日起实施,由省体育局、省委编办、省教育厅、省人力资源和社会保障厅按照职能职责负责解释。 |
| X4:3 | Sub-provincial or affiliated | 0 | — | — |
| X5:1 | Strengthening | 1 | Title / transmittal notice (标题/发文通知) | 为进一步深化体教融合,加强学校体育工作,完善江西体育后备人才培养体系,有效缓解各级各类学校体育专业人才不足问题,拓宽江西优秀退役运动员职业转型发展通道,根据中共中央办公厅、国务院办公厅《关于全面加强和改进新时代学校体育工作的意见》(中办… |
| X5:2 | Guiding | 1 | Art. 7(1), Strengthening leadership and division of responsibilities (七、组织实施（一）加强领导,分工协作) | 人力资源和社会保障部门负责指导学校体育教练员岗位设置、发布学校体育教练员岗位职称申报条件以及指导行业主管部门组织开展职称评审等相关工作。 |
| X5:3 | Encouraging | 1 | Art. 6, Support measures (六、保障措施) | 1.鼓励各级体校教练员兼任学校体育教练员、担任校外体育辅导员,定期进校园带训运动队、参与教师培训、组织课外体育活动、指导体育社团活动,为学生提供专项运动技能培训服务,并可按规定领取报酬。 |
| X5:4 | Improving | 1 | Title / transmittal notice (标题/发文通知) | 为进一步深化体教融合,加强学校体育工作,完善江西体育后备人才培养体系,有效缓解各级各类学校体育专业人才不足问题,拓宽江西优秀退役运动员职业转型发展通道,根据中共中央办公厅、国务院办公厅《关于全面加强和改进新时代学校体育工作的意见》(中办… |
| X5:5 | Carrying out | 1 | Art. 5, Appointment to posts (五、岗位聘用) | 学校在岗位总量和结构比例内,按照学校岗位设置实施方案,采取按需设岗、面向社会公开招聘、择优聘用的原则,开展体育教练员岗位聘用工作。 |
| X5:6 | Promoting | 1 | Title / transmittal notice (标题/发文通知) | 为进一步深化体教融合,加强学校体育工作,完善江西体育后备人才培养体系,有效缓解各级各类学校体育专业人才不足问题,拓宽江西优秀退役运动员职业转型发展通道,根据中共中央办公厅、国务院办公厅《关于全面加强和改进新时代学校体育工作的意见》(中办… |
| X6:1 | Government | 1 | Art. 7, Organization and implementation (七、组织实施) | 体育部门负责收集符合学校体育教练员岗位条件人员信息,做好退役运动员任学校体育教练员暨转换过渡期培训和资格认定工作,牵头组织职业供需见面会,拟定各级学校体育教练员岗位职称申报条件以及开展职称评审等相关工作。 |
| X6:2 | Schools | 1 | Title / transmittal notice (标题/发文通知) | 江西省学校设置体育教练员专业技术岗位的实施意见 / 南昌市体育局 |
| X6:3 | Society | 1 | Art. 3, Principles of and requirements for establishment (三、岗位设置原则与要求) | 3.有条件的地区可通过购买服务方式,与相关专业机构等社会力量合作,向学校提供体育教育教学服务,缓解体育师资不足问题。 |
| X7:1 | Supervision and inspection | 1 | Art. 6, Support measures (六、保障措施) | 学校体育教练员队伍建设应纳入体育传统特色学校、体育后备人才基地建设评选指标,对未按规定配置学校体育教练员岗位的,视情况取消相应资格。 |
| X7:2 | Organizational leadership | 1 | Art. 7(1), Strengthening leadership and division of responsibilities (七、组织实施（一）加强领导,分工协作) | 体育部门负责收集符合学校体育教练员岗位条件人员信息,做好退役运动员任学校体育教练员暨转换过渡期培训和资格认定工作,牵头组织职业供需见面会,拟定各级学校体育教练员岗位职称申报条件以及开展职称评审等相关工作。教育部门负责征集各级学校体育教练员岗位需求,开展学校体育教练员入职后的思想政治、职业道德和教学培训等相关工作。人力资源和社会保障部门负责指导学校体育教练员岗位设置、发布学校体育教练员岗位职称申报条件以及指导行业主管部门组织开展职称评审等相关工作。 |
| X7:3 | Publicity and guidance | 0 | — | — |
| X7:4 | Talent support | 1 | Art. 3, Principles of and requirements for establishment (三、岗位设置原则与要求) | 2.设立专职体育教练员岗位的学校,在核定的编制和专业技术岗位总量及结构比例内设置,专岗专用,纳入专业技术岗位进行管理。 |
| X7:5 | Others | 1 | Art. 7, Organization and implementation (七、组织实施) | (四)本意见自印发之日起实施,由省体育局、省委编办、省教育厅、省人力资源和社会保障厅按照职能职责负责解释。 |
| X8:1 | Clear objectives | 0 | — | — |
| X8:2 | Sufficient basis | 1 | Title / transmittal notice (标题/发文通知) | 为进一步深化体教融合,加强学校体育工作,完善江西体育后备人才培养体系,有效缓解各级各类学校体育专业人才不足问题,拓宽江西优秀退役运动员职业转型发展通道,根据中共中央办公厅、国务院办公厅《关于全面加强和改进新时代学校体育工作的意见》(中办… |
| X8:3 | Scientific plan | 0 | — | — |
| X8:4 | Detailed content | 1 | Art. 4, Duties and qualification requirements (四、岗位职责与任职条件) | 四、岗位职责与任职条件 (一)岗位职责 学校体育教练员按照学校体育工作计划,发挥专业特长,参与体育教学和训练工作。主要承担学生体育运动专项技能传授、体能训练和体育后备人才选育工作,承担学校体育赛事活动组织、学校运动队训练竞赛管理、运动损… |
| X9:1 | Education | 1 | Title / transmittal notice (标题/发文通知) | 为进一步深化体教融合,加强学校体育工作,完善江西体育后备人才培养体系,有效缓解各级各类学校体育专业人才不足问题,拓宽江西优秀退役运动员职业转型发展通道,根据中共中央办公厅、国务院办公厅《关于全面加强和改进新时代学校体育工作的意见》(中办… |
| X9:2 | Sports | 1 | Title / transmittal notice (标题/发文通知) | 江西省学校设置体育教练员专业技术岗位的实施意见 / 南昌市体育局 |

First-level values: X1 0.83; X2 0.00; X3 1.00; X4 0.33; X5 1.00; X6 1.00; X7 0.80; X8 0.50; X9 1.00. PMC index 6.47; concavity index 2.53.

## **P12 — Henan Province**

| **Indicator** | **Meaning** | **Code** | **Locator** | **Quoted warrant** |
| --- | --- | --- | --- | --- |
| X1:1 | Forecasting | 0 | — | — |
| X1:2 | Regulation | 1 | Art. 1, Scope of and requirements for establishment (一、岗位设置范围与要求) | 各地人力资源社会保障部门应在专业技术岗位总量及结构比例方面给予支持。 |
| X1:3 | Recommendation | 1 | Art. 1, Scope of and requirements for establishment (一、岗位设置范围与要求) | 全省义务教育阶段学校、普通高中、职业院校、普通高校可根据工作实际,设立专(兼)职学校教练员岗位。 |
| X1:4 | Description | 1 | Title / transmittal notice (标题/发文通知) | 为贯彻落实中共中央办公厅、国务院办公厅《关于全面加强和改进新时代学校体育工作的意见》(中办发〔2020〕36号),体育总局、教育部《关于深化体教融合促进青少年健康发展的意见》(体发〔2020〕1号)以及体育总局、中央编办、教育部、人力资… |
| X1:5 | Guidance | 0 | — | — |
| X1:6 | Others | 0 | — | — |
| X2:1 | Short-term | 0 | — | — |
| X2:2 | Medium-term | 0 | — | — |
| X2:3 | Long-term | 0 | — | — |
| X2:4 | Within current year | 0 | — | — |
| X3:1 | Qualification requirements | 1 | Art. 4, Qualification requirements (四、任职条件) | 四、任职条件 学校教练员应具备良好的思想政治素质和道德品质,遵纪守法,遵守职业道德规范,身心健康、举止文明;热爱教育事业,为人师表,关爱学生,遵循教育规律和学生成长规律;热爱体育事业,了解相应运动项目的竞赛规程及裁判规则,熟悉相应年龄段… |
| X3:2 | Scope of application | 0 | — | — |
| X3:3 | Professional title system | 1 | Art. 3, Professional title system (三、职称体系) | 三、职称体系 学校教练员的职称层级、岗位等级和评价标准按照《人力资源社会保障部 体育总局关于深化体育专业人员职称制度改革的指导意见》(人社部发〔2020〕76号)有关规定执行。 |
| X3:4 | Job responsibilities | 1 | Art. 2, Duties of the post (二、岗位职责) | 二、岗位职责 学校教练员按照学校体育工作计划,发挥专业特长,参与体育教学和训练工作。 |
| X3:5 | Implementation measures | 1 | Art. 5, Appointment to and management of posts (五、岗位聘用及管理) | (一)学校根据公开招聘、择优聘用的原则,按照规定程序要求组织开展学校教练员岗位聘用工作。 |
| X4:1 | National | 0 | — | — |
| X4:2 | Provincial | 1 | Issuing authority / signature block (发文机关/落款) | 河南省体育局 中共河南省委编制委员会办公室 河南省教育厅 河南省人力资源和社会保障厅 |
| X4:3 | Sub-provincial or affiliated | 0 | — | — |
| X5:1 | Strengthening | 1 | Title / transmittal notice (标题/发文通知) | 为贯彻落实中共中央办公厅、国务院办公厅《关于全面加强和改进新时代学校体育工作的意见》(中办发〔2020〕36号),体育总局、教育部《关于深化体教融合促进青少年健康发展的意见》(体发〔2020〕1号)以及体育总局、中央编办、教育部、人力资… |
| X5:2 | Guiding | 0 | — | — |
| X5:3 | Encouraging | 0 | — | — |
| X5:4 | Improving | 1 | Art. 6, Support measures (六、保障措施) | (二)各地要统筹利用学校教练员资源,探索完善学校教练员多点执教、跨校执教机制,合理规划配置“三大球”、游泳及田径等基础大项、冰雪运动等各类运动项目学校教练员,按区域统筹安排学校教练员的教学训练任务,最大限度发挥学校教练员作用。 |
| X5:5 | Carrying out | 1 | Art. 5, Appointment to and management of posts (五、岗位聘用及管理) | (一)学校根据公开招聘、择优聘用的原则,按照规定程序要求组织开展学校教练员岗位聘用工作。 |
| X5:6 | Promoting | 1 | Title / transmittal notice (标题/发文通知) | 为贯彻落实中共中央办公厅、国务院办公厅《关于全面加强和改进新时代学校体育工作的意见》(中办发〔2020〕36号),体育总局、教育部《关于深化体教融合促进青少年健康发展的意见》(体发〔2020〕1号)以及体育总局、中央编办、教育部、人力资… |
| X6:1 | Government | 1 | Art. 6, Support measures (六、保障措施) | 体育部门负责做好退役运动员转型学校教练员培训工作,教育部门和人力资源社会保障部门在学校教练员入职后加强思想政治、职业道德和教学培训,提高教育教学能力水平。 |
| X6:2 | Schools | 1 | Title / transmittal notice (标题/发文通知) | 河南省大中小学校教练员岗位设置管理的实施意见 |
| X6:3 | Society | 1 | Art. 1, Scope of and requirements for establishment (一、岗位设置范围与要求) | 有条件的地区可以通过购买服务方式,与相关专业机构等社会力量合作向学校提供体育教育教学服务,缓解体育师资不足问题。 |
| X7:1 | Supervision and inspection | 1 | Art. 7, Organization and implementation (七、组织实施) | (二)各地可依据本意见制定实施细则,先行先试、统筹推进,不断完善学校体育工作机制和考核评价体系。 |
| X7:2 | Organizational leadership | 1 | Art. 7, Organization and implementation (七、组织实施) | 各地要高度重视,加强组织领导。 |
| X7:3 | Publicity and guidance | 0 | — | — |
| X7:4 | Talent support | 1 | Art. 1, Scope of and requirements for establishment (一、岗位设置范围与要求) | 设立专职学校教练员岗位的学校,在核定的编制和专业技术岗位总量及结构比例内设置,纳入岗位设置方案,专岗专用,并按照专业技术岗位进行管理。 |
| X7:5 | Others | 0 | — | — |
| X8:1 | Clear objectives | 0 | — | — |
| X8:2 | Sufficient basis | 1 | Art. 1, Scope of and requirements for establishment (一、岗位设置范围与要求) | 全省义务教育阶段学校、普通高中、职业院校、普通高校可根据工作实际,设立专(兼)职学校教练员岗位。 |
| X8:3 | Scientific plan | 1 | Art. 1, Scope of and requirements for establishment (一、岗位设置范围与要求) | 一、岗位设置范围与要求 全省义务教育阶段学校、普通高中、职业院校、普通高校可根据工作实际,设立专(兼)职学校教练员岗位。 |
| X8:4 | Detailed content | 1 | Art. 4, Qualification requirements (四、任职条件) | 四、任职条件 学校教练员应具备良好的思想政治素质和道德品质,遵纪守法,遵守职业道德规范,身心健康、举止文明;热爱教育事业,为人师表,关爱学生,遵循教育规律和学生成长规律;热爱体育事业,了解相应运动项目的竞赛规程及裁判规则,熟悉相应年龄段… |
| X9:1 | Education | 1 | Title / transmittal notice (标题/发文通知) | 发布日期:2023-08-16 09:59信息来源: 局人事教育处 |
| X9:2 | Sports | 1 | Title / transmittal notice (标题/发文通知) | 各省辖市、济源示范区体育、教育行政部门、党委(党工委)编办、人力资源社会保障局,省有关单位: |

First-level values: X1 0.50; X2 0.00; X3 0.80; X4 0.33; X5 0.67; X6 1.00; X7 0.60; X8 0.75; X9 1.00. PMC index 5.65; concavity index 3.35.

## **P13 — Jilin Province**

| **Indicator** | **Meaning** | **Code** | **Locator** | **Quoted warrant** |
| --- | --- | --- | --- | --- |
| X1:1 | Forecasting | 0 | — | — |
| X1:2 | Regulation | 1 | Art. 4, Qualification requirements (四、任职条件) | (一)学校体育教练员应具备以下基本条件: |
| X1:3 | Recommendation | 1 | Art. 7, Support measures (七、保障措施) | 4.支持鼓励学校从事体育教学、体育训练人员同时具备学校体育教练员和体育教师职称,具备相应资质条件的可聘任到学校体育教练员或体育教师岗位工作。 |
| X1:4 | Description | 1 | Title / transmittal notice (标题/发文通知) | 为贯彻落实中共中央办公厅、国务院办公厅《关于全面加强和改进新时代学校体育工作的意见》(中办发〔2020〕36号),体育总局、教育部《关于印发深化体教融合促进青少年健康发展意见的通知》(体发〔2020〕1号)和体育总局、中央编办、教育部、… |
| X1:5 | Guidance | 1 | Art. 1, Guiding principles (一、指导思想) | 一、指导思想 以习近平新时代中国特色社会主义思想为指导,全面贯彻党的教育方针,以服务学生全面发展、增强综合素质为目标,坚持健康第一的教育理念,通过在学校工作的体育教练员(以下简称“学校体育教练员”)加强学校体育工作力量,提升青少年体育锻… |
| X1:6 | Others | 1 | Art. 7, Support measures (七、保障措施) | (五)本办法自发布之日起实施,由省体育局、省委编办、省教育厅、省人社厅按照职责负责解释。 |
| X2:1 | Short-term | 0 | — | — |
| X2:2 | Medium-term | 1 | Art. 7(1), Support measures (七、保障措施（一）) | 确保“十四五”期间学校体育教练员岗位设置和人员配备逐步到位。 |
| X2:3 | Long-term | 0 | — | — |
| X2:4 | Within current year | 0 | — | — |
| X3:1 | Qualification requirements | 1 | Art. 4, Qualification requirements (四、任职条件) | 四、任职条件 (一)学校体育教练员应具备以下基本条件: |
| X3:2 | Scope of application | 0 | — | — |
| X3:3 | Professional title system | 1 | Art. 6, Professional title system (六、职称体系) | 六、职称体系 学校体育教练员的职称层级、岗位等级和评价标准按照《人力资源社会保障部体育总局关于深化体育专业人员职称制度改革的指导意见》(人社部发〔2020〕76号)有关规定执行。 |
| X3:4 | Job responsibilities | 1 | Art. 3, Duties of the post (三、岗位职责) | 三、岗位职责 学校体育教练员按照学校体育工作计划,发挥专业特长,参与体育教学和训练工作。 |
| X3:5 | Implementation measures | 1 | Art. 5, Post management (五、岗位管理) | 中小学、业余体校、中等职业学校主管部门要按照有关文件规定,统筹制定学校体育教练员岗位设置方案,坚持按需设岗、公开招聘、择优聘用的原则,开展岗位聘用工作,将学校体育教练员纳入人事制度统一管理。 |
| X4:1 | National | 0 | — | — |
| X4:2 | Provincial | 1 | Issuing authority / signature block (发文机关/落款) | 吉林省体育局 中共吉林省委机构编制委员会办公室 吉林省教育厅 吉林省人力资源和社会保障厅 |
| X4:3 | Sub-provincial or affiliated | 0 | — | — |
| X5:1 | Strengthening | 1 | Title / transmittal notice (标题/发文通知) | 为贯彻落实中共中央办公厅、国务院办公厅《关于全面加强和改进新时代学校体育工作的意见》(中办发〔2020〕36号),体育总局、教育部《关于印发深化体教融合促进青少年健康发展意见的通知》(体发〔2020〕1号)和体育总局、中央编办、教育部、… |
| X5:2 | Guiding | 0 | — | — |
| X5:3 | Encouraging | 1 | Art. 7, Support measures (七、保障措施) | 4.支持鼓励学校从事体育教学、体育训练人员同时具备学校体育教练员和体育教师职称,具备相应资质条件的可聘任到学校体育教练员或体育教师岗位工作。 |
| X5:4 | Improving | 1 | Art. 7, Support measures (七、保障措施) | 四)各地可依据本办法研究制定实施细则,先行先试、统筹推进,不断完善学校体育工作机制和考核评价体系。 |
| X5:5 | Carrying out | 1 | Art. 5, Post management (五、岗位管理) | 中小学、业余体校、中等职业学校主管部门要按照有关文件规定,统筹制定学校体育教练员岗位设置方案,坚持按需设岗、公开招聘、择优聘用的原则,开展岗位聘用工作,将学校体育教练员纳入人事制度统一管理。 |
| X5:6 | Promoting | 1 | Title / transmittal notice (标题/发文通知) | 为贯彻落实中共中央办公厅、国务院办公厅《关于全面加强和改进新时代学校体育工作的意见》(中办发〔2020〕36号),体育总局、教育部《关于印发深化体教融合促进青少年健康发展意见的通知》(体发〔2020〕1号)和体育总局、中央编办、教育部、… |
| X6:1 | Government | 1 | Art. 5, Post management (五、岗位管理) | (二)体育部门负责做好退役运动员转型学校体育教练员培训工作,教育部门在学校体育教练员入职后加强思想政治、职业道德和教学培训,提升教育教学能力水平。 |
| X6:2 | Schools | 1 | Title / transmittal notice (标题/发文通知) | 关于印发《吉林省大中小学校体育教练员专业技术岗位设置管理实施办法》的通知 |
| X6:3 | Society | 0 | — | — |
| X7:1 | Supervision and inspection | 1 | Art. 7, Support measures (七、保障措施) | (一)学校体育教练员人才队伍建设,事关全省青少年身心健康全面发展和体育优秀后备人才培养成效,各级机构编制、教育、人力资源和社会保障、体育等部门要加强组织领导,强化部门职责,分工协作、联合督导,及时研究解决工作中出现的各类情况和问题,机构… |
| X7:2 | Organizational leadership | 1 | Art. 7, Support measures (七、保障措施) | (一)学校体育教练员人才队伍建设,事关全省青少年身心健康全面发展和体育优秀后备人才培养成效,各级机构编制、教育、人力资源和社会保障、体育等部门要加强组织领导,强化部门职责,分工协作、联合督导,及时研究解决工作中出现的各类情况和问题,机构… |
| X7:3 | Publicity and guidance | 0 | — | — |
| X7:4 | Talent support | 1 | Art. 7(2), Strengthening the recruitment of sports personnel (七、保障措施（二）加大体育人才引进力度) | 各地要将高水平优秀运动员、体育教练员纳入本地人才认定范围,并享受相应的人才引进政策。 |
| X7:5 | Others | 1 | Art. 7, Support measures (七、保障措施) | (五)本办法自发布之日起实施,由省体育局、省委编办、省教育厅、省人社厅按照职责负责解释。 |
| X8:1 | Clear objectives | 1 | Art. 7(1), Support measures (七、保障措施（一）) | 确保“十四五”期间学校体育教练员岗位设置和人员配备逐步到位。 |
| X8:2 | Sufficient basis | 1 | Art. 2, Scope and number of posts (二、岗位设置范围与数量) | (二)公办义务教育学校每个校区、公办普通高中、业余体校、中等职业学校、高等院校根据学校体育工作需要,原则上至少设置1名体育教练员专业技术岗位。 |
| X8:3 | Scientific plan | 1 | Art. 7(1), Support measures (七、保障措施（一）) | 各级机构编制、教育、人力资源和社会保障、体育等部门要加强组织领导,强化部门职责,分工协作、联合督导,及时研究解决工作中出现的各类情况和问题,机构编制部门按规定做好编制保障工作,人力资源和社会保障部门在专业技术岗位总量及结构比例方面给予支持,体育部门负责做好退役运动员转型学校体育教练员培训工作,教育部门负责做好在学校体育教练员入职后加强思想政治、职业道德和教学培养等工作,确保“十四五”期间学校体育教练员岗位设置和人员配备逐步到位。 |
| X8:4 | Detailed content | 1 | Art. 4, Qualification requirements (四、任职条件) | 四、任职条件 (一)学校体育教练员应具备以下基本条件: 1.具有良好的思想政治素质和道德品质,遵纪守法,遵守职业道德规范,身心健康,举止文明。 2.热爱教育事业,为人师表,关爱学生,遵循教育规律和学生成长规律。 3.热爱体育事业,了解相… |
| X9:1 | Education | 1 | Title / transmittal notice (标题/发文通知) | 科技教育 |
| X9:2 | Sports | 1 | Title / transmittal notice (标题/发文通知) | 关于印发《吉林省大中小学校体育教练员专业技术岗位设置管理实施办法》的通知 |

First-level values: X1 0.83; X2 0.25; X3 0.80; X4 0.33; X5 0.83; X6 0.67; X7 0.80; X8 1.00; X9 1.00. PMC index 6.52; concavity index 2.48.

## **P15 — Inner Mongolia Autonomous Region**

| **Indicator** | **Meaning** | **Code** | **Locator** | **Quoted warrant** |
| --- | --- | --- | --- | --- |
| X1:1 | Forecasting | 0 | — | — |
| X1:2 | Regulation | 1 | Art. 2, Duties and qualification requirements (二、岗位职责和任职条件) | 任职学校体育教练员,应具备良好的思想政治素质和道德品质,遵纪守法,遵守职业道德规范,身心健康、举止文明;铸牢中华民族共同体意识,自觉维护民族团结进步;热爱教育事业,为人师表,关爱学生,遵循教育规律和学生成长规律; |
| X1:3 | Recommendation | 1 | Art. 4, Support measures (四、保障措施) | 鼓励各级体育运动学校(以下简称“体校”)、俱乐部中具备相应资质的体育教练员兼职担任学校体育教练员,并按规定领取报酬。 |
| X1:4 | Description | 1 | Title / transmittal notice (标题/发文通知) | 根据国家《关于全面加强和改进新时代学校体育工作的意见》(中办发〔2020〕36号)、《关于深化体教融合促进青少年健康发展的意见》(体发〔2020〕1号)、《关于在学校设置教练员岗位的实施意见》(体人规字〔2023〕3号)精神,为进一步深… |
| X1:5 | Guidance | 1 | Art. 5, Organization and implementation (五、组织实施) | 各地各校要深入学习贯彻习近平总书记关于体育的重要论述,坚持“健康第一”的教育理念,通过设立教练员岗位加强学校体育工作,推动青少年文化学习和体育锻炼协调发展,为完成好习近平总书记交给内蒙古的五大任务和全方位建设“模范自治区”两件大事作出积… |
| X1:6 | Others | 1 | Art. 5, Organization and implementation (五、组织实施) | 本意见自印发之日起实施,由自治区体育局、教育厅会同有关部门负责解释。 |
| X2:1 | Short-term | 0 | — | — |
| X2:2 | Medium-term | 1 | Art. 5(2), Strengthening organizational leadership (五、组织实施（二）加强组织领导) | 各级机构编制、教育、人力资源和社会保障、体育等部门要加强组织领导,细化职责分工,及时研究解决工作中出现的各类情况和问题,确保“十四五”期间学校体育教练员岗位设置和人员配备逐步到位。 |
| X2:3 | Long-term | 0 | — | — |
| X2:4 | Within current year | 0 | — | — |
| X3:1 | Qualification requirements | 1 | Art. 2, Duties and qualification requirements (二、岗位职责和任职条件) | 二、岗位职责和任职条件 (一)岗位职责。学校体育教练员主要承担学生体育运动专项技能、体能训练和优秀体育后备人才选育工作,承担学校运动队训练管理、运动损伤防护康复、训练疲劳恢复等知识技能传授,以及各级各类体育赛事组织、校园体育社团活动指导… |
| X3:2 | Scope of application | 0 | — | — |
| X3:3 | Professional title system | 1 | Art. 3(2), Professional title series (三、岗位管理（二）职称系列) | 学校体育教练员职称纳入体育专业技术人员职称系列。初级、中级、副高级、正高级职称的名称分别为初级教练员、中级教练员、高级教练员、国家级教练员,职称评价标准条件另行制定。执教期间,学生体质和运动能力提升情况、体育后备人才培养输送情况、学校体育赛事活动组织情况、学校体育社团管理情况等,均可作为其职称评审有效业绩。学校体育教练员职称层级分别与事业单位专业技术岗位等级相对应,正高级对应专业技术岗位一至四级,副高级对应专业技术岗位五至七级,中级对应专业技术岗位八至十级,初级对应专业技术岗位十一至十三级。 |
| X3:4 | Job responsibilities | 1 | Art. 2, Duties and qualification requirements (二、岗位职责和任职条件) | 学校体育教练员主要承担学生体育运动专项技能、体能训练和优秀体育后备人才选育工作,承担学校运动队训练管理、运动损伤防护康复、训练疲劳恢复等知识技能传授,以及各级各类体育赛事组织、校园体育社团活动指导、青少年体育俱乐部建设、优秀运动队共建等… |
| X3:5 | Implementation measures | 1 | Art. 4, Support measures (四、保障措施) | 学校教练员岗位优先面向取得一级及以上运动员技术等级的退役运动员和高校高水平运动队毕业生公开招聘。 |
| X4:1 | National | 0 | — | — |
| X4:2 | Provincial | 1 | Issuing authority / signature block (发文机关/落款) | 内蒙古自治区体育局 中共内蒙古自治区委员会机构编制委员会办公室 内蒙古自治区教育厅 内蒙古自治区人力资源和社会保障厅 |
| X4:3 | Sub-provincial or affiliated | 0 | — | — |
| X5:1 | Strengthening | 1 | Title / transmittal notice (标题/发文通知) | 根据国家《关于全面加强和改进新时代学校体育工作的意见》(中办发〔2020〕36号)、《关于深化体教融合促进青少年健康发展的意见》(体发〔2020〕1号)、《关于在学校设置教练员岗位的实施意见》(体人规字〔2023〕3号)精神,为进一步深… |
| X5:2 | Guiding | 1 | Art. 4(2), Opening development pathways for the coaching workforce (四、保障措施（二）畅通学校体育教练员队伍发展路径) | 建立体校与普通大中小学校合作机制,具备条件的体校可设立学校体育教练员培训基地,或组织体校教练员到普通大中小学校开展业务指导。 |
| X5:3 | Encouraging | 1 | Art. 4, Support measures (四、保障措施) | 鼓励各级体育运动学校(以下简称“体校”)、俱乐部中具备相应资质的体育教练员兼职担任学校体育教练员,并按规定领取报酬。 |
| X5:4 | Improving | 1 | Art. 4, Support measures (四、保障措施) | 完善体育教练员职称评聘标准,确保体育教练员在职务职称晋升、教学科研成果评定等方面,与各学科教师享受同等待遇。 |
| X5:5 | Carrying out | 1 | Art. 4, Support measures (四、保障措施) | 建立体校与普通大中小学校合作机制,具备条件的体校可设立学校体育教练员培训基地,或组织体校教练员到普通大中小学校开展业务指导。 |
| X5:6 | Promoting | 1 | Title / transmittal notice (标题/发文通知) | 根据国家《关于全面加强和改进新时代学校体育工作的意见》(中办发〔2020〕36号)、《关于深化体教融合促进青少年健康发展的意见》(体发〔2020〕1号)、《关于在学校设置教练员岗位的实施意见》(体人规字〔2023〕3号)精神,为进一步深… |
| X6:1 | Government | 1 | Art. 3, Post management (三、岗位管理) | 各级人力资源社会保障部门应在专业技术岗位设置方面给予支持。 |
| X6:2 | Schools | 1 | Title / transmittal notice (标题/发文通知) | 现将《关于在内蒙古自治区大中小学校设置体育教练员专业技术岗位的实施意见》印发给你们,请认真贯彻执行。 |
| X6:3 | Society | 1 | Art. 4, Support measures (四、保障措施) | 探索采取政府购买服务等方式,支持青少年体育俱乐部和体育校外培训机构等社会力量提供体育训练、竞赛活动组织等服务。 |
| X7:1 | Supervision and inspection | 1 | Art. 5, Organization and implementation (五、组织实施) | 各盟市可依据本意见制定实施细则,鼓励创建体教融合试点的盟市、旗县先行先试,不断完善学校体育工作机制和考核评价体系。 |
| X7:2 | Organizational leadership | 1 | Art. 5, Organization and implementation (五、组织实施) | (二)加强组织领导。 |
| X7:3 | Publicity and guidance | 1 | Art. 4, Support measures (四、保障措施) | 各级政府要优化教育支出结构,完善投入机制,鼓励引导社会资本支持学校体育发展,切实保障学校体育教练员工作开展的必要支出。 |
| X7:4 | Talent support | 1 | Art. 4(4), Strengthening support for the coaching workforce (四、保障措施（四）加大学校体育教练员队伍保障力度) | 聘用学校体育教练员所需编制原则上在聘用单位核定编制总量内统筹解决。各级政府要优化教育支出结构,完善投入机制,鼓励引导社会资本支持学校体育发展,切实保障学校体育教练员工作开展的必要支出。各地要结合实际制定兼职体育教练员享受待遇的相关政策,稳步提高兼职体育教练员待遇。 |
| X7:5 | Others | 1 | Art. 5, Organization and implementation (五、组织实施) | 本意见自印发之日起实施,由自治区体育局、教育厅会同有关部门负责解释。 |
| X8:1 | Clear objectives | 1 | Art. 5(2), Strengthening organizational leadership (五、组织实施（二）加强组织领导) | 确保“十四五”期间学校体育教练员岗位设置和人员配备逐步到位。 |
| X8:2 | Sufficient basis | 1 | Title / transmittal notice (标题/发文通知) | 根据国家《关于全面加强和改进新时代学校体育工作的意见》(中办发〔2020〕36号)、《关于深化体教融合促进青少年健康发展的意见》(体发〔2020〕1号)、《关于在学校设置教练员岗位的实施意见》(体人规字〔2023〕3号)精神,为进一步深… |
| X8:3 | Scientific plan | 1 | Art. 5(2), Strengthening organizational leadership (五、组织实施（二）加强组织领导) | 各级机构编制、教育、人力资源和社会保障、体育等部门要加强组织领导,细化职责分工,及时研究解决工作中出现的各类情况和问题,确保“十四五”期间学校体育教练员岗位设置和人员配备逐步到位。 |
| X8:4 | Detailed content | 1 | Art. 2, Duties and qualification requirements (二、岗位职责和任职条件) | 二、岗位职责和任职条件 (一)岗位职责。学校体育教练员主要承担学生体育运动专项技能、体能训练和优秀体育后备人才选育工作,承担学校运动队训练管理、运动损伤防护康复、训练疲劳恢复等知识技能传授,以及各级各类体育赛事组织、校园体育社团活动指导… |
| X9:1 | Education | 1 | Title / transmittal notice (标题/发文通知) | 各盟市党委编办,体育、教育行政主管部门、人社局,各高等院校: |
| X9:2 | Sports | 1 | Title / transmittal notice (标题/发文通知) | 各盟市党委编办,体育、教育行政主管部门、人社局,各高等院校: |

First-level values: X1 0.83; X2 0.25; X3 0.80; X4 0.33; X5 1.00; X6 1.00; X7 1.00; X8 1.00; X9 1.00. PMC index 7.22; concavity index 1.78.

## **P17 — Chongqing Municipality**

| **Indicator** | **Meaning** | **Code** | **Locator** | **Quoted warrant** |
| --- | --- | --- | --- | --- |
| X1:1 | Forecasting | 0 | — | — |
| X1:2 | Regulation | 1 | Art. 2, Implementation (二、工作实施) | 市级以上体育传统特色学校(体育传统项目学校、体育特色学校)、国家高水平体育后备人才基地学校、联办共建区(县)级以上高水平运动队学校、高等院校原则上应在核定的专业技术岗位中设置不少于1个专职教练员岗位。 |
| X1:3 | Recommendation | 1 | Art. 2, Implementation (二、工作实施) | 鼓励其他学校从实际出发设置专(兼)职教练员岗位。 |
| X1:4 | Description | 0 | — | — |
| X1:5 | Guidance | 1 | Art. 1, Guiding principles (一、指导思想) | 一、指导思想 以习近平新时代中国特色社会主义思想为指导,全面贯彻落实党的教育方针,围绕体教融合,在大中小学校设置体育教练员岗位(以下简称“教练员岗位”),完善体育师资队伍建设体系,加强学校体育工作力量,帮助青少年在体育锻炼中享受乐趣、增… |
| X1:6 | Others | 0 | — | — |
| X2:1 | Short-term | 0 | — | — |
| X2:2 | Medium-term | 0 | — | — |
| X2:3 | Long-term | 0 | — | — |
| X2:4 | Within current year | 0 | — | — |
| X3:1 | Qualification requirements | 1 | Art. 2, Implementation (二、工作实施) | 一般应具备大学本科及以上学历,中小学体育教练员学历要求可放宽到大学专科及以上招聘。 |
| X3:2 | Scope of application | 1 | Art. 3, Organizational support (三、组织保障) | 本实施方案适用于全市义务教育阶段学校、普通高中、职业院校、普通高校。 |
| X3:3 | Professional title system | 1 | Art. 2, Implementation (二、工作实施) | 学校教练员的职称层级、岗位等级和评价标准按照《人力资源社会保障部体育总局关于深化体育专业人员职称制度改革的指导意见》(人社部发〔2020〕76 号)及重庆市有关规定执行, 纳入我市体育教练员职称评审体系。 |
| X3:4 | Job responsibilities | 1 | Art. 2, Implementation (二、工作实施) | (二)岗位职责 教练员主要承担学生体育运动专项技能训练,传授运动损伤防护康复等知识技能;承担学校运动队训练管理、开展运动训练、参加体育竞赛、选拔输送优秀体育后备人才、培养高水平运动员;指导组织各级各类体育赛事、校园体育社团活动、青少年体… |
| X3:5 | Implementation measures | 1 | Art. 2, Implementation (二、工作实施) | (四)岗位聘用 1.学校在岗位总量和结构比例内,按照学校岗位设置实施方案,根据按需设岗、公开招聘、择优聘用的原则,开展教练员岗位聘用工作。 |
| X4:1 | National | 0 | — | — |
| X4:2 | Provincial | 1 | Art. 3(3), Strengthening supervision and inspection (三、(三)强化督导检查) | 市体育局、市委编办、市教委、市人力资源和社会保障局定期组织联合督查检查 |
| X4:3 | Sub-provincial or affiliated | 0 | — | — |
| X5:1 | Strengthening | 1 | Title / transmittal notice (标题/发文通知) | 为深入贯彻落实中央全面深化改革委员会第十三次会议审议通过的《关于深化体教融合 促进青少年健康发展的意见》(体发〔2020〕1 号)、《体育总局 中央编办 教育部 人力资源社会保障部关于在学校设置教练员岗位的实施意见》(体人规字〔2023… |
| X5:2 | Guiding | 1 | Art. 3(1), Organizational support (三、组织保障（一）) | 各区县体育、教育、人力资源社会保障部门负责共同指导学校制定培养和招聘计划,督促学校落实教练员岗位设置、聘用、职称评定等工作。 |
| X5:3 | Encouraging | 1 | Art. 2, Implementation (二、工作实施) | 鼓励其他学校从实际出发设置专(兼)职教练员岗位。 |
| X5:4 | Improving | 1 | Art. 1, Guiding principles (一、指导思想) | 以习近平新时代中国特色社会主义思想为指导,全面贯彻落实党的教育方针,围绕体教融合,在大中小学校设置体育教练员岗位(以下简称“教练员岗位”),完善体育师资队伍建设体系,加强学校体育工作力量,帮助青少年在体育锻炼中享受乐趣、增强体质、健全人… |
| X5:5 | Carrying out | 1 | Art. 2, Implementation (二、工作实施) | 承担学校运动队训练管理、开展运动训练、参加体育竞赛、选拔输送优秀体育后备人才、培养高水平运动员;指导组织各级各类体育赛事、校园体育社团活动、青少年体育俱乐部建设等工作。 |
| X5:6 | Promoting | 1 | Title / transmittal notice (标题/发文通知) | 为深入贯彻落实中央全面深化改革委员会第十三次会议审议通过的《关于深化体教融合 促进青少年健康发展的意见》(体发〔2020〕1 号)、《体育总局 中央编办 教育部 人力资源社会保障部关于在学校设置教练员岗位的实施意见》(体人规字〔2023… |
| X6:1 | Government | 1 | Art. 2, Implementation (二、工作实施) | 结合本市实际,依托国家人社部、教育部、国家体育总局转型学校教练员培训体系,对符合条件的教练员,且有意投身教育事业的,由市级体育、教育部门统筹组织,开展免费专项培训, 帮助其提升教育教学能力。 |
| X6:2 | Schools | 1 | Title / transmittal notice (标题/发文通知) | 重庆市大中小学校设置教练员岗位实施方案 |
| X6:3 | Society | 0 | — | — |
| X7:1 | Supervision and inspection | 1 | Art. 3(3), Strengthening supervision and inspection (三、组织保障（三）强化督导检查) | 市体育局、市委编办、市教委、市人力资源和社会保障局定期组织联合督查检查,将是否落实学校教练员岗位设置,岗位聘用等工作作为检查内容。检查结果可作为市级以上体育传统特色学校(体育传统项目学校、体育特色学校)、国家高水平体育后备人才基地学校、联办共建区(县)级以上高水平运动队学校申报、评审以及学校评优评先等方面的参考依据。 |
| X7:2 | Organizational leadership | 0 | — | — |
| X7:3 | Publicity and guidance | 0 | — | — |
| X7:4 | Talent support | 1 | Art. 2(6), Professional training (二、工作实施（六）专业培训) | 结合本市实际,依托国家人社部、教育部、国家体育总局转型学校教练员培训体系,对符合条件的教练员,且有意投身教育事业的,由市级体育、教育部门统筹组织,开展免费专项培训,帮助其提升教育教学能力。 |
| X7:5 | Others | 0 | — | — |
| X8:1 | Clear objectives | 0 | — | — |
| X8:2 | Sufficient basis | 1 | Art. 2, Implementation (二、工作实施) | 各区县、各学校可根据本地、本校实际情况,进一步细化教练员岗位职责任务,积极推动学校体育专业人才队伍建设,鼓励先行先试,积极探索经验,不断完善学校体育工作机制和考核评价体系。 |
| X8:3 | Scientific plan | 1 | Art. 2(1), Establishment of posts (二、工作实施（一）岗位设置) | 市级以上体育传统特色学校(体育传统项目学校、体育特色学校)、国家高水平体育后备人才基地学校、联办共建区(县)级以上高水平运动队学校、高等院校原则上应在核定的专业技术岗位中设置不少于1个专职教练员岗位。鼓励其他学校从实际出发设置专(兼)职教练员岗位。 |
| X8:4 | Detailed content | 1 | Art. 2, Implementation (二、工作实施) | (四)岗位聘用 1.学校在岗位总量和结构比例内,按照学校岗位设置实施方案,根据按需设岗、公开招聘、择优聘用的原则,开展教练员岗位聘用工作。 2.学校教练员在取得教师资格后可按规定申请转任体育教师,体育教师在取得相应教练员职称后可按规定申… |
| X9:1 | Education | 1 | Title / transmittal notice (标题/发文通知) | 为深入贯彻落实中央全面深化改革委员会第十三次会议审议通过的《关于深化体教融合 促进青少年健康发展的意见》(体发〔2020〕1 号)、《体育总局 中央编办 教育部 人力资源社会保障部关于在学校设置教练员岗位的实施意见》(体人规字〔2023… |
| X9:2 | Sports | 1 | Title / transmittal notice (标题/发文通知) | 为深入贯彻落实中央全面深化改革委员会第十三次会议审议通过的《关于深化体教融合 促进青少年健康发展的意见》(体发〔2020〕1 号)、《体育总局 中央编办 教育部 人力资源社会保障部关于在学校设置教练员岗位的实施意见》(体人规字〔2023… |

First-level values: X1 0.50; X2 0.00; X3 1.00; X4 0.33; X5 1.00; X6 0.67; X7 0.40; X8 0.75; X9 1.00. PMC index 5.65; concavity index 3.35.

## **P18 — Shanghai Municipality**

| **Indicator** | **Meaning** | **Code** | **Locator** | **Quoted warrant** |
| --- | --- | --- | --- | --- |
| X1:1 | Forecasting | 0 | — | — |
| X1:2 | Regulation | 1 | Art. 3, Establishment of posts (三、岗位设置) | 本市青少年高水平运动队(学校二线运动队)所在学校应逐步设置专职教练员专业技术岗位;鼓励体育“一条龙”布局学校和建有高水平优秀运动队的高校逐步按项目设置专职教练员专业技术岗位。 |
| X1:3 | Recommendation | 1 | Art. 3, Establishment of posts (三、岗位设置) | 鼓励体育“一条龙”布局学校和建有高水平优秀运动队的高校逐步按项目设置专职教练员专业技术岗位。 |
| X1:4 | Description | 1 | Title / transmittal notice (标题/发文通知) | 为贯彻落实《中华人民共和国体育法》,根据中共中央办公厅、国务院办公厅《关于全面加强和改进新时代学校体育工作的意见》(中办发〔2020〕36号)、《体育总局 教育部关于印发深化体教融合 促进青少年健康发展意见的通知》(体发〔2020〕1号… |
| X1:5 | Guidance | 1 | Art. 1, Guiding principles (一、指导思想) | 一、指导思想 以习近平新时代中国特色社会主义思想为指导,全面贯彻党的教育方针,坚持“健康第一”教育理念,深入贯彻落实全国教育大会精神及国家、本市加强学校体育工作的一系列指示要求,以提高学生身心健康水平和综合素质、促进学生全面发展和终身发… |
| X1:6 | Others | 1 | Art. 8, Organization and implementation (八、组织实施) | 本意见自发布之日起实施,由市体育局、市委编办、市教委、市人力资源社会保障局负责解释。 |
| X2:1 | Short-term | 0 | — | — |
| X2:2 | Medium-term | 0 | — | — |
| X2:3 | Long-term | 0 | — | — |
| X2:4 | Within current year | 0 | — | — |
| X3:1 | Qualification requirements | 1 | Art. 6, Qualification requirements (六、任职条件) | 六、任职条件 学校教练员在符合事业单位工作人员基本任职条件基础上,还须符合下列条件: |
| X3:2 | Scope of application | 1 | Art. 2, Scope of application (二、适用范围) | 二、适用范围 本意见适用于中小学校(含中等职业学校)、高校。 |
| X3:3 | Professional title system | 1 | Art. 5, Professional title system (五、职称体系) | 五、职称体系 学校教练员职称纳入体育专业人员职称系列,初级、中级、副高级、正高级职称的名称分别为初级学校教练、中级学校教练、高级学校教练、国家级学校教练,职称评价标准另行制定。 |
| X3:4 | Job responsibilities | 1 | Art. 4, Duties of the post (四、岗位职责) | 四、岗位职责 学校教练员按照学校体育工作计划,发挥专业特长,承担体育教学和训练,学生体育运动专项技能、体能训练和体育后备人才选育,学校体育赛事活动组织、学校运动队训练竞赛管理、运动损伤防护康复等知识技能传授,以及学校体育社团管理等工作。 |
| X3:5 | Implementation measures | 1 | Art. 7, Appointment to posts (七、岗位聘用) | 七、岗位聘用 (一)学校按现有规定程序要求制定学校教练员岗位设置实施方案,根据按需设岗、公开招聘、择优聘用的原则,开展岗位聘用工作。 |
| X4:1 | National | 0 | — | — |
| X4:2 | Provincial | 1 | Issuing authority / signature block (发文机关/落款) | 上海市体育局 中共上海市委机构编制委员会办公室 上海市教育委员会 上海市人力资源和社会保障局 沪体人〔2024〕82号 |
| X4:3 | Sub-provincial or affiliated | 0 | — | — |
| X5:1 | Strengthening | 1 | Title / transmittal notice (标题/发文通知) | 加强学校体育工作是深入落实党的教育方针、实施体育强国战略的重要举措,对于促进青少年全面健康发展、实现中华民族伟大复兴具有重要意义。 |
| X5:2 | Guiding | 1 | Art. 5, Organization and implementation (五、组织实施) | 人力资源和社会保障主管部门要加强指导把关,结合年报汇总审核,梳理规范各学校教练员岗位设置相关工作。 |
| X5:3 | Encouraging | 1 | Art. 3, Establishment of posts (三、岗位设置) | 鼓励体育“一条龙”布局学校和建有高水平优秀运动队的高校逐步按项目设置专职教练员专业技术岗位。 |
| X5:4 | Improving | 1 | Art. 7, Appointment to posts (七、岗位聘用) | (六)探索完善体育“一条龙”布局学校优秀教练员共用机制,统筹安排“一条龙”不同学段学校教练员的教学训练任务,推动学校教练员资源共享。 |
| X5:5 | Carrying out | 1 | Art. 7, Appointment to posts (七、岗位聘用) | (一)学校按现有规定程序要求制定学校教练员岗位设置实施方案,根据按需设岗、公开招聘、择优聘用的原则,开展岗位聘用工作。 |
| X5:6 | Promoting | 1 | Title / transmittal notice (标题/发文通知) | 加强学校体育工作是深入落实党的教育方针、实施体育强国战略的重要举措,对于促进青少年全面健康发展、实现中华民族伟大复兴具有重要意义。 |
| X6:1 | Government | 1 | Art. 6, Qualification requirements (六、任职条件) | (六)具备专项运动的专业理论水平和技术能力,具有本市体育和教育部门认可的教练员从业经历或体育项目专业训练经历。 |
| X6:2 | Schools | 1 | Title / transmittal notice (标题/发文通知) | 市体育局 市委编办 市教委 市人力资源社会保障局关于印发《关于上海市学校教练员专业技术岗位设置管理的实施意见》的通知 |
| X6:3 | Society | 1 | Art. 3, Establishment of posts (三、岗位设置) | 学校也可通过向社会专业机构等购买体育训练服务、聘请兼职教师等方式加强专业训练师资力量。 |
| X7:1 | Supervision and inspection | 1 | Art. 8, Organization and implementation (八、组织实施) | 各级行政主管部门要加强统筹协同,定期组织教练员岗位管理会商协调会,分析工作进展和成效,及时研究解决新情况新问题,切实将岗位设置的各项工作落实到位,将学校教练员岗位管理工作纳入督导评估范围。 |
| X7:2 | Organizational leadership | 1 | Art. 8, Organization and implementation (八、组织实施) | 各级行政主管部门及各学校要充分认识学校教练员人才队伍建设的重要意义,切实加强组织领导,建立有效工作机制,推动学校教练员岗位设置和人员配备落实到位。 |
| X7:3 | Publicity and guidance | 0 | — | — |
| X7:4 | Talent support | 1 | Art. 3, Establishment of posts (三、岗位设置) | 设立专职教练员岗位的学校,在核定的编制和专业技术岗位总量及结构比例内设置,专岗专用,纳入专业技术岗位进行管理。中小学校、高校主管部门可对所管理学校的教练员岗位统筹设置,统一管理使用。 |
| X7:5 | Others | 1 | Art. 8, Organization and implementation (八、组织实施) | 本意见自发布之日起实施,由市体育局、市委编办、市教委、市人力资源社会保障局负责解释。 |
| X8:1 | Clear objectives | 0 | — | — |
| X8:2 | Sufficient basis | 1 | Title / transmittal notice (标题/发文通知) | 为贯彻落实《中华人民共和国体育法》,根据中共中央办公厅、国务院办公厅《关于全面加强和改进新时代学校体育工作的意见》(中办发〔2020〕36号)、《体育总局 教育部关于印发深化体教融合 促进青少年健康发展意见的通知》(体发〔2020〕1号… |
| X8:3 | Scientific plan | 1 | Art. 2, Establishment of posts (二、岗位设置) | 本市青少年高水平运动队(学校二线运动队)所在学校应逐步设置专职教练员专业技术岗位;鼓励体育“一条龙”布局学校和建有高水平优秀运动队的高校逐步按项目设置专职教练员专业技术岗位。其他中小学校、高校可根据学校体育工作需要设置专(兼)职教练员专业技术岗位。鼓励民办学校设置专(兼)职教练员专业技术岗位。 |
| X8:4 | Detailed content | 1 | Art. 6, Qualification requirements (六、任职条件) | 六、任职条件 学校教练员在符合事业单位工作人员基本任职条件基础上,还须符合下列条件: (一)具有良好的思想政治素质和道德品质,遵纪守法,遵守职业道德规范,身心健康,举止文明。 (二)热爱教育事业,为人师表,关爱学生,遵循教育规律和学生成… |
| X9:1 | Education | 1 | Title / transmittal notice (标题/发文通知) | 各区体育局、区委编办、区教育局、区人力资源社会保障局,各有关单位: |
| X9:2 | Sports | 1 | Title / transmittal notice (标题/发文通知) | 市体育局 市委编办 市教委 市人力资源社会保障局关于印发《关于上海市学校教练员专业技术岗位设置管理的实施意见》的通知 |

First-level values: X1 0.83; X2 0.00; X3 1.00; X4 0.33; X5 1.00; X6 1.00; X7 0.80; X8 0.75; X9 1.00. PMC index 6.72; concavity index 2.28.

# **S4.3 Adjudication and verification summary**

The pre-adjudication blind re-coding and agreement statistics are summarised in Supplementary Material S2. Adjudication changed 32 of the 456 item cells: 27 from 1 to 0 and 5 from 0 to 1. The number of positive codes therefore decreased from 343 to 321.

The coding sheet in S4.2 is the final analysed dataset and is the source of the first-level values and PMC indices reported in the manuscript. Each of the 321 positive codes was checked against the corresponding policy text; every quoted warrant was required to match the cited policy text after normalising only whitespace and full-/half-width character forms.

Because the study used one coder, the reliability evidence is intra-coder test–retest consistency rather than inter-coder reliability. Publication of the complete final coding sheet allows readers to inspect individual coding judgments directly.
